# Exclusion rates in randomized controlled trials of treatments for physical conditions: a systematic review

**DOI:** 10.1186/s13063-020-4139-0

**Published:** 2020-02-26

**Authors:** Jinzhang He, Daniel R. Morales, Bruce Guthrie

**Affiliations:** 1 Ninewells Hospital and Medical School, University of Dundee, James Arrott Drive, Dundee, DD2 1SY Scotland; 2 Population Health and Genomics Division, University of Dundee, Mackenzie Building, Kirsty Semple Way, Dundee, DD2 4BF Scotland; 3 Centre for Population Health Sciences, University of Edinburgh, Doorway 3 Old Medical School, Teviot Place, Edinburgh, EH8 9AG Scotland

**Keywords:** Randomized controlled trial [V03.175.250.500.500], External validity, Generalizability, Multimorbidity [N05.715.350.225.500], Aged [M01.060.116.100], Systematic review [V03.850], Real-world evidence

## Abstract

**Background:**

The generalisability of randomized controlled trials (RCTs) can be uncertain because the impact of exclusion criteria is rarely quantified. The aim of this study was to systematically review studies examining the percentage of clinical populations with a physical health condition who would be excluded by RCTs of treatments for that condition.

**Methods:**

Medline and Embase were searched from inception to Feb 11th 2018. Two reviewers independently completed screening, full-text review, data extraction and risk-of-bias assessment. The primary outcome was the percentage of patients in the clinical population who would have been excluded from each examined trial. Subgroup analyses examined exclusion by population setting, publication date and funding source.

**Results:**

Titles/abstracts (20,754) were screened, and 50 studies were included which reported exclusion rates from 305 trials of treatments in 31 physical conditions. Estimated rates of exclusion from trials varied from 0% to 100%, and the median exclusion rate was 77.1% of patients (interquartile range 55.5% to 89.0% exclusion). Median exclusion rates for trials in common chronic conditions were high, including hypertension 83.0%, type 2 diabetes 81.7%, chronic obstructive pulmonary disease 84.3%, and asthma 96.0%. The most commonly applied exclusion criteria related to age, co-morbidity and co-prescribing, whereas more implicit criteria relating to life expectancy or functional status were not typically examined. There was no evidence that exclusion varied by the nature of the clinical population in which exclusion was evaluated or trial funding source. There was no statistically significant change in exclusion rates in more recent compared with older trials.

**Conclusions:**

The majority of trials of treatments for physical conditions examined excluded the majority of patients with the condition being treated. Almost a quarter of the trials studied excluded over 90% of patients, more than half of trials excluded at least three quarters of patients, and four out of five trials excluded at least half of patients. A limitation is that most studies applied only a subset of eligibility criteria, so exclusion rates are likely under-estimated. Exclusion from trials of older people and people with co-morbidity and co-prescribing is increasingly untenable given population aging and increasing multimorbidity.

**Trial registration:**

PROSPERO registration CRD42016042282.

## Background

Randomized controlled trials (RCTs) are the gold standard method for evaluating the efficacy of treatments because well-designed RCTs minimize bias and confounding. They therefore maximize internal validity, giving confidence that the results are true for the trial population studied. However, trial populations are often highly selected, which may weaken the generalizability of RCT evidence in the sense of leaving uncertainty that the results apply to everyone with the condition in clinical practice [1, 2]. Some exclusions from RCTs are justifiable (e.g., where an individual is allergic to a medicine). However, Van Spall et al. estimated that 84.1% of trials published in high-impact general medical journals between 1994 and 2006 had poorly justified patient exclusion criteria [3].

A number of studies have shown that various landmark RCTs measuring treatment effects, many of which underpin guideline recommendations and influence regulatory decision-making, exclude large proportions of people with the condition being treated [4, 5]. Older people, women, and people with co-morbidity or co-prescribing are noticeably excluded from trials [3, 6, 7]. Although there is some evidence that women and older people are better represented in newer trials, they remain under-represented compared with the wider population [7]. These patterns of exclusion do not represent the realities of current and future clinical practice. Most people with any chronic condition have co-morbidity, and multimorbidity is the norm in older people [8, 9]. Therefore, guideline-recommended treatment in routine practice will often require significant extrapolation from RCT evidence [10, 11], where strict RCT eligibility criteria lead to trial populations significantly differing from clinical populations seen in routine practice [12, 13].

The problem that strict RCT eligibility criteria pose for generalizing from RCT-derived evidence is well known [14, 15]. However, the extent to which trials assessing treatment effects across different conditions exclude patients seen and treated in clinical practice is uncertain. The aims of this study were to undertake a systematic review of studies estimating the percentage of people with a chronic physical condition who would be excluded by RCTs of treatment for that condition and to examine how exclusion rates varied for different diseases, for different clinical populations, and over time.

## Methods

### Search strategy

A systematic review was undertaken searching the Medline and Embase databases from inception to 11 February 2018 for all studies comparing the percentage of people from a ‘clinical’ population with a physical condition who would have been excluded from one or more trials of treatment intended for that condition. The search strategy is detailed in Additional file 1.

### Inclusion criteria

We included studies published that explicitly examined the percentage of people with a chronic physical condition in a defined clinical population who would have been eligible for one or more selected RCTs of an individual patient treatment for that condition (including medication, surgery and other non-pharmacological interventions). The clinical populations included were not restricted in terms of their setting or method of sampling and therefore could be any of unselected patients seen in clinical practice in primary or specialist care, patients in clinical or research registries, or research cohorts identified or recruited in these settings. However, the appropriateness of the clinical population used to examine exclusion from a particular trial was examined as part of risk-of-bias evaluation.

### Exclusion criteria

We excluded studies examining eligibility for trials of mental health conditions, studies that were not published in English, studies that did not explicitly report the percentage of patients eligible for trials or where percentages of patients eligible could not be calculated from the available data (e.g., those comparing recruited with non-recruited patients without examining exclusion in an underlying clinical population), and studies examining eligibility for a hypothetical trial or applying a set of common exclusion criteria from multiple trials instead of using actual exclusion criteria from single trials. Since estimated exclusion rates in very small clinical populations are likely to be imprecise, we also excluded studies where eligibility was calculated in a clinical population that included fewer than 100 patients.

### Selection of studies

All titles and abstracts were independently screened by two reviewers to identify papers for full-text review. Full-text review and data extraction were carried out independently by two reviewers on the basis of the published protocol [16], and disagreements were resolved by discussion to reach consensus.

### Data extraction and quality assessment

Data extraction was carried out by a minimum of two reviewers, involving a third reviewer where necessary, and disagreements were resolved by discussion to reach consensus. Data extracted for each study included the condition of interest and a description of comparison clinical population, including the purpose of the clinical population dataset (e.g., clinical registry and electronic health record data), health-care setting and location, the date of clinical population recruitment or identification, clinical population size, and the diagnostic criteria used to define the clinical population. These data were used to make an assessment of bias on the overall appropriateness of the clinical population. Extracted data for the underlying trials examined by each study included the rationale for the choice of trials examined, the type of intervention or treatment in the trial, the listed trial eligibility criteria that were applied (or not) to each clinical population to estimate exclusion rate, and the trial’s source of funding (pharmaceutical versus non-pharmaceutical).

The primary outcome extracted was the percentage of patients in the clinical population who would have been excluded for each trial examined and the reported 95% confidence interval (CI) of this percentage (which was calculated if not reported by the authors).

### Risk-of-bias assessment

There is no published risk-of-bias tool to assess the kinds of studies examined. We therefore developed three pre-specified risk-of-bias criteria that were independently assessed by two reviewers, namely:

1. *How the reviewed paper selected trials to examine*. We evaluated whether there was a systematic approach to trial selection (e.g., systematic search of the literature) or a clearly stated justification for the choice of trials and whether that justification was judged to be adequate. Studies were considered to be at low risk of bias if selection rationale were clearly stated and judged to be justifiable; otherwise, they were considered to be at high risk of bias.
2. *The appropriateness of each trial–clinical population pair*. The appropriateness of each trial–clinical population pair was assessed in relation to how well the clinical population appropriately represented the population for whom the treatment evaluated in the trial was intended or suitable. For example, a primary care population of people with heart failure is appropriate for a trial of beta-blockers or angiotensin-converting enzyme inhibitors used as long-term treatment [4], whereas an emergency department population is appropriate for a trial of treatment in acute, decompensated heart failure [17]. Studies were considered to be at low risk of bias if the clinical population was judged to be representative of real-world populations for which the trial treatment was intended or indicated, at high risk of bias if the clinical population was not considered to be representative of real-world populations for which the trial treatment was intended or indicated, and at unclear risk of bias if insufficient information was provided for assessment.
3. *The choice of trial eligibility criteria to examine*. The choice of trial eligibility criteria assessed in relation to the stated criteria applied and not applied. Studies were considered to be at low risk of bias for the choice of trial eligibility criteria assessed in relation to the stated criteria if they clearly stated that all important or common criteria were applied; otherwise, studies were considered to be at high risk of bias.

### Data synthesis and analysis

Some trials were evaluated in more than one clinical population. In this situation, the trial–clinical population pair with the lowest percentage of patients was selected for analysis in order to obtain the most conservative estimate of the percentage of patients excluded. For the remaining trial–clinical population pairs, the overall median, range and interquartile range for the primary outcome (the estimated percentage of the clinical population excluded by each trial) were calculated and repeated for condition groups (cardiovascular conditions, diabetes, respiratory conditions, cancer, rheumatoid arthritis (RA), human immunodeficiency virus (HIV) and other conditions) and for individual conditions. Variation was further examined by using linear regression to model unadjusted and adjusted differences in the percentage excluded by each trial in relation to whether the clinical population was recruited from primary or specialist care, whether the trial examined was publicly funded or industry-funded, the date of trial publication (with trials grouped into quartiles of publication date with equal numbers of trials in each group: 1994–1999, 2000–2003, 2004–2011 and 2012–2018), and risk-of-bias assessment (low risk of bias versus high or unclear risk of bias).

## Results

### Study characteristics

**Fig. 1:**
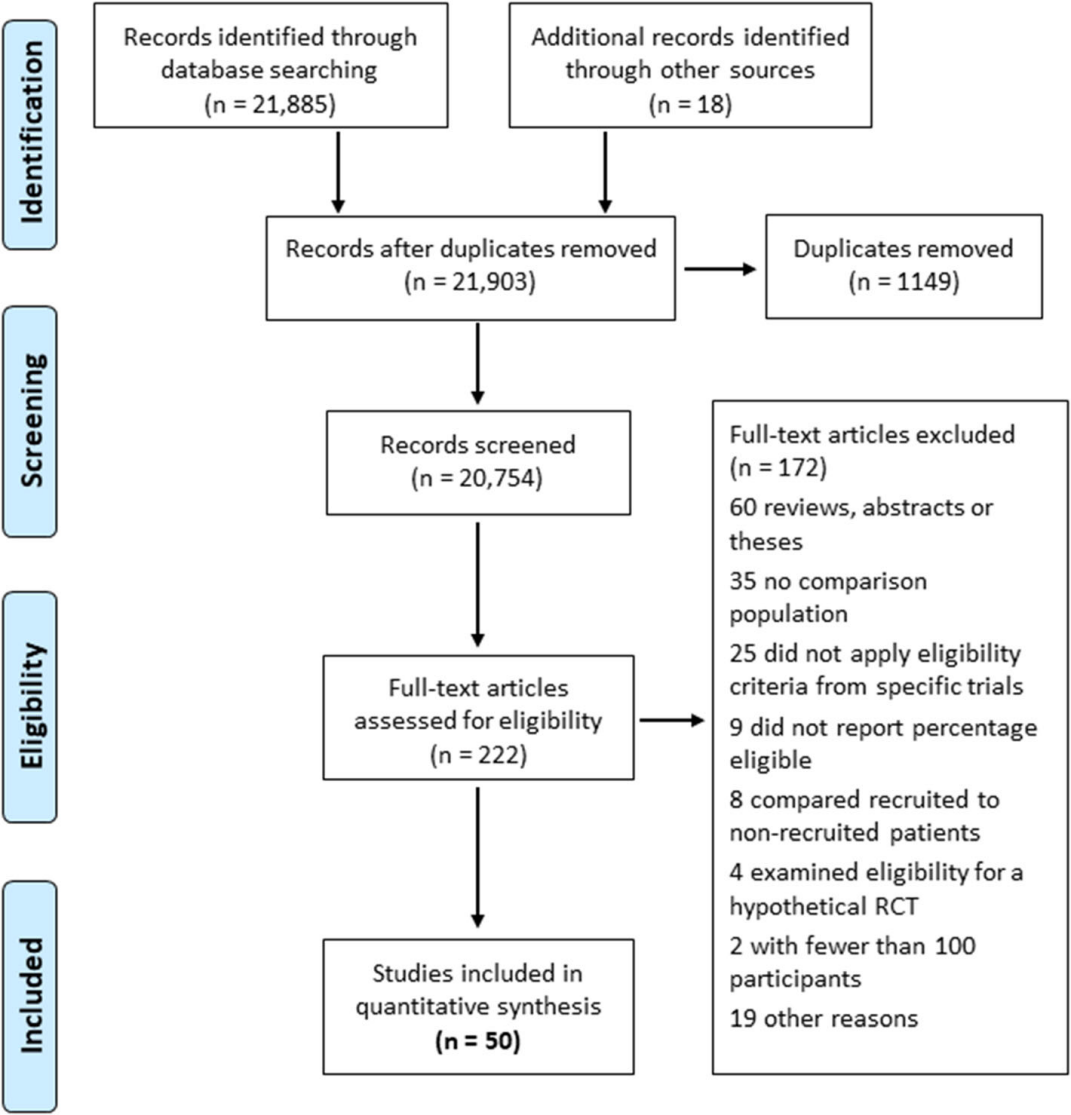
Flow diagram of identification, screening and eligibility assessment

The searches identified 21,885 articles with a further 18 identified from other sources, including examination of references of included studies. Non-duplicate documents (20,754) were screened, and 222 full-text articles were examined. Fifty studies that examined trial eligibility in 57 distinct clinical populations were included (Fig. 1). Twenty of the reference clinical populations examined were primary care or community samples: seven derived from electronic clinical datasets, three clinical registries, five research registries, and five survey-derived populations. Thirty-seven of the clinical populations examined were specialist samples: 19 derived from record review of various kinds, four clinical registries, and 14 research registries. Characteristics of all 50 included studies and all 57 reference clinical populations are shown in supplementary table S1. The 50 studies provided data on the proportion of the reference clinical population that would have been excluded by 305 trials. Characteristics of all the trials examined by these studies are shown in supplementary tables S2, S3, S4, S5, S6, S7, S8, S9, S10, S11, S12, S13, S14, S15 and S16. Eighty-one (26.6%) of the trials examined by the 50 studies were published in 1994–1999, 78 (25.6%) in 2000–2003, 75 (24.6%) in 2004–2011, and 67 (22.0%) in 2012–2018. Seventy-five (24.6%) trials were publicly funded, 203 (66.6%) industry-funded, and 27 (8.9%) did not record their source of funding. Included studies examined trial exclusion in 31 physical conditions (seven cardiovascular, three diabetes, three respiratory, eight types of cancer, RA, HIV infection, and eight other conditions) (Table 1), and there was considerable heterogeneity in the treatments being trialed (supplementary table S2). The trials examined were most frequently evaluating treatments for RA (51 trials; 16.7%), chronic obstructive pulmonary disease (COPD) (51 trials; 16.7%), HIV infection (31 trials; 10.2%), heart failure (25 trials; 8.2%) and hypertension (22 trials; 7.2%).

**Table 1:** Percentage of the clinical population excluded by condition studied

	Number of trials	Median percentage excluded (range excluded)^a^
**All conditions**	305	77.1 (0.0 to 100.0)
**Cardiovascular conditions**	81	74.7 (1.6 to 98.8)
Heart failure	25	65.0 (18.8 to 92.0)
Hypertension	22	83.0 (1.6 to 98.8)
Stroke/transient ischemic attack	21	83.6 (33.2 to 98.4)
Atrial fibrillation	4	34.9 (32.3 to 41.2)
Coronary heart disease	4	53.1 (2.8 to 84.5)
Lipid lowering for primary prevention	4	85.9 (69.7 to 89.1)
Secondary prevention of myocardial infarction	1	76.8
**Diabetes mellitus**	16	88.1 (29.8 to 99.0)
Type 2 diabetes	7	81.7 (49.3 to 96.5)
Diabetic ulcers	7	93.3 (29.8 to 99.0)
Type 1 diabetes	2	91.6 (87.5 to 95.6)
**Respiratory conditions**	78	89.4 (42.4 to 100.0)
COPD	51	84.3 (42.4 to 100.0)
Asthma	17	96.0 (64.0 to 100.0)
Bronchiectasis	10	80.1 (49.0 to 93.0)
**Cancer**	24	56.6 (13.6 to 81.2)
Breast cancer	12	56.6 (28.9 to 81.2)
Lung cancer	3	71.4 (65.4 to 71.9)
Renal cancer	3	13.6 (13.6 to 48.5)
Colorectal cancer	2	66.7 (65.7 to 67.6)
Bladder cancer	1	45.3
Stomach cancer	1	41.3
Lymphoma	1	70.4
Prostate cancer	1	57.1
**Rheumatoid arthritis**	51	84.0 (56.0 to 98.7)
**HIV infection**	32	42.0 (0.0 to 67.6)
**Other conditions**	23	58.3 (23.7 to 88.9)
Venous thromboembolism prophylaxis	9	41.5 (23.7 to 78.8)
Venous ulcers	7	83.6 (58.3 to 88.9)
Brain injury	2	40.5 (35.9 to 45.0)
Pressure ulcers	1	34.7
Alzheimer’s disease	1	86.5
Fibromyalgia	1	52.1
Irritable bowel syndrome	1	73.1
Incisional hernia	1	62.5

*Abbreviations*: *COPD* chronic obstructive pulmonary disease, *HIV* human immunodeficiency virus

^a^ Where there is only one trial–clinical population comparison, the number reported is the value for that comparison; where there are two, the median reported is the midpoint value between the two

### Percentage of the clinical population excluded from trials

**Fig. 2:**
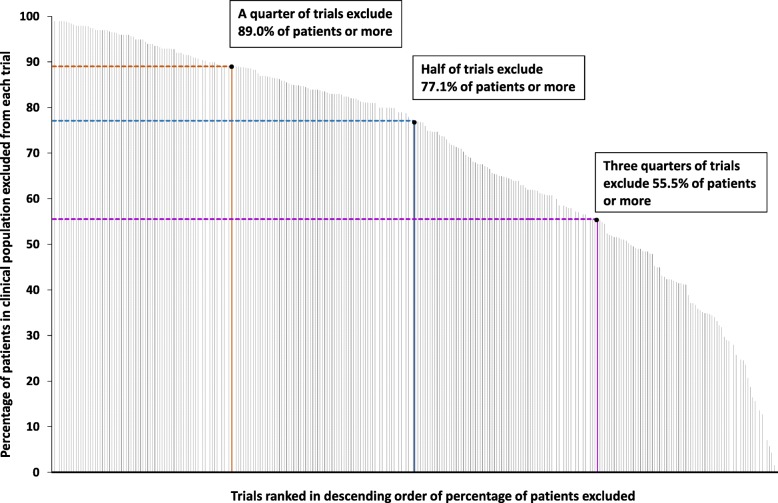
Trials ranked in descending order of the percentage excluded in the clinical population studied

Across all 305 trials, the median rate of exclusion was 77.1% (range 0–100%) of patients, varying from a median of 42.0% for HIV trials to a median of 89.4% for respiratory trials (Table 1, Fig. 2). Only 16 (5.2%) trials excluded less than 25% of patients, whereas 159 (52.1%) excluded at least 75%. At single-condition level, trials of treatments in atrial fibrillation excluded the fewest patients (median 34.9%, range 32.3–41.2%) and trials of treatments in asthma the most (median 96.0%, range 64.0–100%). Notably, exclusion rates for the most common chronic conditions were high, including hypertension 83.0%, lipid-lowering drugs in primary prevention 85.9%, type 2 diabetes 81.7%, COPD 84.3% and asthma 96.0%.

### Inclusion and exclusion criteria used by studies to estimate exclusion rates

It was only explicit which eligibility criteria had been used to determine exclusion rates in the clinical population for 174 (57.4%) of trials. The most commonly reported eligibility criterion used to determine exclusion rates was disease severity for 142 trials (81.4% of trials where this was reported), most commonly selecting patients with more severe or less well-controlled disease. Co-morbidity was reported as being used to determine exclusion rates for 119 (68.4%) trials, usually as an exclusion criterion (117 [67.2%] trials) but sometimes as an inclusion criterion (14 [8.0%] trials, for example, to select patients at higher risk of cardiovascular disease in diabetes and atrial fibrillation trials). Age was reported as used to determine exclusion rates in the clinical population for 86 (49.4%) trials, most commonly using an upper age limit for eligibility and other criteria such as limited life expectancy and inability to comply with treatment for 56 (32.2%) trials.

### Variation by type of condition

HIV trials excluded the lowest percentage of patients amongst the different conditions (mean 38.4%, 95% CI 31.4 to 45.5) (Table 2). In unadjusted analysis, there were statistically significantly higher rates of exclusion for all other conditions compared with HIV trials, and cancer trials excluded 15.6% more patients, cardiovascular trials 31.8% more, respiratory trials 36.6% more, RA trials 44.6% more, and diabetes trials 42.4% more. When adjusted for all other variables, the results remained similar with significant differences in the percentage of patients excluded in trials of different conditions compared with HIV trials; cancer trials excluded 20.4% (95% CI 8.8 to 48.4) more patients, cardiovascular trials 34.0% (95% CI 24.0 to 44.0) more, respiratory trials 43.1% (95% CI 31.9 to 54.4) more, RA trials 43.9% (95% CI 33.4 to 54.4) more, and diabetes trials 46.8% (95% CI 31.1 to 62.6).

**Table 2:** Exclusion rates by trial characteristics

Variable (number of trials)	Unadjusted coefficient (95% CI)^a^	*P* value	Adjusted coefficient (95% CI)^a^	*P* value
Condition				
HIV infection (*n* = 32)	Reference		Reference	
Cancer (*n* = 24)	15.6 (5.0 to 26.2)	< 0.001	20.4 (8.8 to 32.0)	< 0.001
Cardiovascular (*n* = 81)	31.8 (23.8 to 39.7)	0.003	34.0 (24.0 to 44.0)	< 0.001
Respiratory (*n* = 78)	36.6 (27.8 to 45.3)	< 0.001	43.1 (31.9 to 54.2)	< 0.001
Rheumatoid arthritis (*n* = 51)	44.6 (36.9 to 52.2)	< 0.001	43.9 (33.4 to 54.4)	< 0.001
Diabetes (*n* = 16)	42.4 (28.2 to 56.7)	< 0.001	46.8 (31.1 to 62.6)	< 0.001
Other conditions (*n* = 23)	19.5 (9.2 to 29.8)	< 0.001	25.0 (12.2 to 37.8)	< 0.001
Trial funding source^b^				
Public (*n* = 75)	Reference		Reference	
Industry (*n* = 203)	15.7 (9.6 to 21.7)	< 0.001	−4.7 (−11.0 to 1.6)	0.1
Comparison clinical population setting				
Primary care (*n* = 198)	Reference		Reference	
Specialist care (*n* = 107)	−6.2 (−11.7 to −0.6)	0.03	−3.0 (−9.0 to 3.0)	0.3
Year of trial publication				
1994–1999 (*n* = 81)	Reference		Reference	
2000–2003 (*n* = 78)	−4.0 (−11.4 to 3.3)	0.28	−4.7 (−10.8 to 1.4)	0.1
2004–2011 (*n* = 75)	− 1.1 (−8.6 to 6.3)	0.76	− 6.2 (−13.1 to 0.7)	0.08
2012–2018 (*n* = 71)	−0.3 (−7.4 to 7.9)	0.95	−6.5 (−13.8 to 0.7)	0.08
Risk of bias				
Low (*n* = 126)	Reference		Reference	
High/Unclear (*n* = 179)	17.2 (12.2 to 22.2)	< 0.001	9.2 (3.5 to 14.8)	0.002

*Abbreviations*: *CI* confidence interval, *HIV* human immunodeficiency virus

^a^ The coefficients are interpreted as the percentage point difference in exclusion in each category compared with the reference

^b^ Twenty-seven trials did not report funding source

### Variation by funding source, clinical population and trial publication date

Publicly funded trials excluded a mean of 58.2% of patients (95% CI 52.3 to 64.1), and industry-funded trials excluded 15.7% more (95% CI 9.6 to 21.7) in unadjusted analysis, but there was no statistically significant difference observed after adjustment (difference −4.7%, 95% CI −11.0 to 1.6). Studies where the clinical population was recruited in primary care excluded a mean of 72.2% (95% CI 69.0 to 75.5). In unadjusted analysis, studies where the clinical population was in specialist care excluded 6.2% (95% CI −11.7 to −0.6) more patients, but there was no statistically significant difference after adjustment (difference −3.0%, 95% CI −9.0 to 3.0). Trials published during 1994–1999 excluded 71.0% of patients (95% CI 65.6 to 76.5) on average. This was no different in later time periods in unadjusted analysis. Although estimated differences were larger in adjusted analysis with fewer people excluded more recently, differences remained non-significant (2012–2018 difference compared with 1194–1999 −6.5% (95% CI −13.8 to 0.7, *P* = 0.08).

### Risk of bias

In risk-of-bias assessment, 126 (41.3%) of estimates of trial exclusion rates were assessed as low risk of bias, 104 (34.1%) as high risk, and 75 (24.6%) as unclear. High risk of bias was driven largely by the clinical population used in the comparison being judged as less appropriate for the treatment being trialed (supplementary tables S17 and S18). Comparisons with a low risk of bias had significantly lower exclusion rates (Table 2). Low-risk studies excluded 59.9% (95% CI 55.7 to 64.1) of patients on average, and studies rated high/unclear risk of bias excluded 17.2% more patients (95% CI 12.2 to 22.2). After adjustment for other characteristics, studies rated high/unclear risk of bias excluded 9.2% more patients (95% CI 3.5 to 14.8).

### Trials where exclusion rates were estimated in multiple clinical populations

Thirty-eight trials were examined in two or more clinical populations (Table 3), and 30 were trials of treatment for RA. Exclusion rates of nine RA trials were each estimated in three clinical populations [18], whereas exclusion rates for the remaining 21 were estimated in two clinical populations [19]. For the nine trials examined in three clinical populations, estimated exclusion rates were higher in every comparison in the Veterans’ Affairs Rheumatoid Arthritis (VARA) cohort (median 97.4%, range 75.6 to 98.4%) when compared with the Rheumatoid Arthritis Investigators’ Network (RAIN) database (median 89.6%, range 74.7 to 91.6%) and the National Register for Biologic Treatment cohort (median 80.0%, range 56.0 to 92.4%). In the remaining 21 trials, estimated exclusion rates in every comparison were higher in VARA (median 97.4%, range 72.7 to 99.1%) than in RAIN (median 89.0%, range 64.9 to 93.5%). Such differences would be expected given variation in the data collected by different registries and in the clinical population included (VARA, for example, is made up predominately of male veterans whereas RAIN is a less selected population of patients attending rheumatology clinics) [20]. Differences were more variable and sometimes larger for trials of treatments for the other conditions (atrial fibrillation, heart failure, acute myocardial infarction and COPD) examined in more than one clinical population, although there was no consistent pattern to explain this in relation to risk of bias or the nature of the clinical population (Table 3).

**Table 3:** Consistency of findings when the same trial is examined in more than one clinical population

Condition and trial	Clinical population	Overall risk of bias	Percentage excluded Median percentage (range for Aaltonen and Vashisht)
Atrial fibrillation ARISTOTLE (2011)	Yoon (record review for consenting patients in a single hospital)^a^	High	32.3
	Lee (primary care electronic medical record population data)	Low	38.7
	Fanning (record review within multiple hospitals)	High	39.5
	Desmaele (clinical registry in a single hospital)	High	54.5
	Hagg (primary care electronic medical record population data)	Low	71.1
Atrial fibrillation ROCKET-AF (2011)	Yoon (record review for consenting patients in a single hospital)^a^	High	34.5
	Lee (primary care electronic medical record population data)	Low	52.5
	Desmaele (clinical registry in a single hospital)	High	60.7
	Fanning (record review within multiple hospitals)	High	64.2
Atrial fibrillation RE-LY (2009)	Yoon (record review for consenting patients in a single hospital)^a^	High	35.2
	Lee (primary care electronic medical record population data)	Low	36.2
	Fanning (record review within multiple hospitals)	High	47.4
	Desmaele (clinical registry in a single hospital)	High	52.4
Heart failure MERIT-HF (2000)	Constantino (record review in a single hospital)^a^	Unclear	48.0
	Jost (clinical registry in a single hospital)	Low	58.8
	Masoudi (National Heart Failure Project registry)	High	82.6
Heart failure RALES (1999)	Masoudi (National Heart Failure Project registry)^a^	High	74.7
	Costantino (record review in a single hospital)	Unclear	76.0
Acute myocardial infarction GUSTO (1993)	Krumholz (National Research Registry of Myocardial Infarction)^a^	High	84.5
	Krumholz (Cooperative Cardiovascular Project registry)	High	90.6
COPD POET-COPD (2011)	Kruis (seven primary care databases)^a^	High	77.0
	Halpin (primary care research database)	Low	88.2
COPD UPLIFT (2009)	Kruis (seven primary care databases)^a^	High	58.0
	Halpin (primary care research database)	Low	77.5
Rheumatoid Arthritis 9 trials^b^	Aaltonen (National Register for Biologic Treatment)^a^	High	80.0 (56.0 to 92.4)
	Vashisht (RA Investigators’ Network database research registry)	High	89.6 (74.7 to 91.6)
	Vashisht (Veterans’ Affairs Rheumatoid Arthritis research registry)	Unclear	97.4 (75.6 to 98.4)
Rheumatoid Arthritis 21 trials^b^	Vashisht (RA Investigators’ Network database research registry)^a^	Unclear	89.0 (64.9 to 93.5)
	Vashisht (Veterans’ Affairs Rheumatoid Arthritis research registry)	High	97.4 (72.7 to 99.1)

^a^ Marked trials with most conservative estimate of percentage of patients excluded were analysed

^b^ See supplementary table S14 for individual trial comparisons

## Discussion

### Summary of evidence

This study examined estimated exclusion rates in clinical populations in 305 trials of treatments for physical conditions. Almost a quarter of the trials studied excluded 90% or more of patients, more than half of trials excluded more than 75% of patients, and four out of five trials excluded more than 50% of patients. There was variation in exclusion depending on the condition studied, but exclusion rates did not differ between studies using primary versus specialist care clinical populations to evaluate exclusion rates or between trials that were publicly versus industry-funded. There was no strong evidence that rates of exclusion had changed over time. A third of studies were at high risk of bias, most commonly because the clinical population used was not appropriate for the trial examined, and a further quarter of studies were at unclear risk of bias. Exclusion rates were lower for studies at low risk of bias where median exclusion was 60.8%, although two thirds of low risk-of-bias studies would still have excluded more than 50% and one third more than 75% of patients.

### Strengths and limitations

A strength of the study is the systematic approach to identify and examine the underlying literature by using a deliberately broad search strategy to maximize sensitivity. However, the nature of the literature examined and the fact that there are no clear reporting criteria for such studies make it possible that some studies were not identified. Despite this, estimated exclusion rates in 305 trials in 57 clinical populations were included. A key observation is that examined studies were heterogeneous in a variety of ways. Underlying studies varied in how they selected trials to compare, in their choice of clinical population, and in the trial inclusion and exclusion criteria they applied. Some of the observed variation in exclusion rates likely reflects the choices made, but these were not always explicit in the included studies. This may be related to the fact that there are no clear criteria for the conduct of such studies. A further limitation is that we excluded comparisons with fewer than 100 patients in order to avoid imprecise estimates for common conditions (although, in practice, only two studies were excluded as a result). Finally, most of the underlying studies applied only a subset of eligibility criteria, most commonly age, co-morbidity and co-prescribing because these are easily applied to the data contained in coded data extracted from electronic health records and clinical or research registries. The implication is that true exclusion rates are likely even higher than reported here because of unexamined explicit criteria and because trial recruitment also involves the application of implicit criteria by researchers (such as the presence of frailty and whether an individual is perceived to be likely to adhere to trial procedures).

### Comparison with other literature

Exclusion and inclusion criteria are not always clearly reported in trial publications. For example, 56% of 255 cancer RCTs published in leading journals had discrepancies between eligibility criteria listed in protocols and those listed in the papers reporting results, and 96.7% of these discrepancies imply that the trial population was broader than it actually was [21]. Examining RCTs published in high-impact journals 1994–2006, Van Spall et al. found co-morbidity, age and co-prescribing used as exclusion criteria in the majority of the 283 trials examined, usually without any explicit justification [3]. A study of 4341 RCTs published in four high-impact general medical journals found that 29% had upper age limits for inclusion that were rarely explicitly justified. Although the percentage of trials with upper age limits declined somewhat between 1998 and 2015, absolute change over time was small [22], and only 7% of RCTs published in 2012 were specifically conducted in older patients [23]. Of 319 ongoing RCTs for 10 common conditions registered with ClinicalTrials.gov in 2014, 79% excluded patients with common co-morbidities [24]. Studies of trials in individual conditions have similar findings. Only one of 112 RCTs of secondary prevention of cardiovascular disease published in 2010–2012 justified the exclusion criteria applied [25]. Two thirds of RCTs for type 2 diabetes had upper age limits for inclusion, three quarters excluded a range of co-morbidities, and only 1.4% of the 440 RCTs examined were specifically in older adults [26]. However, this literature does not quantify the impact of inclusion and exclusion criteria on eligibility as we have done here.

### Implications for policy, practice and research

Exclusion of patients from trials matters only if the exclusion criteria are effect modifiers of treatment [27], meaning that the benefits or harms of treatment (or both) systematically vary in the included versus the excluded. This review found that trial evidence is typically derived from narrow populations which are usually selected to have higher risk of outcomes expected to be improved by treatment (e.g., by selective *inclusion* of patients at high cardiovascular risk) and usually selected to have lower risk of adverse effects (e.g., by selective *exclusion* of patients with co-morbidity, co-prescribing and frailty).

Guideline developers, medicine regulators and clinicians therefore all face the problem of having to extrapolate RCT findings to excluded clinical populations where benefits and harms may be plausibly different. Simple extrapolation requires making assumptions that the benefits and harms of treatment are similar in included and excluded populations [28]. This is often reasonable but such assumptions do not always hold true. For example, trial-derived estimated numbers needed to treat (NNTs) for the use of angiotensin-converting enzyme inhibitors over about 3 years to prevent end-stage renal disease (ESRD) in chronic kidney disease are 9–25. Estimated NNTs to prevent ESRD in clinical populations are more than 100 because of lower baseline risk of ESRD and higher risk of competing mortality than observed in trial populations [29]. Adverse effects and harms from treatment are also usually higher in people with frailty and polypharmacy [30] and increase with age. Aspirin used after a cerebrovascular event in patients over 75 years old, for example, is associated with a fivefold increase in fatal bleeding compared with younger patients [31]. So even if treatment benefits are similar in trial and clinical populations, overall net benefit may still vary.

Careful attention to internal validity has improved the quality of trial evidence and its systematic synthesis, but generalizability and applicability are usually less explicitly considered [32]. Despite recommendations that systematic reviews should always discuss applicability of evidence [33], only a minority actually do [34]. There remains a clear place for efficacy trials in highly selected populations, but choosing to design such a trial is also effectively a declaration that the trialists have concerns that net benefit may be different in excluded populations. While more restrictive eligibility criteria for early-stage clinical trials may be appropriate when little is known about a treatment’s safety and efficacy, enrolment of more diverse populations for later studies (or adaptive enrolment to include broader populations depending on initial efficacy findings) will help ensure a better understanding of the treatment’s effect for all patients likely to benefit. In this regard, the US Food and Drug Administration is exploring recommendations around modernizing eligibility criteria for cancer clinical trials [35]. Furthermore, robust methods aimed at generating real-world evidence may help augment evidence from trials.

To facilitate judgements about applicability by clinicians, systematic reviewers, guideline developers and medicine regulators, journals and registries should require trialists to explicitly report *and* justify inclusion and exclusion criteria, should report data on who was excluded at screening (although much exclusion happens before formal eligibility screening), and ideally should report how the trial population compares with the clinical population from which it was recruited. Age, co-morbidity and co-prescribing exclusions in particular require justification, not least because aging populations mean that for most conditions older people with multimorbidity and polypharmacy will be an increasing percentage of the clinically treated population [8, 36].

Assessment of the applicability of evidence should be explicitly reported by systematic reviews and in guideline development. Extrapolation of evidence is inevitable but should be explicitly justified when recommendations are made for *all* patients with a condition based on trial evidence from *narrow subsets* of the clinical population. Alternatively, guideline developers may consider making more nuanced or stratified recommendations that account for differences between trial and clinical populations [28, 37]. Guideline development therefore needs to be more informed by evidence about applicability by making greater use of epidemiological data describing how the clinical population differs from trial populations. This is also relevant for medicine regulation, where a better understanding of differences between trial and real-world populations may help in risk-minimization planning, including in the design of post-authorization safety studies.

Finally, although this review found a large volume of evidence about exclusion, the quality of that evidence was variable. Future studies in this field should clearly justify their selection of trials to examine and prioritize landmark trials or those cited in high-quality guidelines since these most clearly define standards of practice. The clinical population used to examine eligibility should be clearly described, and its appropriateness for measuring exclusion rates in the trial being examined justified. Studies of exclusion should report all eligibility criteria applied and all criteria not applied and discuss the implications of this for interpreting the findings.

## Conclusions

Most people with any of the physical conditions studied would be excluded from most trials of treatments for that condition. This is most commonly because the trial excludes older people and those with significant co-morbidity or co-prescribing. Population aging, increasing multimorbidity and increasing polypharmacy make it imperative that evidence of treatment effectiveness better match the people whom we actually treat in clinical practice.

## Supplementary information

**Additional file 1.** Search strategy and Supplementary Tables.

## Data Availability

Data sharing is not applicable to this article as no datasets were generated or analysed during the current study. All underlying studies and trials are documented and referenced in the supplementary file.

## Abbreviations

CI: Confidence interval
COPD: Chronic obstructive pulmonary disease
ESRD: End-stage renal disease
HIV: Human immunodeficiency virus
NNT: Number needed to treat
RA: Rheumatoid arthritis
RAIN: Rheumatoid Arthritis Investigators’ Network
RCT: Randomized controlled trial
VARA: Veterans’ Affairs Rheumatoid Arthritis
