# Supplementary material for: Exclusion rates in randomized controlled trials of treatments for physical conditions: a systematic review
Source: Trials. 2020 Feb 26;21:228. doi: 10.1186/s13063-020-4139-0 (PMC7045589; doi:10.1186/s13063-020-4139-0)
Supplement: Supplementary file 1 — Additional file 1. Search strategy and Supplementary Tables. [file 13063_2020_4139_MOESM1_ESM.docx]

# Exclusion rates in randomized-controlled trials of treatments for physical conditions: a systematic review

### Supplementary file

Jinzhang He BSc, Medical Student^1^ [j.z.he@dundee.ac.uk](mailto:j.z.he@dundee.ac.uk)

Daniel R. Morales PhD, Discovery Fellow^2^ [d.r.z.morales@dundee.ac.uk](mailto:d.r.z.morales@dundee.ac.uk)

Bruce Guthrie PhD, Professor of General Practice^3^ [bruce.guthrie@ed.ac.uk](mailto:bruce.guthrie@ed.ac.uk)

1. Ninewells Hospital and Medical School, University of Dundee, James Arrott Drive, Dundee DD2 1SY

2. Population Health and Genomics Division and Health Data Research UK, University of Dundee, Mackenzie Building, Kirsty Semple Way, Dundee DD2 4BF

3. Usher Institute Centre for Population Health Sciences, University of Edinburgh, Doorway 3 Old Medical School, Teviot Place, Edinburgh EH8 9AG

#### Corresponding author

Bruce Guthrie

[bruce.guthrie@ed.ac.uk](mailto:bruce.guthrie@ed.ac.uk)

+44 (0)131 650 9498

### Index of tables

[Supplementary Table S1: Clinical cohorts (42) used for comparison in the 37 studies included in the review 4](#_Toc7187258)

[Supplementary Table S2: Trial-cohort comparisons included in the review for trials in cardiovascular conditions: hypertension 13](#_Toc7187259)

[Supplementary Table S3: Trial-cohort comparisons included in the review for trials in cardiovascular conditions: heart failure 15](#_Toc7187260)

[Supplementary Table S4: Trial-cohort comparisons included in the review for trials in cardiovascular conditions: stroke and transient ischaemic attack 17](#_Toc7187261)

[Supplementary Table S5: Trial-cohort comparisons included in the review for trials in cardiovascular conditions: atrial fibrillation 19](#_Toc7187262)

[Supplementary Table S6: Trial-cohort comparisons included in the review for trials in cardiovascular conditions: coronary heart disease 20](#_Toc7187263)

[Supplementary Table S7: Trial-cohort comparisons included in the review for trials in cardiovascular conditions: lipid lowering for primary prevention 21](#_Toc7187264)

[Supplementary Table S8: Trial-cohort comparisons included in the review for trials in cardiovascular conditions: secondary prevention after myocardial infarction 22](#_Toc7187265)

[Supplementary Table S9: Trial-cohort comparisons included in the review for trials in diabetes mellitus 23](#_Toc7187266)

[Supplementary Table S10: Trial-cohort comparisons included in the review for trials in respiratory conditions: chronic obstructive pulmonary disease 25](#_Toc7187267)

[Supplementary Table S11: Trial-cohort comparisons included in the review for trials in respiratory conditions: asthma 28](#_Toc7187268)

[Supplementary Table S12: Trial-cohort comparisons included in the review for trials in respiratory conditions: bronchiectasis 30](#_Toc7187269)

[Supplementary Table S13: Trial-cohort comparisons included in the review for trials in cancer 31](#_Toc7187270)

[Supplementary Table S14: Trial-cohort comparisons included in the review for trials in rheumatoid arthritis 33](#_Toc7187271)

[Supplementary Table S15: Trial-cohort comparisons included in the review for trials in HIV 39](#_Toc7187272)

[Supplementary Table S16: Trial-cohort comparisons included in the review for trials in ‘other’ conditions 41](#_Toc7187273)

[Supplementary Table S17: Risk of bias by each of the three criteria 43](#_Toc7187274)

[Supplementary Table S18: Risk of bias assessment for each trial-clinical population pair 44](#_Toc7187275)

### Search Strategy

#### Review protocol

The systematic review protocol was registered with PROSPERO (Reference CRD42016042282).[^1^](#_ENREF_1) Relevant papers in MEDLINE and EMBASE were searched on 11^th^ February 2018, without restriction on publication date, using the following search strategies:

*MEDLINE Search*

1) Exclusion criteria (All Fields)

2) External validity (All Fields)

3) Applicability (All Fields)

4) Reproducibility of results (MeSH - E05.318.780.725)

5) Clinical trial as topic (MeSH – E05.318.760.250.500)

6) Randomized controlled trial as topic (MeSH – E05.318.760.250.500.365.500)

7) Generali* (Title/Abstract)

8) Bias epidemiology (MeSH – N05.715.350.150)

9) (1 OR 2 OR 3 OR 7) AND (4 OR 5 OR 6 OR 8)

*EMBASE Search*

1) Exclusion criteria (Title/Abstract)

2) External validity (Title/Abstract)

3) Applicability (Title Abstract)

4) Reproducibility of results (Title/Abstract)

5) Clinical trial as topic (Title/Abstract)

6) Randomized controlled trial as topic (Title/Abstract)

7) Generali* (Title/Abstract)

8) Bias epidemiology (Title/Abstract)

9) (1 OR 2 OR 3 OR 7) AND (4 OR 5 OR 6 OR 8)

Supplementary Table S1: Clinical cohorts (42) used for comparison in the 37 studies included in the review

| **Study** | **Condition** | **Cohort setting** | **Cohort description** | **Cohort size** | **No. of trials examined** | **Percentage of cohort excluded**  **Value if one trial, range and median if ≥2** |
| --- | --- | --- | --- | --- | --- | --- |
| **Aaltonen (2017)**[**^2^**](#_ENREF_2) | Rheumatoid arthritis | Primary care: National registry (National register for Biologic treatment in Finland) with additional data on patient comorbidities supplemented from Finnish Hospital Discharge Register and Finnish Cancer Register. | All patients with rheumatoid arthritis diagnosed and managed according to Finnish Current Care Guidelines on rheumatoid arthritis who had used tumour-necrosis factor inhibitors as first, second, or third biologic treatment. (2004 - 2014) | Range 44 – 1,021 (Varying due to specific requirements regarding prior biologics use) | 27 | Range 56.0 - 92.4  Median 80.0 |
| **Andersson (2016)**[**^3^**](#_ENREF_3) | Colorectal Cancer | Specialist: 24 hospitals in Denmark, Sweden and Uruguay that participated in COLOFOL pragmatic trial, retrospective review of clinical database. | All patients above 75 years of age who underwent radical surgery of Stage 2 or Stage 3 sporadic colorectal cancer, with a life expectancy of >2 years. (2005 - 2010) | 13,718 | 1 | 67.6 |
| **Bijker**  **(2002)**[**^4^**](#_ENREF_4) | Breast Cancer (Ductal Carcinoma In Situ) | Specialist: 5 centers in France, Italy, UK, and The Netherlands that participated in EORTC trial, clinical records collected to examine eligibility. | All patients presenting with DCIS and receiving treatment in these centers. (1986 - 1996) | 910 | 1 | 52.4 |
| **Bijkerk (2008)**[**^5^**](#_ENREF_5) | Irritable Bowel Syndrome | Primary care: 43 primary care centers in the Netherlands, clinical records of consecutive patients collected to examine eligibility. | All patients with definite IBS according to Rome II diagnostic criteria, and probable IBS pragmatically diagnosed by GP if experienced active IBS symptoms in past 4 weeks. (2008) | 1,288 | 1 | 73.1 |
| **Bress (2017)**[**^6^**](#_ENREF_6) | Hypertension | Primary care: Population based postal survey in USA (National Health and Nutrition Examination Survey) intended to be nationally representative and with oversampling of smaller important minority groups to ensure reliable data | Survey responders who are above 50 years of age with moderate to severe hypertension based on a mean of three blood pressure measurements during a single interview session or end organ damage (self-reported diabetes or cardiovascular disease, impaired renal function based on serum creatinine). (Pooled data from 1999-2000, 2000-2002, 2003-2004, 2005-2006) | 4,249 | 1 | 48.6 |
| **Carter cohort 1 (2009)**[**^7^**](#_ENREF_7) | Diabetic foot ulcers | Specialist: 18 outpatient wound-care centers across 17 states in USA with clinics in both urban and rural areas, retrospective review of electronic medical records of patient who gave consent. | All diabetic patients presenting to wound-care centre with Wagner grade 1 or higher foot ulcers. (Aug 2001 - May 2008) | 1,112 | 7 | Range 29.8 - 99.0  Median 93.3 |
| **Carter cohort 2 (2009)**[**^7^**](#_ENREF_7) | Venous ulcers | Specialist: 18 outpatient wound-care centers across 17 states in USA with clinics in both urban and rural areas, retrospective review of electronic medical records of patient who gave consent. | All non-diabetic patients presenting to wound-care centre with venous ulcers and clear evidence of venous stasis (Aug 2001 - May 2008). | 1,162 | 7 | Range 58.3 – 88.9  Median 83.6 |
| **Carter cohort 3 (2009)**[**^7^**](#_ENREF_7) | Pressure ulcers | Specialist: 18 outpatient wound-care centers across 17 states in USA with clinics in both urban and rural areas, retrospective review of electronic medical records of patient who gave consent. | All non-diabetic patients presenting to wound-care centre with National Pressure Ulcer Advisory Panel Stage 1 or higher foot ulcers. (Aug 2001 - May 2008) | 927 | 1 | 34.7 |
| **Chalmers (2016)**[**^8^**](#_ENREF_8) | Bronchiectasis | Specialist: 6 different observational cohort study databases from specialist centres in Scotland (Dundee, Edinburgh), England (Newcastle), Belgium (Leuven), Italy (Monza) and Ireland (Galway). | Dundee, Edinburgh, Monza, Newcastle: All patients ≥ 18 years of age with HRCT confirmed bronchiectasis. Patients with bronchiectasis secondary to interstitial lung disease, obstruction or tumours were not included. Patients on long term inhaled/oral antibiotics not included in Edinburgh cohort. Galway & Leuven: All patients with HRCT confirmed bronchiectasis. Patients with bronchiectasis secondary to interstitial lung disease, obstruction or tumours were not included in the Leuven cohort. (Dundee: 2012 - 2015, Edinburgh: 2008 - 2012, Galway: 2009 - 2014, Leuven: 2006 - 2012, Monza: 2011 - 2014, Newcastle: 2009 - 2013) | 1,672 | 10 | Range 49 - 93  Median 80 |
| **Costantino (2008)**[**^9^**](#_ENREF_9) | Heart failure | Specialist: Single heart failure outpatient clinic in Milan, Italy, clinical records of consecutive patients collected to examine eligibility. | Consecutive patients referred to heart failure outpatient clinic. (Jan 1993 - Dec 2003) | 299 | 16 | Range 29 - 92  Median 68 |
| **Dalela (2017)**[**^10^**](#_ENREF_10) | Prostate cancer | Specialist: Nationwide hospital-based cancer database (National Cancer Database) jointly supported by American college of Surgeons and American Cancer Society in USA. Research database collected to examine eligibility. | All men with histologically proven localised prostate cancer. (2004 - 2012) | 828,897 | 1 | 57.1 |
| **Desmaele (2016)**[**^11^**](#_ENREF_11) | Atrial fibrillation | Specialist: Research registry (Universitair Ziekenhuis Brussel Stroke Registry) from a single academic hospital in Brussels, Belgium, registry reviewed to examine eligibility | All patients admitted to Stroke unit with atrial fibrillation diagnosed either before admission with documented electrocardiogram findings or during admission with electrocardiogram indicative of atrial fibrillation. (1^st^ Jan 2010 - 30^th^ Jun 2014) | 468 | 3 | Range 52.4 - 60.7  Median 54.5 |
| **Fanning (2017)**[**^12^**](#_ENREF_12) | Atrial fibrillation | Specialist: Public hospital network serving a catchment of 750,000 people in Melbourne, Australia. Retrospective review of clinical data of consecutive attenders. | All patients presenting to hospital network and discharged with diagnosis of atrial fibrillation according to 10^th^ edition of International Classification of Diseases, Australian modification either as primary diagnosis or flagged up as comorbidity. (Jan 2012 - Dec 2015) | 4734 | 3 | Range 39.5 - 64.2  Median 47.4 |
| **Fischer (2012)**[**^13^**](#_ENREF_13) | Incisional Hernia | Specialist: 25 general and academic surgical centres in Germany, clinical records of consecutive patients collected to examine eligibility. | All patients with any diagnosis that required a midline laparotomy. (Jul 2004 - Sep 2006) | 629 | 1 | 62.5 |
| **Fortin (2006)**[**^14^**](#_ENREF_14) | Hypertension | Primary care: 21 family practices in the Saguenay region of Quebec, Canada, research database from previous prevalence study collected to examine eligibility. | All patients attending appointments. (Jan 2003 - July 2003) | 980 | 5 | Range 1.6 – 65.5  Median 48.5 |
| **Fossa**  **(2002)**[**^15^**](#_ENREF_15) | Bladder Cancer | Specialist: Single hospital in Norway, retrospective review of clinical records of referred patients. | All patients referred with muscle invasive T2-T4A, N0/NX, M0 bladder cancer. (Dec 1989 - May 1995) | 234 | 1 | 45.3 |
| **Gandhi (2005)**[**^16^**](#_ENREF_16) | HIV | Primary care: 23 sites in various care settings in the USA (including community support groups, drug rehabilitation programmes, primary care, general hospitals and academic hospitals), participating in the Women's Interagency HIV study, research database collected to examine eligibility. | All HIV seropositive women above age of 13 years who were enrolled into WIHS between 1994 and 1995. (2003 data extraction) | 1,717 | 31 | Range 0 – 67.6  Median 41.8 |
| **Hagg (2014)**[**^17^**](#_ENREF_17) | Atrial Fibrillation | Primary care: 9 private practitioners and 1 regional hospital in Skelleftea, Sweden, with access to records of all primary care centers in the region. Clinical records collected to examine eligibility. | All patients with diagnosed atrial fibrillation based on ICD-10 criteria and with risk factors indicating oral anti-coagulation. Patients not included into comparison cohort if a single isolated episode of atrial fibrillation or with reversible cause, high risk of bleeding, or currently on warfarin for any indication other than atrial fibrillation. (Dec 2010 data extraction) | 2,274 | 1 | 71.1 |
| **Halpin (2016)**[**^18^**](#_ENREF_18) | Chronic Obstructive Pulmonary Disease (COPD) | Primary care: General practices across UK that were part of Optimum Patient Care Research Database. Database contained information about secondary care, comorbidities and exacerbation history, and collected to examine eligibility. | All patients >40 years of age with confirmed diagnosis of COPD, and with data on FEV1, modified Medical Research Council score and full blood count. (Jan 2011 - Jan 2015) | 36,893 | 31 | Range 42.4 – 96.5  Median 77.1 |
| **Hansen (2016)**[**^19^**](#_ENREF_19) | Stroke (Intracerebral haemorrhage /haemorrhagic stroke) | Specialist: Research registry (Lund Stroke Register) involving multiple hospitals in Lund, Sweden, registry collected to examine eligibility. | All patients presenting with first-ever episode of haemorrhagic stroke or intracerebral haemorrhage | Range 220-271 (Varying due to missing data) | 11 | Range 58.6 - 98.4  Median 84.9 |
| **Janson (2009)**[**^20^**](#_ENREF_20) | Colon Cancer | Specialist: 8 hospitals in Sweden, clinical registry collected to examine eligibility. | All patients who underwent surgery for colon cancer based on ICD-10 criteria. (Mar 2003 data extraction) | 2,384 | 1 | 65.7 |
| **Jeremias (2008)**[**^21^**](#_ENREF_21) | Angina | Specialist: Single specialist center in Boston, USA, research registry of consecutive patients who underwent stenting procedure collected to examine eligibility. | Consecutive patients who underwent implantation of 1 or more Sirolimus-eluting stent. (Apr 2003 - Dec 2003) | 838 | 1 | 64.7 |
| **Jones (2017)**[**^22^**](#_ENREF_22) | Fibromyalgia | Primary care: Postal survey sent to patients registered with eight general practitioners in city of Aberdeen, Scotland and North Cheshire, England. P | Responders of postal survey with chronic pain consistent with the 1990 American College of Rheumatology classification criteria of fibromyalgia, pain score of ≥1 according to Chronic Pain Grade, consulted general practitioner regarding chronic pain in previous 12 months, access to land-line telephone, no other health condition requiring alternative medicine/treatments and no contraindications to exercise. | 1844 | 1 | 52.1 |
| **Jost**  **(2005)**[**^23^**](#_ENREF_23) | Heart Failure | Specialist: Single tertiary heart failure center in Germany, clinical registry collected to examine eligibility. | All patients presenting with chronic systolic heart failure (ejection fraction < 0.4) not caused by primary valvular/congenital heart disease, NYHA class II - IV on admission, survived initial hospital stay after treatment and on treatment with any combination of ACEi/ARB and diuretics. (Jan 1995 - Jun 2004) | 675 | 1 | 58.8 |
| **Klein**  **(1995)**[**^24^**](#_ENREF_24) | Type 1 Diabetes | Primary care: Primary practices participating in the Wisconsin Epidemiologic Study of Diabetic Retinopathy in the USA, research registry collected to examine eligibility. | All patients with clinically diagnosed type 1 diabetes mellitus before age of 30 years. (1980 - 1982) | 891 | 2 | Range 87.5 – 95.6  Median 91.6 |
| **Kruis**  **(2014)**[**^25^**](#_ENREF_25) | Chronic Obstructive Pulmonary Disease (COPD) | Primary care: Primary care database from the UNLOCK study, comprising of data from 7 primary care datasets from the Netherlands, United Kingdom, Sweden and Greece, collected to examine eligibility. | All patients with spirometry-validated COPD according to GOLD guidelines. UK dataset excluded patients with serious comorbidity affecting patient's ability to perform spirometry. Netherlands dataset excluded patients with terminal disease, immobility, substance abuse, history of asthma/allergic rhinitis before 40 years of age, dementia, oxygen therapy, and history of myocardial infarction within past 3 months. Greek dataset excluded patients with history of asthma, unstable cardiovascular disease, and any respiratory disease other than COPD. (Dates not stated) | 3,508 | 5 | Range 58 - 83  Median 77 |
| **Krumhols cohort 1 (2003)**[**^26^**](#_ENREF_26) | Acute Myocardial Infarction | Specialist: Non-governmental acute care hospitals in USA involved in the Cooperative Cardiovascular Project (CCP) registry, research registry collected to examine eligibility. | Medicare patients of age ≥65 years admitted with diagnosis of acute myocardial infarction. (Jun 1994 - Dec 1995) | 181,777 | 1 | 90.6 |
| **Krumhols cohort 2 (2003)**[**^26^**](#_ENREF_26) | Acute Myocardial Infarction | Specialist: 1658 hospitals in USA participating in the National Research Registry of Myocardial Infarction, research registry collected to examine eligibility. | Consecutive patients of age ≥65 years admitted with diagnosis of acute myocardial infarction. (June 1994 - Dec 1995) | 133,545 | 1 | 84.5 |
| **Lee**  **(2012)**[**^27^**](#_ENREF_27) | Atrial Fibrillation | Primary care: General Practice Research Database (GPRD), with clinical data from 630 general practices in UK and accounting for approximately 8% of UK population, collected to examine eligibility. | All patients of age ≥18 years with diagnosis of non-valvular atrial fibrillation who were alive on 31st March 2008. (31st March 2008 data extraction) | 83,898 | 3 | Range 36.2 – 52.5  Median 38.7 |
| **Lloyd-Jones (2001)**[**^28^**](#_ENREF_28) | Lipid lowering for primary prevention of coronary heart disease | Primary care: Previous cohort studies (Framingham Heart Study and Framingham Offsprings Study) in Framingham, USA, research registry collected to examine eligibility. | Subset of all participants age 30-74 years free of coronary heart disease at point of entry in both Framingham Heart Study and the Framingham Offsprings Study Survey. (Framingham Heart Study cohort: 1971 - 1974, Framingham Offsprings Study cohort: 1971 - 1975) | 5,368 | 4 | Range 69.7 – 89.1  Median 85.9 |
| **Maasland (2009)**[**^29^**](#_ENREF_29) | Acute stroke/TIA | Specialist: Research survey (National Stroke Survey) sent to 10 hospitals in the Netherlands, which are responsible of delivering care to approximately 10% of acute stroke patients. | All patients admitted or seen with acute stroke/TIA according to neurologist's assessment or if symptom onset <6 months ago. (Oct 2002 - May 2003) | 886 | 6 | Range 33.2 – 74.7  Median 51.8 |
| **Masoudi (2003)**[**^30^**](#_ENREF_30) | Heart Failure | Specialist: Various hospitals in Puerto Rico and Washington DC, USA participating in the National Heart Failure Project (NHF), research registry collected to examine eligibility. | Fee-for-service Medicare patients age ≥65 years with discharge diagnosis of heart failure, alive during index hospitalisation and with documented left ventricular systolic function. Patients who had chronic renal failure requiring haemodialysis or left against medical advice were not included into comparison cohort. (Apr 1998 - Mar 1999) | 20,388 | 3 | Range 74.7 – 86.6  Median 82.4 |
| **Markovic cohort 1 (2017)**[**^31^**](#_ENREF_31) | Brain injury | Specialist: Single academic hospital in Sweden, retrospective review of clinical data of consecutive attenders. | All patients, both inpatient and outpatient, with diagnosis of acute stroke or acute traumatic brain injury within past 4 months. (Sep 2011 - Nov 2014) | 626 | 1 | 45.0 |
| **Markovic cohort 2 (2017)**[**^31^**](#_ENREF_31) | Brain injury | Specialist: Single academic hospital in Sweden, retrospective review of clinical data of consecutive attenders. | All patients, both inpatient and outpatient, with diagnosis of acute stroke or acute traumatic brain injury within previous 4 - 12 months. (Sep 2011 - Nov 2014) | 234 | 1 | 35.9 |
| **Minnerup (2015)**[**^32^**](#_ENREF_32) | Stroke (Acute) | Specialist: 3 tertiary care stroke units in Switzerland, the UK and Germany, electronic healthcare record data collected to examine eligibility. | All patients admitted with acute stroke *or* transient ischaemic attack with continuing symptoms. (Apr-May 2014 in Switzerland, Dec 2013 to Jun 2014 in the UK, Dec 2007 to Dec 2009 in Germany). | 1,537 | 4 | Range 88.7 – 97.9  Median 96.2 |
| **Miro**  **(2015)**[**^33^**](#_ENREF_33) | Acute heart failure | Specialist: Representative Emergency Departments in Spain, clinical registry collected to examine eligibility. | All patients diagnosed with acute heart failure based on Framingham diagnostic criteria. (Three 1-month blocks: Apr 2007, May 2009, Nov-Dec 2011) | 5,845 | 1 | 82.5 |
| **Morin-Ben Abdullah (2016)**[**^34^**](#_ENREF_34) | Venous thrombo-embolism (prophylaxis) | Specialist: Single academic hospital in Montreal, Canada. Retrospective review of clinical data for selected attenders. | Randomly selected portion of 1664 patients presenting to an internal medicine teaching ward where thromboembolism prophylaxis was indicated (Padua score of ≥4) but not received any anticoagulant or antiplatelet therapy. (Jul 2013 - Jun 2014) | 118 | 9 | Range 23.7 - 78.8  Median 41.5 |
| **Patel (2017)**[**^35^**](#_ENREF_35) | Heart failure | Specialist: Single hospital in South London, UK with diagnostic heart failure clinic providing specialist heart failure services. Clinical database collected to examine eligibility. | All patients with echocardiography-proven heart failure with preserved ejection fraction. (Jan 2005 - Nov 2012) | 557 | 7 | Range 18.8 – 69.3  Median 51.5 |
| **Pedone (2003)**[**^36^**](#_ENREF_36) | Hypertension | Primary care: Nationally representative, population-based survey in USA (National Health and Nutrition Examination Survey III). | All patients age ≥17 with moderate to severe hypertension or any end organ damage as a result of hypertension. (1988 - 1974) | 3,828 | 16 | Range 67.2 – 98.8  Median 87.8 |
| **Neider cohort 1 (2017)**[**^37^**](#_ENREF_37) | Renal cancer | Specialist: Single hospital in Norway, retrospective review of clinical data of consecutive attenders. | All patients with renal cell carcinoma who have not received any previous therapy. (2006 - 2016) | 101 | 1 | 48.5 |
| **Neider cohort 2 (2017)**[**^37^**](#_ENREF_37) | Renal cancer | Specialist: Single hospital in Norway, retrospective review of clinical data of consecutive attenders. | Patients with renal cell carcinoma who have received one or two anti-angiogenic therapy regimens previously. (2006 - 2016) | 22 | 2 | Range 13.6 – 13.6  Median 13.6 |
| **Saunders cohort 1 (2013)**[**^38^**](#_ENREF_38) | Type 2 Diabetes | Primary care: National clinical registry (SCI-DC) comprising >99% of adults with diagnosed diabetes mellitus in Scotland, with clinical information from both primary and secondary care. | All patients with a clinical diagnosis of type 2 diabetes mellitus. (Extracted in 2008) | 180,590 | 5 | Range 64.3 – 96.5  Median 88.6 |
| **Saunders cohort 2 (2013)**[**^38^**](#_ENREF_38) | Type 2 Diabetes | Primary care: National clinical registry (SCI-DC) comprising >99% of adults with diagnosed diabetes mellitus in Scotland, with clinical information from both primary and secondary care. | All patients with newly diagnosed (<1 year) type 2 diabetes mellitus. (Extracted in 2008) | 13,687 | 2 | Range 49.3 – 68.2  Median 58.8 |
| **Schneider (1997)**[**^39^**](#_ENREF_39) | Alzheimer's Disease | Specialist: 9 specialist centers in USA under the ADDTC programme, research cohort collected to examine eligibility. | Consecutively enrolled patients with probable or possible Alzheimer's Disease based on the NINCDS-ADRDA criteria. (1985 - 1992) | 2,036 | 1 | 86.5 |
| **Sokka**  **(2003)**[**^40^**](#_ENREF_40) | Rheumatoid Arthritis | Specialist: Single academic rheumatology clinic in USA, retrospective review of clinical data of consecutive attenders. | Patients seen by a senior rheumatologist, met American College of Rheumatology criteria for rheumatoid arthritis. (Jan 1998 - Jun 2001) | 138 | 1 | 95.0 |
| **Spitzer (2016)**[**^41^**](#_ENREF_41) | Myocardial infarction | Specialist: Research registry in single academic hospital in Bern, Switzerland, retrospective review of clinical data of consecutive attenders. | All patients with diagnosis of ST-elevated myocardial infarction treated with percutaneous coronary intervention | 607 | 1 | 41.5 |
| **Terschuren (2010)**[**^42^**](#_ENREF_42) | Lymphoma (high grade non-Hodgkin) | Specialist: 20 regional hospitals and 2 university hospitals with outpatient cancer treatment facilities participating in the population-based North Germany Leukemia and Lymphoma Study, research database collected to examine eligibility. | Presumed patients with diagnosis of high grade non-Hodgkin's lymphoma. (1994 - 1998) | 328 | 1 | 70.4 |
| **Timmis (2016)**[**^43^**](#_ENREF_43) | Secondary prevention after myocardial infarction | Primary care: Research database (CALIBER) containing information sourced from primary and secondary care representative of whole population of England, research database collected to examine eligibility | All patients who survived at least a year after index admission to hospital with primary diagnosis of acute myocardial infarction or unstable angina with no subsequent re-admissions within a year. (Apr 2005 - Mar 2010) | 7,328 | 1 | 76.8 |
| **Travers (2007)**[**^44^**](#_ENREF_44) | Asthma | Primary care: Postal survey (Wellington Respiratory Survey) sent to randomly selected individuals from the electoral register in Wellington, New Zealand. | Responders to postal survey with reported diagnosis of asthma with increase in FEV1 ≥ 15% post-bronchodilator exposure, either symptoms of asthma or use of anti-asthmatic drugs in past 12 months, and with documented diurnal peak flow variation ≥20% in any of first 7 days of recording. (2002 - 2005) | 117 | 17 | Range 64 - 100  Median 96 |
| **Travers (2007)**[**^45^**](#_ENREF_45) | Chronic Obstructive Pulmonary Disease (COPD) | Primary care: Postal survey (Wellington Respiratory Survey) sent to randomly selected individuals from the electoral register in Wellington, New Zealand. | Responders to postal survey fulfilling spirometry-based COPD criteria (post-bronchodilator FEV1/FVC <0.7 with no specific pulmonary pathology), and completed pulmonary function test, skin prick test for common allergens, and a one-week peak flow diary. (2002 - 2005) | 179 | 17 | Range 8-100  Median 95 |
| **Treweek (2005)**[**^46^**](#_ENREF_46) | Breast Cancer | Primary care: Research database of all patients with incident breast cancer in Tayside, Scotland over a 16 year period, database collected to examine eligibility. | All patients with breast cancer. (Jan 1993 - Dec 2008) | 4,811 | 10 | Range 28.9 - 71.1  Median 56.6 |
| **Vardy (2009)**[**^47^**](#_ENREF_47) | Non-Small Cell Lung Cancer | Specialist: Single tertiary cancer centre in Sydney, Australia, research registry collected to examine eligibility. | All patients referred to tertiary cancer centre from Royal Prince Alfred Hospital and Concord Repatriation General Hospital with Stage IIIB and IV non-small cell lung cancer. (Oct 2001 - Dec 2002) | 185 | 3 | Range 65.4 – 71.9  Median 71.4 |
| **Vashisht cohort 1 (2016)**[**^48^**](#_ENREF_48) | Rheumatoid Arthritis | Specialist: 12 US sites participating in the Veterans Affair Rheumatoid Arthritis (VARA) registry, research registry collected to examine eligibility. | All patients with rheumatoid arthritis diagnosed by board-certified/board-eligible rheumatologist and satisfying the 1987 American College of Rheumatology classification criteria. (Jan 2003 - Sep 2010) | 1,523 | 30 | Range 72.7 – 99.1  Median 97.4 |
| **Vashisht cohort 2 (2016)**[**^48^**](#_ENREF_48) | Rheumatoid Arthritis | Primary care: 6 community practices and 1 academic site in USA as part of the Rheumatoid Arthritis Investigators Network (RAIN) database, research database collected to examine eligibility. | All patients with rheumatoid arthritis diagnosed by board-certified/board-eligible rheumatologist and satisfying the 1987 American College of Rheumatology classification criteria. (Mar 2002 - June 2011) | 1,548 | 30 | Range 64.9 – 93.5  Median 89.0 |
| **Ward**  **(1992)**[**^49^**](#_ENREF_49) | Stomach Cancer | Specialist: Hospitals in West Midlands, England participating in the West Midlands Cancer Registry, clinical registry collected to examine eligibility. | All patients aged 15 - 74 years with resected, histologically-proven primary carcinoma of the stomach. (1976 - 1980) | 1,207 | 1 | 41.3 |
| **Yeh**  **(2015)**[**^50^**](#_ENREF_50) | Angina | Specialist: Over 1200 hospitals part of the National Cardiovascular Data Registry in 50 states in USA, representing more than three-quarters of all PCI-performing hospitals, research registry collected to examine eligibility. | All patients who underwent PCI with a drug-eluting stent for angina/occluded coronary arteries and prescribed thienopyridine on discharge. (Sep 2009 - Jul 2011) | 794,278 | 1 | 5.8 |
| **Yoon**  **(2014)**[**^51^**](#_ENREF_51) | Atrial Fibrillation | Specialist: University Medical Center in South Korea, retrospective review of notes from consecutive attenders who gave consent. | All patients presenting with atrial fibrillation, on warfarin therapy with target INR 2.0 - 3.0, followed up for >6 months. (Oct 2011 - Oct 2013) | 695 | 4 | Range 32.3 – 41.2  Median 34.8 |

Supplementary Table S2: Trial-cohort comparisons included in the review for trials in cardiovascular conditions: hypertension

| **Study** | **Name of trial (date of publication)** | **Condition examined** | **Treatment** | **Source of original trial funding** | **Percentage (95% CI) excluded** |
| --- | --- | --- | --- | --- | --- |
| **Bress (2017)**[**^6^**](#_ENREF_6) | SPRINT (2015) | Hypertension | Standard treatment (systolic BP target <140mmHg) vs intensive-treatment group (systolic BP target <120mmHg) | Public | 48.6 (47.1 to 50.1) |
| **Fortin (2006)**[**^14^**](#_ENREF_14) | Appel (2003) | Hypertension | Established behavioral intervention vs Established behavioral intervention + DASH diet vs Advice only | Public | 65.5 (62.4 to 68.4) |
| **Fortin (2006)** | Hansson (1998) | Hypertension | Aspirin vs Placebo | Industry | 48.5 (43.2 to 53.9) |
| **Fortin (2006)** | Wing (2003) | Hypertension | Angiotensin-converting enzyme inhibitors vs Diuretics | Industry | 36.8 (30.1 to 43.9) |
| **Fortin (2006)** | ALLHAT (2002) | Hypertension | Chlorthalidone vs Amlodipine vs Lisinopril | Public | 1.6 (0.7 to 3.6) |
| **Fortin (2006)** | Sacks (2001) | Hypertension | Control diet vs DASH diet | Public | 61.1 (58.0 to 64.1) |
| **Pedone (2003)**[**^36^**](#_ENREF_36) | VA-NHBLI (1978) | Hypertension | Chlorthalidone 100mg + Reserpine 0.25mg vs Placebo | Public | 91.7 (90.8 to 92.5) |
| **Pedone (2003)** | HDPF (1979) | Hypertension | Systematic antihypertensive treatment program vs Referral to community medical therapy | Public | 71,2 (69.7 to 72.6) |
| **Pedone (2003)** | Oslo (1980) | Hypertension | Hydrochlorothiazide + Methyldopa/Propranolol vs No Treatment | Not reported | 97.1 (96.5 to 97.6) |
| **Pedone (2003)** | Australia (1980) | Hypertension | Chlorothiazide + Methyldopa/Propranolol/Pindolol + Hydralazine/Clonidine vs Placebo | Public | 82.9 (81.7 to 84.0) |
| **Pedone (2003)** | MRC (1985) | Hypertension | Bendroflumethiazide vs Placebo vs Propranolol vs Placebo | Industry | 79.0 (77.7 to 80.3) |
| **Pedone (2003)** | VA I (1967) | Hypertension | Hydrochlorothiazide + reserpine + hydralazine hydrochloride | Not reported | 96.0 (95.3 to 96.6) |
| **Pedone (2003)** | VA II (1970) | Hypertension | Active antihypertensive agents vs Placebo | Not reported | 81.1 (79.8 to 82.3) |
| **Pedone (2003)** | PHS (1977) | Hypertension | Diuretic + Rauwolfia serpentina vs Placebo | Public | 83.1 (81.9 to 84.3) |
| **Pedone (2003)** | HSCSG (1977) | Hypertension | Antihypertensive vs Placebo | Public | 98.8 (98.4 to 99.1) |
| **Pedone (2003)** | Barraclough (1971) | Hypertension | Bendroflumethiazide + Potassium supplement/Methyldopa/Debrisoquine vs Placebo | Industry | 89.0 (88.0 to 90.0) |
| **Pedone (2003)** | Carter (1970) | Hypertension | Antihypertensive Treatment (in the form of Thiazide diuretics + Methyldopa + Guanethidine/Bethanidine/Debrisoquine) vs No Treatment | Industry | 98,2 (97.7 to 98.6) |
| **Pedone (2003)** | EWPHE (1985) | Hypertension | Hydrochlorothiazide + Triamterene vs Placebo | Industry | 67.2 (65.7 to 68.7) |
| **Pedone (2003)** | Coope (1986) | Hypertension | Atenolol + Bendroflumethiazide + Methyldopa (in stepwise fashion) vs No Treatment | Industry | 88.9 (87.9 to 89.9) |
| **Pedone (2003)** | MRC-O (1992) | Hypertension | Amiloride/Hydrochlorothiazide vs Placebo vs Atenolol vs Placebo | Industry | 86.8 (85.1 to 87.2) |
| **Pedone (2003)** | SHEP  (1991) | Hypertension | Chlorthalidone + Atenolol vs Placebo | Public | 83.8 (82.6 to 84.9) |
| **Pedone (2003)** | STOP  (1991) | Hypertension | Antihypertensives (Atenolol/ Hydrochlorothiazide/ Amiloride/ Metoprolol/Pindolol) vs Placebo | Industry | 97.9 (97.4 to 98.3) |

Supplementary Table S3: Trial-cohort comparisons included in the review for trials in cardiovascular conditions: heart failure

| **Study** | **Name of trial (date of publication)** | **Condition examined** | **Treatment** | **Source of original trial funding** | **Percentage (95% CI) excluded** |
| --- | --- | --- | --- | --- | --- |
| **Costantino (2008)**[**^9^**](#_ENREF_9) | CIBIS (1994) | Heart failure | Bisoprolol vs Placebo | Not reported | 92 (88.3 to 94.5) |
| **Costantino (2008)** | GESICA (1994) | Heart failure | Amiodarone vs Standard Treatment | Not reported | 71 (65.5 to 75.8) |
| **Costantino (2008)** | Packer (1996) | Heart failure | Carvedilol vs Placebo | Industry | 62 (56.2 to 67.2) |
| **Costantino (2008)** | DIG (1997) | Heart failure | Digoxin vs Placebo | Industry | 29 (23.9 to 34.1) |
| **Costantino (2008)** | PRIME II (1997) | Heart failure | Ibopramine vs Placebo | Industry | 79 (74.0 to 83.2) |
| **Costantino (2008)** | FIRST (1997) | Heart failure | Intravenous Epoprostenol vs Standard Care | Not reported | 84 (79.4 to 87.7) |
| **Costantino (2008)** | Cohn (1998) | Heart failure | Vesnarinone 30mg vs Vesnarinone 60mg vs Placebo | Industry | 78 (72.9 to 82.3) |
| **Costantino (2008)** | RALES (1999) | Heart failure | Spironolactone vs Placebo | Industry | 76 (70.8 to 80.4) |
| **Costantino (2008)** | DIAMOND (1999) | Heart failure | Dofetilide vs Placebo | Industry | 52 (46.2 to 57.4) |
| **Costantino (2008)** | CIBIS II (1999) | Heart failure | Bisoprolol vs Placebo | Industry | 86 (81.6 to 89.4) |
| **Costantino (2008)** | MERIT-HF (2000) | Heart failure | Metoprolol vs Placebo | Industry | 48 (42.2 to 53.5) |
| **Costantino (2008)** | MACH-1 (2000) | Heart failure | Mibefradil vs Placebo | Industry | 65 (59.3 to 70.1) |
| **Costantino (2008)** | COPERNICUS (2001) | Heart failure | Carvedilol vs Placebo | Industry | 62 (56.2 to 67.2) |
| **Costantino (2008)** | Val-HeFT (2001) | Heart failure | Valsartan vs Placebo | Industry | 58 (52.2 to 63.3) |
| **Costantino (2008)** | BEST (2001) | Heart failure | Bucindolol vs Placebo | Public | 83 (79.4 to 87.7) |
| **Costantino (2008)** | CHARM (2003) | Heart failure | Candesartan vs Placebo | Industry | 35 (29.6 to 40.3) |
| **Jost (2005)**[**^23^**](#_ENREF_23) | MERIT-HF (1999) | Heart failure | Metoprolol vs Placebo | Industry | 58.8 (55.1 to 62.5) |
| **Masoudi (2003)**[**^30^**](#_ENREF_30) | SOLVD (1991) | Heart failure | Enalapril vs Placebo | Industry | 82.4 (81.9 to 83.0) |
| **Masoudi (2003)** | MERIT-HF (1999) | Heart failure | Metoprolol vs Placebo | Industry | 86.6 (86.2 to 87.1) |
| **Masoudi (2003)** | RALES (1999) | Heart failure | Spironolactone vs Placebo | Industry | 74.7 (74.1 to 75.3) |
| **Miro (2015)**[**^33^**](#_ENREF_33) | RELAX-AHF (2013) | Acute heart failure | Serelaxin vs Placebo | Industry | 17.5 (16.6 to 18.6) |
| **Patel (2017)**[**^35^**](#_ENREF_35) | DIG-Ancillary (1997) | Heart failure | Digoxin vs Placebo | Not reported | 24.8 (21.3 to 28.6) |
| **Patel (2017)** | CHARM-Preserved (2003) | Heart failure | Candesartan vs Placebo | Industry | 35.6 (31.6 to 39.7) |
| **Patel (2017)** | PEP-CHF (2006) | Heart failure | Perindopril vs Placebo | Industry | 66.6 (32.5 to 70.5) |
| **Patel (2017)** | I-PRESERVE (2008) | Heart failure | Irbesartan vs Placebo | Industry | 51.7 (47.5 to 55.9) |
| **Patel (2017)** | J-DHF (2013) | Heart failure | Carvedilol vs Placebo | Public | 18.8 (15.7 to 22.4) |
| **Patel (2017)** | TOPCAT (2014) | Heart failure | Spironolactone vs Placebo | Public | 51.5 (47.3 to 55.7) |
| **Patel (2017)** | PARAGON-HF (2018) | Heart failure | Valsartan + Sacubitril vs Valsartan only | Industry | 69.3 (65.3 to 75.1) |

Supplementary Table S4: Trial-cohort comparisons included in the review for trials in cardiovascular conditions: stroke and transient ischaemic attack

| **Study** | **Name of trial (date of publication)** | **Condition examined** | **Treatment** | **Source of original trial funding** | **Percentage (95% CI) excluded** |
| --- | --- | --- | --- | --- | --- |
| **Hansen (2016)**[**^19^**](#_ENREF_19) | STICH-I (2005) | Intracerebral Haemorrhage/  Haemorrhagic Stroke | Early surgery (haematoma evacuated within 24 hours) vs best medical treatment | Public | 64.5 (58.2 to 70.4) |
| **Hansen (2016)** | CHANT (2007) | Intracerebral Haemorrhage/  Haemorrhagic Stroke | NXY-059 (Disufenton sodium) vs Placebo | Industry | 83.6 (78.1 to 88.0) |
| **Hansen (2016)** | FAST (2008) | Intracerebral Haemorrhage/  Haemorrhagic Stroke | Placebo vs 20µg/kg recombinant factor VII vs 80µg/kg recombinant factor VII | Industry | 82.1 (76.4 to 86.7) |
| **Hansen (2016)** | STICH-II (2013) | Intracerebral Haemorrhage/  Haemorrhagic Stroke | Early surgery (within 12 hours) + medical treatment vs medical treatment alone (with later evacuation if judged necessary) | Public | 90.9 (86.5 to 94.0) |
| **Hansen (2016)** | INTERACT-2 (2013) | Intracerebral Haemorrhage/  Haemorrhagic Stroke | Intensive treatment (systolic BP <140mmHg within 1 hour) vs duideline-recommended treatment (systolic BP <180mmHg) | Public | 82.1 (76.4 to 86.7) |
| **Hansen (2016)** | CLEAR-III (2014) | Intracerebral Haemorrhage/  Haemorrhagic Stroke | Recombinant tissue plasminogen activator + external ventricular drain vs Placebo + external ventricular drain | Public | 96.4 (93.1 to 98.2) |
| **Hansen (2016)** | ATTACH-II (2016) | Intracerebral Haemorrhage/  Haemorrhagic Stroke | Intensive treatment (systolic blood pressure maintained between 110-139mmHg) vs Standard treatment (systolic blood pressure maintained between 140-179mmHg) | Public | 89.3 (84.6 to 92.8) |
| **Hansen (2016)** | MISTIE-III (2017) | Intracerebral Haemorrhage/  Haemorrhagic Stroke | Minimally invasive surgery + recombinant tissue plasminogen activator vs Standard medical treatment | Public | 94.5 (90.7 to 96.8) |
| **Hansen (2016)** | RESTART (2018) | Intracerebral Haemorrhage/  Haemorrhagic Stroke | Using antiplatelet drugs (one or more of aspirin, dipyridamole or clopidogrel) vs Avoiding antiplatelet drugs | Public | 84.9 (79.9 to 88.8) |
| **Hansen (2016)** | SWITCH (2018) | Intracerebral Haemorrhage/  Haemorrhagic Stroke | Decompressive craniectomy + best medical treatment vs Best medical treatment only | Public | 98.4 (95.7 to 99.5) |
| **Hansen (2016)** | TICH-2 (2018) | Intracerebral Haemorrhage/  Haemorrhagic Stroke | IV Tranexamic acid vs Placebo (normal saline 0.9%) | Public | 58.6 (51.8 to 65.2) |
| **Maasland (2009)**[**^29^**](#_ENREF_29) | ESPS-2 (1996) | Acute stroke/TIA | Dipyridamole vs Aspirin vs Dipyridamole + Aspirin vs Placebo | Not reported | 37.2 (34.1 to 40.5) |
| **Maasland (2009)** | CARPIE (1996) | Acute stroke/TIA | Aspirin vs Clopidogrel | Not reported | 67.7 (64.6 to 70.7) |
| **Maasland (2009)** | TACIP (2003) | Acute stroke/TIA | Triflusal vs Aspirin | Industry | 33.2 (30.2 to 36.6) |
| **Maasland (2009)** | MATCH (2004) | Acute stroke/TIA | Aspirin + Clopidogrel vs Placebo + Clopidogrel | Industry | 74.7 (71.8 to 77.5) |
| **Maasland (2009)** | ESPRIT (2006) | Acute stroke/TIA | Aspirin + Dipyridamole vs Aspirin | Public | 42.3 (39.1 to 45.6) |
| **Maasland (2009)** | PRoFESS (2008) | Acute stroke/TIA | Aspirin + Dipyridamole vs Clopidogrel + Telmisartan vs Placebo | Industry | 61.3 (58.0 to 64.4) |
| **Minnerup (2015)**[**^32^**](#_ENREF_32) | ALIAS 2 (2013) | Acute stroke | Intravenous Albumin (25%) vs Placebo | Industry | 95.7 (94.6 to 96.6) |
| **Minnerup (2015)** | AXIS 2 (2013) | Acute stroke | Filgrastim vs Placebo | Industry | 96.7 (95.7 to 97.5) |
| **Minnerup (2015)** | EuroHYP-1 (2014) | Acute stroke | Hypothermia + Best medical treatment vs Best medical treatment only | Public | 88.7 (87.0 to 90.2) |
| **Minnerup (2015)** | SWIFT PRIME (2015) | Acute stroke | Intravenous tissue plasmogen activator + endovascular thrombectomy vs Intravenous tissue plasmogen activator alone | Industry | 97.9 (97.1 to 98.5) |

Supplementary Table S5: Trial-cohort comparisons included in the review for trials in cardiovascular conditions: atrial fibrillation

| **Study** | **Name of trial (date of publication)** | **Condition examined** | **Treatment** | **Source of original trial funding** | **Percentage (95% CI) excluded** |
| --- | --- | --- | --- | --- | --- |
| **Desmaele (2016)**[**^11^**](#_ENREF_11) | RE-LY (2009) | Atrial fibrillation | Dabigatran 110mg vs Dabigatran 150mg vs Warfarin | Industry | 52.4 (47.7 to 56.9) |
| **Desmaele (2016)** | ARISTOTLE (2011) | Atrial fibrillation | Apixaban vs Warfarin vs Matching placebo respectively | Industry | 54.5 (49.8 to 59.0) |
| **Desmaele (2016)** | ROCKET-AF (2011) | Atrial fibrillation | Rivaroxaban vs Warfarin | Industry | 60.7 (56.1 to 65.1) |
| **Fanning (2017)**[**^12^**](#_ENREF_12) | RE-LY (2009) | Atrial fibrillation | Dabigatran 110mg vs Dabigatran 150mg vs Warfarin | Industry | 47.4 (46.0 to 48.9) |
| **Fanning (2017)** | ARISTOTLE (2011) | Atrial fibrillation | Apixaban vs Warfarin vs Matching placebo respectively | Industry | 39.5 (38.1 to 40.9) |
| **Fanning (2017)** | ROCKET-AF (2011) | Atrial fibrillation | Rivaroxaban vs Warfarin | Industry | 64.2 (62.8 to 65.6) |
| **Hagg (2014)**[**^17^**](#_ENREF_17) | ARISTOTLE (2011) | Atrial fibrillation | Apixaban vs Warfarin vs Matching placebo respectively | Industry | 71.1 (69.2 to 72.9) |
| **Lee (2012)**[**^27^**](#_ENREF_27) | RE-LY (2009) | Atrial fibrillation | Dabigatran 110mg vs Dabigatran 150mg vs Warfarin | Industry | 36.2 (35.8 to 36.5) |
| **Lee (2012)** | ARISTOTLE (2011) | Atrial fibrillation | Warfarin vs Apixaban vs Matching Placebos | Industry | 38.7 (38.4 to 39.0) |
| **Lee (2012)** | ROCKET-AF (2011) | Atrial fibrillation | Rivaroxaban vs Warfarin | Industry | 52.5 (52.1 to 52.8) |
| **Yoon (2014)**[**^51^**](#_ENREF_51) | RE-LY (2009) | Atrial fibrillation | Dabigatran 110mg vs Dabigatran 150mg vs Warfarin | Industry | 35.2 (31.6 to 39.0) |
| **Yoon (2014)** | ROCKET-AF (2011) | Atrial fibrillation | Rivaroxaban vs Warfarin | Industry | 34.5 (30.9 to 38.4) |
| **Yoon (2014)** | ARISTOTLE (2011) | Atrial fibrillation | Warfarin vs Apixaban | Industry | 32.3 (28.9 to 36.0) |
| **Yoon (2014)** | ENGAGE (2013) | Atrial fibrillation | Edoxaban vs Warfarin | Industry | 41.2 (37.4 to 45.1) |

Supplementary Table S6: Trial-cohort comparisons included in the review for trials in cardiovascular conditions: coronary heart disease

| **Study** | **Name of trial (date of publication)** | **Condition examined** | **Treatment** | **Source of original trial funding** | **Percentage (95% CI) excluded** |
| --- | --- | --- | --- | --- | --- |
| **Jeremias (2008)**[**^21^**](#_ENREF_21) | SIRIUS (2003) | Angina | Sirolimus-eluting Stent vs Standard Stent | Industry | 64.7 (61.4 to 67.8) |
| **Krumhols CCP (2003)**[**^26^**](#_ENREF_26) | GUSTO (1993) | Acute myocardial infarction | Streptokinase + Intravenous Heparin vs Streptokinase + Subcutaneous Heparin vs Accelerated tissue plasmogen activator + Intravenous Heparin vs Streptokinase + Accelerated tissue plasmogen activator + Intravenous Heparin | Industry | 90.6 (90.4 to 90.7) |
| **Krumhols NRRMI (2003)**[**^26^**](#_ENREF_26) | GUSTO (1993) | Acute myocardial infarction | Streptokinase + Intravenous Heparin vs Streptokinase + Subcutaneous Heparin vs Accelerated tissue plasmogen activator + Intravenous Heparin vs Streptokinase + Accelerated tissue plasmogen activator + Intravenous Heparin | Industry | 84.5 (84.3 to 84.7) |
| **Spitzer (2016)**[**^41^**](#_ENREF_41) | COMFORTABLE AM6(2012) | Acute myocardial infarction/  Unstable Angina | Biolimus-eluting stent vs Bare-metal stent | Industry | 41.5 (37.6 to 45.6) |
| **Yeh (2015)**[**^50^**](#_ENREF_50) | DAPT (2010) | Angina | Aspirin + Placebo (12 months) vs Aspirin + Thienopyridine (30 months) | Industry | 5.8 (5.7 to 5.8) |

Supplementary Table S7: Trial-cohort comparisons included in the review for trials in cardiovascular conditions: lipid lowering for primary prevention

| **Study** | **Name of trial (date of publication)** | **Condition examined** | **Treatment** | **Source of original trial funding** | **Percentage (95% CI) excluded** |
| --- | --- | --- | --- | --- | --- |
| **Lloyd-Jones (2001)**[**^28^**](#_ENREF_28) | LPC-CPPT (1984) | Lipid lowering for primary prevention | Cholestyramine vs Placebo | Public | 89.1 (88.3 to 89.9) |
| **Lloyd-Jones (2001)** | HHS (1987) | Lipid lowering for primary prevention | Gemfibrozil vs Placebo | Not reported | 84.8 (83.8 to 85.7) |
| **Lloyd-Jones (2001)** | WOSCOPS (1995) | Lipid lowering for primary prevention | Pravastatin vs Placebo | Industry | 87 (86.0 to 87.8) |
| **Lloyd-Jones (2001)** | AFCAPS/ TexCAPS (1998) | Lipid lowering for primary prevention | Lovastatin vs Placebo | Industry | 69.7 (68.4 to 70.9) |

Supplementary Table S8: Trial-cohort comparisons included in the review for trials in cardiovascular conditions: secondary prevention after myocardial infarction

| **Study** | **Name of trial (date of publication)** | **Condition examined** | **Treatment** | **Source of original trial funding** | **Percentage (95% CI) excluded** |
| --- | --- | --- | --- | --- | --- |
| **Timmis (2016)**[**^43^**](#_ENREF_43) | PEGASUS-TIMI-54 (2015) | Secondary prevention after myocardial infarction | Ticagrelor vs Placebo | Industry | 76.8 (75.8 to 77.8) |

Supplementary Table S9: Trial-cohort comparisons included in the review for trials in diabetes mellitus

| **Study** | **Name of trial (date of publication)** | **Condition examined** | **Treatment** | **Source of original trial funding** | **Percentage (95% CI) excluded** |
| --- | --- | --- | --- | --- | --- |
| **Saunders (2013)**[**^38^**](#_ENREF_38) | ACCORD (2008) | Type 2 diabetes | Intensive vs Standard Therapy | Public | 88.6 (88.4 to 88.7) |
| **Saunders (2013)** | ADVANCE (2008) | Type 2 diabetes | Intensive vs Standard Therapy | Industry | 64.3 (64.0 to 64.5) |
| **Saunders (2013)** | PROactive (2005) | Type 2 diabetes | Pioglitazone vs Placebo | Industry | 96.5 (96.4 to 96.6) |
| **Saunders (2013)** | RECORD (2009) | Type 2 diabetes | Rosiglitazone + Metformin vs Rosiglitazone + Sulfonylurea vs Metformin + Sulfonylurea | Industry | 90.8 (90.7 to 91.0) |
| **Saunders (2013)** | VADT (2009) | Type 2 diabetes | Intensive vs Standard Therapy | Public | 81.7 (81.5 to 81.9) |
| **Saunders (2013)** | UKDPS 33 (1998) | Type 2 diabetes | Sulfonylureas or Insulin vs Standard Therapy | Public | 49.3 (48.4 to 50.1) |
| **Saunders (2013)** | UKDPS 34 (1998) | Type 2 diabetes | Metformin vs Standard Therapy | Public | 68.2 (67.4 to 69.0) |
| **Carter (2009)**[**^7^**](#_ENREF_7) | Donahue 1998) | Diabetic foot ulcers | Collagen-Alginate dressing vs Gauze dressing | Not reported | 81.4 (79.0 to 83.6) |
| **Carter (2009)** | Armstrong (2005) | Diabetic foot ulcers | Negative wound pressure therapy vs Standard moist wound card | Industry | 47.9 (45.0 to 50.9) |
| **Carter (2009)** | Driver (2006) | Diabetic foot ulcers | Platelet-rich plasma gel vs Saline gel | Industry | 97.5 (96.4 to 98.3) |
| **Carter (2009)** | Marston 2003) | Diabetic foot ulcers | Fibroblast-derived bio-absorbable dermal mesh vs Standard wound dressing | Industry | 99.0 (98.2 to 99.4) |
| **Carter (2009)** | Kalani (2003) | Diabetic foot ulcers | Dalteparin vs Placebo | Industry | 96.0 (94.7 to 97.0) |
| **Carter (2009)** | Tsang (2003) | Diabetic foot ulcers | Actovegin 5% vs Actovegin 5% + Recombinant Human Epidermal Growth Factor 0.02% vs Actovegin 5% + Recombinant Human Epidermal Growth Factor 0.04% | Industry | 29.8 (27.2 to 32.5) |
| **Carter (2009)** | Veves (2002) | Diabetic foot ulcers | PROMOGRAN dressing vs Saline-moistened dressing | Industry | 93.3 (91.6 to 94.6) |
| **Klein (1995)**[**^24^**](#_ENREF_24) | DCCT (1993) | Type 1 diabetes | Conventional vs Intensive Treatment as Primary Prevention of Diabetic Retinopathy | Public | 95.6 (94.1 to 96.8) |
| **Klein (1995)** | DCCT (1993) | Type 1 diabetes | Conventional vs Intensive Treatment as Secondary intervention of Diabetic Retinopathy | Public | 87.5 (85.2 to 89.6) |

Supplementary Table S10: Trial-cohort comparisons included in the review for trials in respiratory conditions: chronic obstructive pulmonary disease

| **Study** | **Name of trial (date of publication)** | **Condition examined** | **Treatment** | **Source of original trial funding** | **Percentage (95% CI) excluded** |
| --- | --- | --- | --- | --- | --- |
| **Halpin (2016)**[**^18^**](#_ENREF_18) | NCT02172287 (2000) | COPD | Tiotropium + Placebo vs Salmeterol + Placebo | Industry | 84.3 (83.9 to 84.7) |
| **Halpin (2016)** | NCT00274014 (2003) | COPD | Tiotropium vs Placebo | Industry | 88.8 (88.5 to 89.1) |
| **Halpin (2016)** | NCT00274547 (2003) | COPD | Tiotropium vs Placebo | Industry | 74.9 (74.5 to 75.3) |
| **Halpin (2016)** | NCT00277264 (2004) | COPD | Tiotropium vs Placebo | Industry | 90.5 (90.2 to 90.8) |
| **Halpin (2016)** | UPLIFT (2008) | COPD | Tiotropium vs Placebo | Industry | 77.5 (77.1 to 77.9) |
| **Halpin (2016)** | Bateman (2010) | COPD | Tiotropium vs Placebo | Industry | 72.2 (71.7 to 72.7) |
| **Halpin (2016)** | POET-COPD (2011) | COPD | Tiotropium + Placebo vs Salmeterol + Placebo | Industry | 88.2 (87.9 to 88.5) |
| **Halpin (2016)** | TIOSPIR (2013) | COPD | Tiotropium 2.5μg vs Tiotropium 5μg vs Tiotropium 18mcg | Industry | 77.1 (76.7 to 77.5) |
| **Halpin (2016)** | Tie-COPD (2014) | COPD | Tiotropium vs Placebo | Industry | 75.0 (74.6 to 75.4 |
| **Halpin (2016)** | NCT00134979 (2008) | COPD | Formoterol + Tiotropium vs Formoterol vs Tiotropium vs Placebo | Industry | 73.7 (73.2 to 74.1) |
| **Halpin (2016)** | ACCLAIM/COPD I (2011) | COPD | Aclidinium vs Placebo | Industry | 58.5 (58.0 to 59.0) |
| **Halpin (2016)** | ATTAIN (2012) | COPD | Aclidinium 200μg vs Aclidinium 400μg vs Placebo | Industry | 55.4 (54.9 to 55.9) |
| **Halpin (2016)** | NCT01044459 (2011) | COPD | Aclidinium 200μg vs Aclidinium 400μg | Industry | 61.9 (61.4 to 62.4) |
| **Halpin (2016)** | Donohue (2010) | COPD | Indacaterol 150μg vs Indacaterol 300μg vs Placebo vs Tiotropium | Industry | 73.8 (73.3 to 74.2) |
| **Halpin (2016)** | Jones (2011) | COPD | Indacaterol vs Salmeterol vs Placebo | Industry | 65.1 (64.6 to 65.6) |
| **Halpin (2016)** | Yao (2014) | COPD | Indacaterol 150μg vs Indacaterol 300μg vs Placebo | Industry | 56.7 (56.2 to 57.2) |
| **Halpin (2016)** | INVIGORATE (2013) | COPD | Indacaterol vs Tiotropium | Industry | 93.5 (93.2 to 93.7) |
| **Halpin (2016)** | Ferguson (2014) | COPD | Olodaterol vs Placebo | Industry | 45.0 (44.5 to 45.5) |
| **Halpin (2016)** | Koch (2014) | COPD | Olodaterol 5μg vs Olodaterol 10μg vs Formoterol 15μg vs Placebo | Industry | 42.4 (41.9 to 42.9) |
| **Halpin (2016)** | GLOW2 (2014) | COPD | Glycopyrronium vs Tiotropium vs Placebo | Industry | 76.9 (76.5 to 77.3) |
| **Halpin (2016)** | GLOW1 (2014) | COPD | Glycopyrronium vs Placebo | Industry | 60.8 (60.3 to 61.3) |
| **Halpin (2016)** | GLOW7 (2013) | COPD | Glycopyrronium vs Placebo | Industry | 79.3 (78.9 to 79.7) |
| **Halpin (2016)** | SPARK (2013) | COPD | Indacaterol + Glycopyrronium vs Tiotropium vs Glycopyrronium | Industry | 96.5 (96.3 to 96.7) |
| **Halpin (2016)** | SHINE (2015) | COPD | Indacaterol + Glycopyrronium vs Indacaterol vs Glycopyrronium vs Tiotropium vs Placebo | Industry | 84.8 (84.4 to 85.2) |
| **Halpin (2016)** | ILLUMINATE (2013) | COPD | Indacaterol + Glycopyrronium vs Salmeterol + Fluticasone | Industry | 92.8 (92.5 to 93.1) |
| **Halpin (2016)** | LANTERN (2015) | COPD | Indacaterol + Glycopyrronium vs Salmeterol + Fluticasone | Industry | 91.4 (91.1 to 91.7) |
| **Halpin (2016)** | FLAME (2016) | COPD | Indacaterol + Glycopyrronium vs Salmeterol + Fluticasone | Industry | 96.1 (95.9 to 96.3) |
| **Halpin (2016)** | Donohue (2013) | COPD | Umeclidinium + Vilanterol vs Umeclidinium vs Vilanterol vs Placebo | Industry | 86.3 (85.9 to 86.6) |
| **Halpin (2016)** | Decramer (2014) | COPD | Umeclidinium 125μg + Vilanterol 25 μg vs Umeclidinium 62.5μg + Vilanterol 25 μg vs Tiotropium 18 μg vs Vilanterol 25 μg | Industry | 86.8 (86.5 to 87.1) |
| **Halpin (2016)** | NCT01777334 (2013) | COPD | Umeclidinium + Vilanterol vs Tiotropium | Industry | 85.3 (84.9 to 85.7) |
| **Halpin (2016)** | Buhl (2015) | COPD | Tiotropium-Olodaterol 2.5/5μg vs Tiotropium-Olodaterol 5/5μg vs Tiotropium 2.5 μg vs Tiotropium 5 μg vs Olodaterol 5 μg | Industry | 51.1 (50.6 to 51.6) |
| **Kruis (2014)**[**^25^**](#_ENREF_25) | ISOLDE (2000) | COPD | Fluticasone vs Placebo | Industry | 61 (59.3 to 62.6) |
| **Kruis (2014)** | TRISTAN (2003) | COPD | Salmeterol + Fluticasone vs Salmeterol vs Fluticasone vs Placebo | Industry | 83 (81.7 to 84.2) |
| **Kruis (2014)** | TORCH (2007) | COPD | Salmeterol + Fluticasone vs Salmeterol vs Fluticasone vs Placebo | Industry | 80 (78,.6 to 81.3) |
| **Kruis (2014)** | UPLIFT (2009) | COPD | Tiotropium vs Placebo | Industry | 58 (56.3 to 59.6) |
| **Kruis (2014)** | POET-COPD (2011) | COPD | Salmeterol vs Tiotropium | Industry | 77 (75.6 to 78.4) |
| **Travers (2007)**[**^45^**](#_ENREF_45) | Anthonisen (1994) | COPD | Smoking cessation + Ipratropium vs Smoking cessation + Placebo vs Usual care | Public | 95 (89.3 to 97.6) |
| **Travers (2007)** | COMVIBENT (1997) | COPD | Albuterol + Ipratropium vs Albuterol vs Ipratropium | Industry | 99 (95.3 to 99.8) |
| **Travers (2007)** | Boyd (1997) | COPD | Salmeterol 50μg twice daily vs Salmeterol 100μg twice daily vs Placebo | Industry | 94 (88.2 to 97.1) |
| **Travers (2007)** | Gross (1998) | COPD | Albuterol + Ipratropium vs Albuterol vs Ipratropium | Industry | 97 (91.5 to 98.7) |
| **Travers (2007)** | Mahler (1999) | COPD | Salmeterol vs Ipratropium vs Placebo | Industry | 91 (83.9 to 94.7) |
| **Travers (2007)** | Paulwels (1999) | COPD | Budesonide vs Placebo | Industry | 92 (85.5 to 96.2) |
| **Travers (2007)** | Lung Health Study Research Group (2000) | COPD | Triamcinolone vs Placebo | Industry | 90 (82.9 to 94.0) |
| **Travers (2007)** | Burge (2000) | COPD | Fluticasone vs Placebo | Industry | 80 (72.2 to 86.5) |
| **Travers (2007)** | Dahl (2001) | COPD | Formoterol vs Ipratropium vs Placebo | Industry | 84 (76.0 to 89.4) |
| **Travers (2007)** | Casaburi (2002) | COPD | Tiotropium vs Placebo | Industry | 95 (89.3 to 97.6) |
| **Travers (2007)** | Mahler (2002) | COPD | Salmeterol + Fluticasone vs Salmeterol vs Fluticasone vs Placebo | Industry | 97 (91.5 to 98.7) |
| **Travers (2007)** | Rossi (2002) | COPD | Formoterol vs Placebo + Theophylline vs Placebo | Industry | 84 (76.0 to 89.4) |
| **Travers (2007)** | Vincken (2002) | COPD | Tiotropium vs Ipratropium | Not reported | 96 (90.4 to 98.2) |
| **Travers (2007)** | Calverley (2003) | COPD | Salmeterol + fluticasone vs Salmeterol vs Fluticasone vs Placebo | Industry | 100 (96.8 to 100) |
| **Travers (2007)** | Calverley (2003) | COPD | Formoterol + Budesonide vs Formoterol vs Budesonide vs Placebo | Not reported | 98 (94.0 to 99.5) |
| **Travers (2007)** | Hanania (2003) | COPD | Salmeterol + Fluticasone vs Salmeterol vs Fluticasone vs Placebo | Industry | 97 (91.5 to 98.7) |
| **Travers (2007)** | Szafranski (2003) | COPD | Formoterol + Budesonide vs Formoterol vs Budesonide vs Placebo | Industry | 97 (91.5 to 98.7) |

Supplementary Table S11: Trial-cohort comparisons included in the review for trials in respiratory conditions: asthma

| **Study** | **Name of trial (date of publication)** | **Condition examined** | **Treatment** | **Source of original trial funding** | **Percentage (95% CI) excluded** |
| --- | --- | --- | --- | --- | --- |
| **Travers (2007)**[**^44^**](#_ENREF_44) | Greening (1994) | Asthma | Salmeterol 50μg twice daily + Beclometasone Dipropionate 200μg twice daily vs Beclometasone Dipropionate 500μg twice daily | Industry | 95 (90.7 to 97.3) |
| **Travers (2007)** | Woolcock (1996) | Asthma | Salmeterol 50μg twice daily + Beclometasone Dipropionate 500μg twice daily vs Salmeterol 100μg twice daily + Beclometasone Dipropionate 500μg twice daily vs Beclometasone Dipropionate 1000μg twice daily | Industry | 93 (88.0 to 95.7) |
| **Travers (2007)** | Pauwels (1994) | Asthma | Budesonide 100μg twice daily + Placebo vs Budesonide 100μg twice daily + Formoterol 12μg twice daily vs Budesonide 400μg twice daily + Placebo vs Budesonide 400μg twice daily + Formoterol 12μg twice daily | Industry | 94 (89.3 to 96.5) |
| **Travers (2007)** | Busse (1998) | Asthma | Budesonide 100μg twice daily vs Budesonide 200μg twice daily vs Budesonide 400μg twice daily vs Budesonide 800μg twice daily vs Placebo | Industry | 94 (89.3 to 96.5) |
| **Travers (2007)** | Reed (1998) | Asthma | Beclometasone Dipropionate vs Theophylline | Industry | 100 (98.8 to 100) |
| **Travers (2007)** | Wenzel (1998) | Asthma | Salmeterol vs Albuterol | Industry | 96 (92.1 to 98.1) |
| **Travers (2007)** | Laviolette (1999) | Asthma | Montelukast + Beclometasone Dipropionate vs Montelukast vs Beclometasone Dipropionate vs Placebo | Industry | 98 (94.4 to 99.1) |
| **Travers (2007)** | Bleecker (2000) | Asthma | Fluticasone vs Zafirlukast | Industry | 99 (96.0 to 99.7) |
| **Travers (2007)** | Nelson (2000) | Asthma | Salmeterol + Fluticasone vs Montelukast + Fluticasone | Industry | 93 (88.0 to 95.7) |
| **Travers (2007)** | Busse (2001) | Asthma | Omalizumab vs Placebo | Industry | 93 (86.6 to 94.9) |
| **Travers (2007)** | Fish (2001) | Asthma | Salmeterol vs Montelukast | Industry | 93 (88.0 to 95.7) |
| **Travers (2007)** | O'Byrne (2001) | Asthma | Group A: Budesonide 100μg twice daily + Formoterol 4.5μg twice daily vs Budesonide 100μg twice daily vs Placebo  Group B: Budesonide 100μg twice daily + Formoterol 4.5μg twice daily vs Budesonide 200μg twice daily vs Budesonide 200μg twice daily + Formoterol 4.5μg twice daily | Not reported | 64 (56.4 to 70.4) |
| **Travers (2007)** | Lalloo (2003) | Asthma | Formoterol 4.5μg twice daily + Budesonide 80μg twice daily vs Budesonide 200μg twice daily | Industry | 98 (94.4 to 99.1) |
| **Travers (2007)** | Pauwels (2003) | Asthma | Budesonide vs Placebo | Industry | 99 (96.0 to 99.7) |
| **Travers (2007)** | Price (2003) | Asthma | Montelukast + Budesonide 400μg twice daily vs Budesonide 800μg twice daily | Industry | 99 (96.0 to 99.7) |
| **Travers (2007)** | Ringdal (2003) | Asthma | Salmeterol + Fluticasone vs Montelukast + Fluticasone | Industry | 98 (94.4 to 99.1) |
| **Travers (2007)** | Vaquerizo (2003) | Asthma | Montilukast + Budesonide vs Budesonide | Industry | 97 (92.9 to 98.5) |

Supplementary Table S12: Trial-cohort comparisons included in the review for trials in respiratory conditions: bronchiectasis

| **Study** | **Name of trial (date of publication)** | **Condition examined** | **Treatment** | **Source of original trial funding** | **Percentage (95% CI) excluded** |
| --- | --- | --- | --- | --- | --- |
| **Chalmers (2016)**[**^8^**](#_ENREF_8) | EMBRACE (2012) | Bronchiectasis | Azithromycin vs Placebo | Public | 49 (46.6 to 51.4) |
| **Chalmers (2016)** | BLESS (2013) | Bronchiectasis | Erythromycin vs Placebo | Public | 71.7 (69.4 to 73.8) |
| **Chalmers (2016)** | BAT (2013) | Bronchiectasis | Azithromycin vs Placebo | Public | 81.1 (79.2 to 82.9) |
| **Chalmers (2016)** | AIR-BX (2014) | Bronchiectasis | Inhaled Aztreonam vs Placebo | Industry | 77 (74.9 to 78.9) |
| **Chalmers (2016)** | PROMIS (2014) | Bronchiectasis | Inhaled Colistin vs Placebo | Industry | 93 (87.3 to 90.3) |
| **Chalmers (2016)** | ORBIT-2 (2013) | Bronchiectasis | Inhaled Ciprofloxacin vs Placebo | Industry | 90 (88.4 to 91.3) |
| **Chalmers (2016)** | RESPIRE (2013) | Bronchiectasis | Ciprofloxacin dry powder vs Placebo | Industry | 85 (83.2 to 86.6) |
| **Chalmers (2016)** | GENT (2011) | Bronchiectasis | Nebulised Gentamicin vs Placebo | Industry | 79 (76.9 to 80.8) |
| **Chalmers (2016)** | DNAse (1998) | Bronchiectasis | Inhaled recombinant human DNase vs Placebo | Industry | 90 (88.4 to 91.3) |
| **Chalmers (2016)** | B301 (2013) | Bronchiectasis | Inhaled Mannitol vs Placebo | Industry | 49 (46.6 to 51.4) |

Supplementary Table S13: Trial-cohort comparisons included in the review for trials in cancer

| **Study** | **Name of trial (date of publication)** | **Condition examined** | **Treatment** | **Source of original trial funding** | **Percentage (95% CI) excluded** |
| --- | --- | --- | --- | --- | --- |
| **Andersson (2016)**[**^3^**](#_ENREF_3) | COLOFOL (2015) | Colorectal cancer | Frequent monitoring post-surgery (6, 12, 18, 24, 36 months) vs less frequent monitoring (12, 36 months) | Public | 67.6 (66.8 to 68.4) |
| **Bijker (2002)**[**^4^**](#_ENREF_4) | EORTC 10853 (2000) | Breast cancer (DCIS) | Local excision vs local excision + radiotherapy | Public | 52.4 (49.2 to 55.6) |
| **Dalela (2017)**[**^10^**](#_ENREF_10) | PIVOT (2012) | Prostate cancer (localised disease) | Radical Prostatectomy vs Observation | Public | 57.1 (57.0 to 57.2) |
| **Fossa (2002)**[**^15^**](#_ENREF_15) | International Collaboration (1999) | Bladder cancer | Cisplatin + Methotrexate + Vinblastine as neoadjuvant chemotherapy vs No neoadjuvant chemotherapy | Not reported | 45.3 (39.0 to 51.7) |
| **Janson (2009)**[**^20^**](#_ENREF_20) | COLOR (2002) | Colon cancer | Open Resection vs Laparoscopic Resection | Industry | 65.7 (63.8 to 67.6) |
| **Neider (2017)**[**^37^**](#_ENREF_37) | Motzer (2013) | Renal cancer | Pazopanib vs Sunitinib | Industry | 48.5 (38.5 to 58.6) |
| **Neider (2017)** | Choueiri (2015) | Renal cancer | Nivolumab vs Everolimus | Industry | 13.6 (3.6 to 36.0) |
| **Neider (2017)** | Motzer (2015) | Renal cancer | Cabozantinib vs Everolimus | Industry | 13.6 (3.6 to 36.0) |
| **Terschuren (2010)**[**^42^**](#_ENREF_42) | NHL-B1/NHL-B2 (2004) | Lymphoma | CHOP-14 vs CHOP-21 vs CHOP-14 + Etoposide vs CHOP-21 + Etoposide | Industry | 70.4 (65.3 to 75.1) |
| **Treweek (2005)**[**^46^**](#_ENREF_46) | ATAC (2002) | Breast cancer | Anastrozole vs Tamoxifen vs Anastrozole + Tamoxifen | Industry | 57.3 (55.9 to 58.7) |
| **Treweek (2005)** | BIG 1-98 (2005) | Breast cancer | Letrozole vs Tamoxifen | Industry | 55.5 (54.1 to 56.9) |
| **Treweek (2015)** | CANADIAN (2002) | Breast cancer | 42.5Gy whole breast irradiation vs 50Gy whole breast irradiation | Public | 55.0 (53.6 to 56.4) |
| **Treweek (2015)** | EORTC (2001) | Breast cancer | Standard Treatment vs Additional Radiation | Public | 56.6 (55.2 to 58.0) |
| **Treweek (2015)** | IES (2004) | Breast cancer | Tamoxifen vs Exemestane | Industry | 56.6 (55.2 to 58.0) |
| **Treweek (2015)** | MA17 (2003) | Breast cancer | Letrozole vs Placebo | Industry | 81.2 (80.1 to 82.3) |
| **Treweek (2015)** | NSABP B-28 (2005) | Breast cancer | Doxorubicin + Cyclophosphamide vs Doxorubicin + Cyclophosphamide + Palitaxel | Public | 80.4 (79.3 to 81.5) |
| **Treweek (2015)** | START A/ START B (2008) | Breast cancer | START A: 39 Gy vs 41.6Gy vs 50Gy Irradiation  START B: 40Gy vs 50Gy Irradiation | Public | 28.9 (27.7 to 30.2) |
| **Treweek (2015)** | TACT (2009) | Breast cancer | FEC + Docetaxel vs FEC or Epirubin + CMF | Industry | 77.2 (76.0 to 78.3) |
| **Treweek (2015)** | TARGIT-A (2010) | Breast cancer | Targeted intra-operative radiotherapy vs Conventional whole breast external beam radiotherapy | Public | 56.6 (55.2 to 58.0) |
| **Treweek (2015)** | TEAM (2011) | Breast cancer | Tamoxifen + Exemestane vs Exemestane | Industry | 54.6 (53.2 to 56.0) |
| **Vardy (2009)**[**^47^**](#_ENREF_47) | E1594 (2001) | Non-small cell lung cancer | Gemcitabine + Cisplatin vs Docetaxel + Cisplatin vs Paclitaxel + Carboplatin vs Paclitaxel + Cisplatin | Industry | 65.4 (58.3 to 71.9) |
| **Vardy (2009)** | SWOG 9509 (2001) | Non-small cell lung cancer | Vinorelbine + Cisplatin vs Paclitaxel + Carboplatin | Industry | 71.4 (64.5 to 77.4) |
| **Vardy (2009)** | TAS 326 (2003) | Non-small cell lung cancer | Docetaxel + Cisplatin vs Docetaxel + Carboplatin vs Vinorelbine + Cisplatin | Industry | 71.9 (65.0 to 77.9) |
| **Ward (1992)**[**^49^**](#_ENREF_49) | BSCG-1 (1989) | Stomach cancer | Surgery vs Radiotherapy vs Chemotherapy | Not reported | 41.3 (38.5 to 44.1) |

FEC: Fluorouracil, Epirubicin, Cyclophosphamide. CMF: Cyclophosphamide, Methotrexate, and Fluorouracil. CHOP-14: cyclophosphamide, doxorubicin, vincristine, prednisolone given every 2 weeks for 6 cycles. CHOP-21: cyclophosphamide, doxorubicin, vincristine, prednisolone given every 3 weeks for 6 cycles

Supplementary Table S14: Trial-cohort comparisons included in the review for trials in rheumatoid arthritis

| **Study*** | **Name of trial (date of publication)** | **Condition examined** | **Treatment** | **Source of original trial funding** | **Percentage (95% CI) excluded** |
| --- | --- | --- | --- | --- | --- |
| **Aaltonen (2017)**[**^2^**](#_ENREF_2) | Maini (1999) | Rheumatoid arthritis | Placebo vs Infliximab 3mg/kg every 4 weeks vs Infliximab 3mg/kg every 8 weeks vs Infliximab 10mg/kg every 4 weeks vs Infliximab 10mg/kg every 8 weeks | Industry | 81.0 (74.7 to 86.1) |
| **Aaltonen (2017)** | Moreland (1999) | Rheumatoid arthritis | Etanercept 10mg vs Etanercept 25mg vs Placebo | Industry | 80.0 (76.7 to 82.9) |
| **Aaltonen (2017)** | Weinblatt (1999) | Rheumatoid arthritis | Methotrexate vs Methotrexate + Etanercept | Industry | 63.0 (59.2 to 66.6) |
| **Aaltonen (2017)** | Lipsky (2000) | Rheumatoid arthritis | Methotrexate vs Infliximab 3mg/kg every 4 weeks + Methotrexate vs Infliximab 3mg/kg every 8 weeks + Methotrexate vs Infliximab 10mg/kg every 4 weeks + Methotrexate vs Infliximab 10mg/kg every 8 weeks + Methotrexate | Industry | 81.0 (74.7 to 86.1) |
| **Aaltonen (2017)** | ARMADA (2003) | Rheumatoid arthritis | Adalimumab 20mg vs Adalimumab 40mg vs Adalimumab 80mg vs Placebo | Industry | 64.0 (60.0 to 67.7) |
| **Aaltonen (2017)** | Keystone (2004) | Rheumatoid arthritis | Adalimumab 40mg vs Adalimumab 20mg vs Placebo | Industry | 56.0 (52.1 to 59.9) |
| **Aaltonen (2017)** | Van de Putte (2004) | Rheumatoid arthritis | Adalimumab 20mg weekly vs Adalimumab 20mg every other week vs Adalimumab 40mg weekly vs Adalimumab 40mg every other week vs Placebo | Industry | 80.0 (77.1 to 82.6) |
| **Aaltonen (2017)** | Combe (2006) | Rheumatoid arthritis | Etanercept vs Sulfasalazine vs Etanercept + Sulfasalazine | Industry | 84.0 (81.0 to 86.7) |
| **Aaltonen (2017)** | TEMPO (2006) | Rheumatoid arthritis | Methotrexate vs Etanercept vs Methotrexate + Etanercept | Industry | 86.0 (83.7 to 88.0) |
| **Aaltonen (2017)** | Westhovens (2006) | Rheumatoid arthritis | Placebo vs Infliximab 3mg/kg vs Infliximab 10mg/kg | Industry | 60.0 (52.8 to 66.8) |
| **Aaltonen (2017)** | Kim (2007) | Rheumatoid arthritis | Adalimumab 40mg vs Adalimumab 20mg vs Placebo + Methotrexate | Industry | 68.0 (65.0 to 70.9) |
| **Aaltonen (2017)** | ATTEST (2008) | Rheumatoid arthritis | Placebo vs infliximab 3mg/kg vs Abatacept 10mg/kg | Industry | 83.0 (76.8 to 87.8) |
| **Aaltonen (2017)** | Keystone (2008) | Rheumatoid arthritis | Certolizumab pegol 200mg vs Certolizumab pegol 400mg vs Placebo | Industry | 92.4 (85.1 to 96.4) |
| **Aaltonen (2017)** | FAST4WARD (2009) | Rheumatoid arthritis | Certolizumab pegol vs Placebo | Industry | 89.0 (79.7 to 94.5) |
| **Aaltonen (2017)** | GO-AFTER (2009) | Rheumatoid arthritis | Golimumab 50mg vs Golimumab 100mg vs Placebo | Industry | 82.0 (71.4 to 95.0) |
| **Aaltonen (2017)** | RAPID 2 (2009) | Rheumatoid arthritis | Certolizumab pegol 200mg vs Certolizumab pegol 400mg vs Placebo | Industry | 90.5 (81.6 to 95.6) |
| **Aaltonen (2017)** | GO-FORWARD (2010) | Rheumatoid arthritis | Methotrexate vs Golimumab 50mg + Methotrexate vs Golimumab 100mg + Methotrexate vs Golimumab 100mg | Industry | 63.0 (51.5 to 73.2) |
| **Aaltonen (2017)** | Choy (2012) | Rheumatoid arthritis | Certolizumab pegol vs Placebo | Industry | 90.0 (81.4 to 95.0) |
| **Aaltonen (2017)** | GO-FORTH (2012) | Rheumatoid arthritis | Methotrexate vs Golimumab 50mg + Methotrexate vs Golimumab 100mg + Methotrexate | Industry | 69.0 (60.2 to 76.5) |
| **Aaltonen (2017)** | REALISTIC (2012) | Rheumatoid arthritis | Certolizumab pegol vs Placebo | Industry | 64.0 (47.7 to 77.2) |
| **Aaltonen (2017)** | TEAR (2012) | Rheumatoid arthritis | Immediate Methotrexate + Etanercept vs Immediate Methotrexate + Sulfasalazine + Hydroxychloroquine vs Step-up to Methotrexate + Etanercept vs Step-up to Methotrexate + Sulfasalazine + Hydroxychloroquine | Industry | 82.0 (78.8 to 84.8) |
| **Aaltonen (2017)** | ADACTA (2013) | Rheumatoid arthritis | Tocilizumab vs Adalimumab | Industry | 74.0 (70.4 to 77.3) |
| **Aaltonen (2017)** | AMPLE (2013) | Rheumatoid arthritis | Abatacept vs Adalimumab | Industry | 81.0 (77.7 to 83.9) |
| **Aaltonen (2017)** | O’Dell (2013) | Rheumatoid arthritis | Methotrexate + Etanercept vs Methotrexate + Sulfasalazine + Hydroxychloroquine | Public | 58.0 (54.5 to 61.4) |
| **Aaltonen (2017)** | Yoo (2013) | Rheumatoid arthritis | Infliximab vs CT-P13 | Industry | 60.0 (52.8 to 66.8) |
| **Sokka (2003)**[**^40^**](#_ENREF_40) | ATTRACT (2002) | Rheumatoid arthritis | Infliximab 3mg/Kg (for 4 or 8 weeks) + Methotrexate vs Infliximab 10mg/kg (for 4 or 8 weeks) + Methotrexate vs Placebo + Methotrexate | Not reported | 95 (89.9 to 97.5) |
| **Vashisht VARA (2016)**[**^48^**](#_ENREF_48) | Moreland (1999) | Rheumatoid arthritis | Etanercept 10mg vs Etanercept 25mg vs Placebo | Industry | 97.7 (96.7 to 98.3) |
| **Vashisht RAIN (2016)**[**^48^**](#_ENREF_48) | Moreland (1999) | Rheumatoid arthritis | Etanercept 10mg vs Etanercept 25mg vs Placebo | Industry | 92.9 (91.5 to 94.1) |
| **Vashisht VARA (2016)** | Maini  (1999) | Rheumatoid arthritis | Infliximab (3mg/kg for 4 weeks vs 3mg/kg for 8weeks vs 10mg/kg for 4weeks vs 10/mg/kg for 8weeks) vs Placebo | Industry | 98.4 (97.6 to 98.9) |
| **Vashisht RAIN (2016)** | Maini  (1999) | Rheumatoid arthritis | Infliximab (3mg/kg for 4 weeks vs 3mg/kg for 8weeks vs 10mg/kg for 4weeks vs 10/mg/kg for 8weeks) vs Placebo | Industry | 91.6 (90.1 to 92.9) |
| **Vashisht VARA (2016)** | St Clair (2004) | Rheumatoid arthritis | Infliximab 3mg/kg vs Infliximab 6mg/kg vs Placebo | Industry | 99.1 (98.5 to 99.5) |
| **Vashisht RAIN (2016)** | St Clair (2004) | Rheumatoid arthritis | Infliximab 3mg/kg vs Infliximab 6mg/kg vs Placebo | Industry | 93.5 (92.1 to 94.6) |
| **Vashisht VARA (2016)** | Weinblatt (2003) | Rheumatoid arthritis | Adalimumab (20mg vs 40mg vs 80mg) + Background Methotrexate vs Placebo + Background Methotrexate | Industry | 75.6 (73.4 to 77.7) |
| **Vashisht RAIN (2016)** | Weinblatt (2003) | Rheumatoid arthritis | Adalimumab (20mg vs 40mg vs 80mg) + Background Methotrexate vs Placebo + Background Methotrexate | Industry | 74.7 (725 to 76.8) |
| **Vashisht VARA (2016)** | Furst (2003) | Rheumatoid arthritis | Adalimumab 40mg + Continuing standard anti-rheumatics vs Placebo + Continuing standard anti-rheumatics | Industry | 79.5 (77.3 to 81.4) |
| **Vashisht RAIN (2016)** | Furst (2003) | Rheumatoid arthritis | Adalimumab 40mg + Continuing standard anti-rheumatics vs Placebo + Continuing standard anti-rheumatics | Industry | 7.0 (73.8 to 78.0) |
| **Vashisht VARA (2016)** | Keystone (2004) | Rheumatoid arthritis | Adalimumab 20mg vs Adalimumab 40mg vs Placebo | Industry | 94.2 (92.9 to 95.2) |
| **Vashisht RAIN (2016)** | Keystone (2004) | Rheumatoid arthritis | Adalimumab 20mg vs Adalimumab 40mg vs Placebo | Industry | 84.4 (82.5 to 86.1) |
| **Vashisht VARA (2016)** | Van de Putte (2004) | Rheumatoid arthritis | Adalimumab (20mg fortnightly with placebo on alternate weeks vs 20mg weekly vs 40mg weekly vs 40mg fortnightly with placebo on alternate weeks) vs Placebo | Industry | 97.4 (96.4 to 98.1) |
| **Vashisht RAIN (2016)** | Van de Putte (2004) | Rheumatoid arthritis | Adalimumab (20mg fortnightly with placebo on alternate weeks vs 20mg weekly vs 40mg weekly vs 40mg fortnightly with placebo on alternate weeks) vs Placebo | Industry | 91.6 (90.1 to 92.9) |
| **Vashisht VARA (2016)** | Keystone (2008) | Rheumatoid arthritis | Certolizumab 400mg + Methotrexate vs Certolizumab 200mg + Methotrexate vs Placebo + Methotrexate | Industry | 94.8 (93.5 to 95.8) |
| **Vashisht RAIN (2016)** | Keystone (2008) | Rheumatoid arthritis | Certolizumab 400mg + Methotrexate vs Certolizumab 200mg + Methotrexate vs Placebo + Methotrexate | Industry | 87.0 (85.2 to 88.5) |
| **Vashisht VARA (2016)** | Smolen (2009) | Rheumatoid arthritis | Certolizumab pegol 200mg + Methotrexate vs Certolizumab pegol 400mg + Methotrexate vs Placebo + Methotrexate | Industry | 87.7 (85.9 to 89.2) |
| **Vashisht RAIN (2016)** | Smolen (2009) | Rheumatoid arthritis | Certolizumab pegol 200mg + Methotrexate vs Certolizumab pegol 400mg + Methotrexate vs Placebo + Methotrexate | Industry | 79.9 (77.8 to 81.8) |
| **Vashisht VARA (2016)** | Fleischmann (2009) | Rheumatoid arthritis | Certolizumab pegol 400mg vs Placebo | Industry | 98.7 (98.0 to 99.1) |
| **Vashisht RAIN (2016)** | Fleischmann (2009) | Rheumatoid arthritis | Certolizumab pegol 400mg vs Placebo | Industry | 91.6 (90.1 to 92.9) |
| **Vashisht VARA (2016)** | Keystone (2009) | Rheumatoid arthritis | Golimumab 100mg + Methotrexate vs Golimumab 50mg + Methotrexate vs Golimumab 100mg + Methotrexate vs Placebo + Methotrexate | Industry | 98.1 (97.3 to 98.7) |
| **Vashisht RAIN (2016)** | Keystone (2009) | Rheumatoid arthritis | Golimumab 100mg + Methotrexate vs Golimumab 50mg + Methotrexate vs Golimumab 100mg + Methotrexate vs Placebo + Methotrexate | Industry | 85.1 (83.2 to 86.8) |
| **Vashisht VARA (2016)** | Emery (2009) | Rheumatoid arthritis | Golimumab 100mg + Methotrexate vs Golimumab 50mg + Methotrexate vs Golimumab 100mg + Placebo vs Placebo + Methotrexate | Industry | 95.5 (94.3 to 96.4) |
| **Vashisht RAIN (2016)** | Emery (2009) | Rheumatoid arthritis | Golimumab 100mg + Methotrexate vs Golimumab 50mg + Methotrexate vs Golimumab 100mg + Placebo vs Placebo + Methotrexate | Industry | 82.5 (80.5 to 84.3) |
| **Vashisht VARA (2016)** | Smolen (2012) | Rheumatoid arthritis | Golimumab 100mg vs Golimumab 50mg vs Placebo | Industry | 72.7 (70.4 to 74.9) |
| **Vashisht RAIN (2016)** | Smolen (2012) | Rheumatoid arthritis | Golimumab 100mg vs Golimumab 50mg vs Placebo | Industry | 64.9 (62.4 to 67.2) |
| **Vashisht VARA (2016)** | Bresnihan (1998) | Rheumatoid arthritis | Interleukin-1 receptor antagonist (30mg vs 75mg vs 150mg) vs Placebo | Not reported | 98.7 (98.0 to 99.1) |
| **Vashisht RAIN (2016)** | Bresnihan (1998) | Rheumatoid arthritis | Interleukin-1 receptor antagonist (30mg vs 75mg vs 150mg) vs Placebo | Not reported | 92.9 (91.5 to 94.1) |
| **Vashisht VARA (2016)** | Cohen (2002) | Rheumatoid arthritis | Anakinra (0.04mg/kg vs 0.1mg/kg vs 0.4mg/kg vs 1mg/kg vs 2mg/kg) vs Placebo | Industry | 98.7 (98.0 to 99.1) |
| **Vashisht RAIN (2016)** | Cohen (2002) | Rheumatoid arthritis | Anakinra (0.04mg/kg vs 0.1mg/kg vs 0.4mg/kg vs 1mg/kg vs 2mg/kg) vs Placebo | Industry | 89.0 (87.3 to 90.4) |
| **Vashisht VARA (2016)** | Fleischmann (2003) | Rheumatoid arthritis | Anakinra vs Placebo | Industry | 97.4 (96.4 to 98.1) |
| **Vashisht RAIN (2016)** | Fleischmann (2003) | Rheumatoid arthritis | Anakinra vs Placebo | Industry | 20.1 (18.2 to 22.2) |
| **Vashisht VARA (2016)** | Cohen (2004) | Rheumatoid arthritis | Anakinra 100mg + Methotrexate vs Placebo + Methotrexate | Industry | 98.7 (98.0 to 99.1) |
| **Vashisht RAIN (2016)** | Cohen (2004) | Rheumatoid arthritis | Anakinra 100mg + Methotrexate vs Placebo + Methotrexate | Industry | 89.0 (87.3 to 90.4) |
| **Vashisht VARA (2016)** | Edwards (2004) | Rheumatoid arthritis | Methotrexate vs Rituximab vs Rituximab + Cyclophosphamide vs Rituximab + Methotrexate | Industry | 97.4 (96.4 to 98.1) |
| **Vashisht RAIN (2016)** | Edwards (2004) | Rheumatoid arthritis | Methotrexate vs Rituximab vs Rituximab + Cyclophosphamide vs Rituximab + Methotrexate | Industry | 90.3 (88.7 to 91.7) |
| **Vashisht VARA (2016)** | Cohen (2006) | Rheumatoid arthritis | Rituximab + Methotrexate vs Placebo + Methotrexate | Industry | 98.1 (97.3 to 98.7) |
| **Vashisht RAIN (2016)** | Cohen (2006) | Rheumatoid arthritis | Rituximab + Methotrexate vs Placebo + Methotrexate | Industry | 91.6 (90.1 to 92.9) |
| **Vashisht VARA (2016)** | Genovese (2005) | Rheumatoid arthritis | Abatacept + DMARDs vs Placebo + DMARDs | Industry | 86.4 (84.5 to 88.0) |
| **Vashisht RAIN (2016)** | Genovese (2005) | Rheumatoid arthritis | Abatacept + DMARDs vs Placebo + DMARDs | Industry | 85.5 (83.6 to 87.1) |
| **Vashisht VARA (2016)** | Kremer (2006) | Rheumatoid arthritis | Abatacept 10mg/kg vs Placebo | Industry | 95.5 (94.3 to 96.4) |
| **Vashisht RAIN (2016)** | Kremer (2006) | Rheumatoid arthritis | Abatacept 10mg/kg vs Placebo | Industry | 89.6 (88.0 to 91.0) |
| **Vashisht VARA (2016)** | Westhovens (2009) | Rheumatoid arthritis | Abatacept 10mg/kg + Methotrexate vs Placebo + Methotrexate | Industry | 98.1 (97.3 to 98.7) |
| **Vashisht RAIN (2016)** | Westhovens (2009) | Rheumatoid arthritis | Abatacept 10mg/kg + Methotrexate vs Placebo + Methotrexate | Industry | 90.9 (88.8 to 91.7) |
| **Vashisht VARA (2016)** | Genovese (2008) | Rheumatoid arthritis | Tocilizumab 8mg/kg + DMARDs vs Placebo + DMARDS | Industry | 97.4 (96.4 to 98.1) |
| **Vashisht RAIN (2016)** | Genovese (2008) | Rheumatoid arthritis | Tocilizumab 8mg/kg + DMARDs vs Placebo + DMARDS | Industry | 89.0 (87.3 to 90.4) |
| **Vashisht VARA (2016)** | Emery (2008) | Rheumatoid arthritis | Tocilizumab 9mg/kg vs Placebo | Industry | 96.8 (95.8 to 97.6) |
| **Vashisht RAIN (2016)** | Emery (2008) | Rheumatoid arthritis | Tocilizumab 9mg/kg vs Placebo | Industry | 86.4 (84.5 to 88.0) |
| **Vashisht VARA (2016)** | Smolen (2008) | Rheumatoid arthritis | Tocilizumab 8mg/kg + Methotrexate vs Tocilizumab 4mg/kg + Methotrexate vs Placebo + Methotrexate | Industry | 97.4 (96.4 to 98.1) |
| **Vashisht RAIN (2016)** | Smolen (2008) | Rheumatoid arthritis | Tocilizumab 8mg/kg + Methotrexate vs Tocilizumab 4mg/kg + Methotrexate vs Placebo + Methotrexate | Industry | 88.3 (86.5 to 89.8) |
| **Vashisht VARA (2016)** | Jones (2010) | Rheumatoid arthritis | Methotrexate 24 weeks vs Tocilizumab 8mg/kg 24 weeks vs Placebo 8 weeks + Tocilizumab 8mg/kg 16 weeks | Industry | 94.8 (93.5 to 95.8) |
| **Vashisht RAIN (2016)** | Jones (2010) | Rheumatoid arthritis | Methotrexate 24 weeks vs Tocilizumab 8mg/kg 24 weeks vs Placebo 8 weeks + Tocilizumab 8mg/kg 16 weeks | Industry | 87.0 (85.2 to 88.5) |
| **Vashisht VARA (2016)** | Kremer (2011) | Rheumatoid arthritis | Tocilizumab 8mg/kg + Stable Methotrexate vs Tocilizumab 4mg/kg + Stable Methotrexate vs Placebo + Stable Methotrexate | Industry | 97.4 (96.4 to 98.1) |
| **Vashisht RAIN (2016)** | Kremer (2011) | Rheumatoid arthritis | Tocilizumab 8mg/kg + Stable Methotrexate vs Tocilizumab 4mg/kg + Stable Methotrexate vs Placebo + Stable Methotrexate | Industry | 88.3 (86.5 to 89.8) |
| **Vashisht VARA (2016)** | Burmester (2013) | Rheumatoid arthritis | Tofacitinib 5mg + Methotrexate vs Tofacitinib 10mg + Methotrexate vs Placebo + Methotrexate (Placebo group then advanced to either Tofacitinib 5mg or 10mg after 3rd month) | Industry | 97.4 (96.4 to 98.1) |
| **Vashisht RAIN (2016)** | Burmester (2013) | Rheumatoid arthritis | Tofacitinib 5mg + Methotrexate vs Tofacitinib 10mg + Methotrexate vs Placebo + Methotrexate (Placebo group then advanced to either Tofacitinib 5mg or 10mg after 3rd month) | Industry | 89.0 (87.3 to 90.4) |
| **Vashisht VARA (2016)** | Fleischamann (2012) | Rheumatoid arthritis | Tofacitinib 5mg 6 months vs Tofacitinib 10mg 6 months vs Placebo 3 months + Tofacitinib 5mg 3 months vs Placebo 3 months + Tofacitinib 10mg 3 months | Industry | 97.4 (96.4 to 98.1) |
| **Vashisht RAIN (2016)** | Fleischamann (2012) | Rheumatoid arthritis | Tofacitinib 5mg 6 months vs Tofacitinib 10mg 6 months vs Placebo 3 months + Tofacitinib 5mg 3 months vs Placebo 3 months + Tofacitinib 10mg 3 months | Industry | 89.0 (87.3 to 90.4) |
| **Vashisht VARA (2016)** | van Vollenhoven (2012) | Rheumatoid arthritis | Tofacitinib 5mg vs Tofacitinib 10mg vs Adalimumab 40mg vs Placebo followed by Tofacitinib 5mg vs Placebo followed by Tofacitinib 10mg (all patients on background methotrexate) | Industry | 97.4 (96.4 to 98.1) |
| **Vashisht RAIN (2016)** | van Vollenhoven (2012) | Rheumatoid arthritis | Tofacitinib 5mg vs Tofacitinib 10mg vs Adalimumab SC 40mg vs Placebo followed by Tofacitinib 5mg vs Placebo followed by Tofacitinib 10mg (all patients on background methotrexate) | Industry | 89.6 (88.0 to 91.0) |

DMARD: Disease Modifying Anti-Rheumatic Drug

* Vashisht examines exclusion in 30 trials in two different clinical populations (Veterans’ Administration Rheumatoid Arthritis – VARA; and Rheumatoid Arthritis Investigators’ Network database– RAIN)

Supplementary Table S15: Trial-cohort comparisons included in the review for trials in HIV

| **Study** | **Name of trial (date of publication)** | **Condition examined** | **Treatment** | **Source of original trial funding** | **Percentage (95% CI) excluded** |
| --- | --- | --- | --- | --- | --- |
| **Gandhi (2005)**[**^16^**](#_ENREF_16) | ACTG 159 (1997) | HIV | Fluconazole + Itraconazole (both with and without prior course of Amphotericin B) | Public | 28.4 (26.3 to 30.5) |
| **Gandhi (2005)** | ACTG 206 (2002) | HIV | Interferon alfa-2b 1 million IU + Didanosine vs Interferon alfa-2b 10 million IU | Public | 50.9 (48.5 to 53.3) |
| **Gandhi (2005)** | ACTG 223 (2003) | HIV | Clarithromycin + Ethambutol vs Clarithromycin + Rifabutin vs Clarithromycin + Ethambutol + Rifabutin | Public | 34.9 (32.7 to 37.2) |
| **Gandhi (2005)** | ACTG 237 (2002) | HIV | Atovaquone + Pyrimethamine vs Atovaquone + Sulfadiazine | Public | 34.1 (31.9 to 36.3) |
| **Gandhi (2005)** | ACTG 243 (1998) | HIV | Antiretroviral therapy alone vs Antiretroviral therapy + Intravenous Cytarabine vs Antiretroviral therapy + Intrathecal Cytarabine | Industry | 37.2 (34.9 to 39.5) |
| **Gandhi (2005)** | ACTG 251 (1994) | HIV | Thalidomide vs Placebo | Industry | 43.2 (40.8 to 45.5) |
| **Gandhi (2005)** | ACTG 261 (1999) | HIV | Zidovudine + Didanosine + Delavirdine mesylate vs Zidovudine + Delavirdine mesylate vs Didanosine + Delavirdine mesylate vs Zidovudine + Didanosine | Public | 60.8 (58.5 to 63.1) |
| **Gandhi (2005)** | ACTG 306 (1999) | HIV | Stabudine Limbs: Stavudine + Placebo + Lamivudine vs Stavudine + Lamivudine vs Placebo + Zidovudine + Lamivudine; Didanosine Limbs: Didanosine + Placebo + Lamivudine vs Didanosine + Placebo + Lamivudine vs Placebo + Zidovudine + Lamivudine | Public | 43.0 (40.7 to 45.3) |
| **Gandhi (2005)** | ACTG 320 (1997) | HIV | Zidovudine + Lamivudine + Indinavir Sulfate vs Zidovudine + Lamivudine + Placebo | Public | 60.8 (58.5 to 63.1) |
| **Gandhi (2005)** | ACTG 343 (1998) | HIV | Zidovudine + Lamivudine + Indinavir vs Zidovudine + Lamivudine vs Indinavir | Public | 62.0 (59.6 to 64.2) |
| **Gandhi (2005)** | ACTG 347 (1999) | HIV | Amprenavir vs Amprenavir + Zidovudine + Lamivudine | Public | 51.2 (48.8 to 53.6) |
| **Gandhi (2005)** | ACTG 359 (2000) | HIV | Saquinavir + Ritonavir + Delavirdine vs Saquinavir + Ritonavir + Adefovir dipivoxil vs Saquinavir + Ritonavir + Delavirdine + Adefovir dipivoxil vs Saquinavir + Nelfinavir + Delavirdine vs Saquinavir + Nelfinavir + Adefovir dipivoxil vs Saquinavir + Nelfinavir + Delavirdine + Adefovir dipivoxil | Public | 55.7 (53.3 to 58.0) |
| **Gandhi (2005)** | ACTG 364 (2001) | HIV | Nelfinavir + Placebo vs Efavirenz + Placebo vs Nelfinavir + Efavirenz | Public | 55.6 (53.2 to 57.9) |
| **Gandhi (2005)** | ACTG 370 (2000) | HIV | Stavudine + Delavirdine + Indinavir vs Zidovudine + Lamivudine + Indinavir vs Zidovudine + Delavirdine + Indinavir | Public | 61.8 (59.5 to 64.1) |
| **Gandhi (2005)** | ACTG 373 (2001) | HIV | Amprenavir + Zidovudine/Stavudine + Lamivudine vs Indinavir + Nevirapine + Lamivudine + Stavudine vs Observation only | Public | 42.1 (39.7 to 44.4) |
| **Gandhi (2005)** | ACTG 388 (2003) | HIV | Lamivudine + Zidovudine + Indinavir vs Lamivudine + Zidovudine + Indinavir + Efavirenz vs Lamivudine + Zidovudine + Indinavir + Nelfinavir | Public | 49.6 (47.2 to 51.9) |
| **Gandhi (2005)** | ACTG 398 (2002) | HIV | Amprenavir + Saquinavir + Abacavir + Efavirenz + Adefovir vs Amprenavir + Indinavir + Abacavir + Efavirenz + Adefovir vs Amprenavir + Nelfinavir + Abacavir + Efavirenz + Adefovir vs Amprenavir + Abacavir + Efanavir + Adefovir | Public | 67.0 (64.7 to 69.2) |
| **Gandhi (2005)** | ACTG 384 (2003) | HIV | Didanosine + Stavudine + Efavirenz + Zidovudine + Lamivudine + Nelfinavir vs Didanosine + Stavudine + Nelfinavir + Zidovudine + Lamivudine + Efavirenz vs Zidovudine + Lamivudine + Efavirenz + Didanosine + Stavudine + Nelfinavir vs Zinovudine + Lamivudine + Nelfinavir + Didanosine + Stavudine + Efavirenz | Public | 67.6 (65.3 to 69.7) |
| **Gandhi (2005)** | ACTG 5095 (2001) | HIV | Zidovudine + Lamivudine + Efavirenz vs Zidovudine + Lamivudine + Abacavir + Efavirenz | Industry | 49.9 47.5 to 52.2) |
| **Gandhi (2005)** | CPCRA 002 (1994) | HIV | Didanosine vs Zalcitabine | Public | 41.8 (39.4 to 44.1) |
| **Gandhi (2005)** | CPCRA 004 (2000) | HIV | Isoniazid + Pyridoxine vs Rifampin + Pyrazinamide | Industry | 20.8 (18.9 to 22.8) |
| **Gandhi (2005)** | CPCRA 005 (1997) | HIV | Isoniazid vs Placebo | Public | 15.7 (14.0 to 17.5) |
| **Gandhi (2005)** | CPCRA 006 (1999) | HIV | Daily vs Thrice-weekly Trimethoprim-Sulfamethoxazole | Public | 4.4 (3.5 to 5.4) |
| **Gandhi (2005)** | CPCRA 007 (1996) | HIV | Zidovudine vs Zidovudine + Didanosine vs Zidovudine + Zalcitabine | Public | 38.9 (36.6 to 41.2) |
| **Gandhi (2005)** | CPCRA 009 (2000) | HIV | Clarithromycin vs Rifabutin vs Clarithromycin + Rifabutin | Public | 16.5 (14.8 to 18.3) |
| **Gandhi (2005)** | CPCRA 022 (1998) | HIV | Standardised point acupuncture + Amitriptyline vs Alternate point acupuncture + Amitriptyline vs Standardised Point acupuncture vs Alternate point acupuncture | Public | 12.8 (11.3 to 14.4) |
| **Gandhi (2005)** | CPCRA 023 (1998) | HIV | Ganciclovir vs Placebo | Industry | 7.2 (6.0 to 8.5) |
| **Gandhi (2005)** | CPCRA 048 (2000) | HIV | Azithromycin vs Placebo | Public | 0 (0.2 to 100) |
| **Gandhi (2005)** | CPCRA 064 (2003) | HIV | 4-month structured treatment interruption + Antiretroviral regimen vs Immediate initiation of antiretroviral regimen | Public | 25.8 (23.9 to 17.9) |
| **Gandhi (2005)** | CPCRA 046 (2000) | HIV | Genotypic antiretroviral resistance testing + suggested treatments vs No genotypic testing and no treatment suggestions | Public | 31.9 (29.7 to 34.1) |
| **Gandhi (2005)** | CPCRA 039 (2001) | HIV | Adefovir + L-carnitine vs Placebo + L-carnitine | Public | 8.2 (7.0 to 9.5) |

Supplementary Table S16: Trial-cohort comparisons included in the review for trials in ‘other’ conditions

| **Study** | **Name of trial (date of publication)** | **Condition examined** | **Treatment** | **Source of original trial funding** | **Percentage (95% CI) excluded** |
| --- | --- | --- | --- | --- | --- |
| **Bijkerk (2008)**[**^5^**](#_ENREF_5) | Bijkerk (2008) | Irritable bowel syndrome | Psyllium vs Bran vs Placebo | Not reported | 73.1 (71.2 to 75.0) |
| **Carter (2009)**[**^7^**](#_ENREF_7) | Arosio (2001) | Venous ulcers | Mesoglycan + Compression therapy + Topical wound care vs Placebo + Compression therapy + Topical wound care | Industry | 89.9 (87.0 to 90.6) |
| **Carter (2009)** | Conccheri (2002) | Venous ulcers | Sulodexide + Compression + Topical wound care vs Placebo + Compression + Topical wound care | Industry | 58.3 (55.4 to 61.1) |
| **Carter (2009)** | da Costa (1999) | Venous ulcers | Granulocyte-macrophage colony stimulating factor 200μg vs Granulocyte-macrophage colony stimulating factor 400μg vs Placebo | Not reported | 83.3 (81.1 to 85.3) |
| **Carter (2009)** | Harding (2005) | Venous ulcers | LyphoDerm + Standard care vs Standard care only | Not reported | 84.9 (82.8 to 86.9) |
| **Carter (2009)** | Milio (2005) | Venous ulcers | Intravenous Prostaglandin E1 + Elastic bandage + Local therapy vs Placebo + Elastic bandage + Local therapy | Industry | 86.6 (84.6 to 88.5) |
| **Carter (2009)** | Mostow (2005) | Venous ulcers | Weekly topical treatment of biomaterials from porcine small-intestine submucosa + Compression therapy vs Compression therapy alone | Not reported | 83.0 (80.7 to 85.0) |
| **Carter (2009)** | Robson (2004) | Venous ulcers | Topical Repifermin 60mcg/cm^2^ vs Topical Repifermin 120mcg/cm^2^ vs Placebo | Not reported | 83.6 (81.3 to 85.6) |
| **Carter (2009)**[**^7^**](#_ENREF_7) | Graumlich (2003) | Pressure ulcers | Topical collagen vs Topical hydrocolloid | Industry | 34.7 (31.7 to 37.9) |
| **Fischer (2012)**[**^13^**](#_ENREF_13) | INSECT (2009) | Incisional hernia | Interrupted suture (Vicryl) vs Continuous suture (Polydioxanone) vs Continuous suture (Monoplus) | Industry | 62.5 (58.6 to 66.2) |
| **Jones (2017)**[**^22^**](#_ENREF_22) | MUSICIAN (2012) | Fibromyalgia | Usual treatment only vs Telephone-delivered cognitive behavioral therapy + usual treatment vs Exercise + usual treatment vs Telephone-based cognitive behavioral therapy + exercise + usual treatment | Public | 52.1 (49.7 to 54.4) |
| **Markovic 4-month (2017)**[**^31^**](#_ENREF_31) | NCT02091453 (2014) | Brain injury | Attention Process Training vs Standard activity-based rehabilitation of attention | Public | 45.0 (41.1 to 49.0) |
| **Markovic 12-month (2017)**[**^31^**](#_ENREF_31) | NCT02091453 (2014) | Brain injury | Attention Process Training vs Standard activity-based rehabilitation of attention | Public | 35.9 (29.8 to 42.4) |
| **Morin-Ben Abdallah (2016)**[**^34^**](#_ENREF_34) | Belch (1981) | Venous Thromboembolism (prophylaxis) | Heparin vs No treatment | Not reported | 78.8 (70.1 to 85.6) |
| **Morin-Ben Abdallah (2016)** | Dahan (1986) | Venous Thromboembolism (prophylaxis) | Enoxaparin vs Placebo | Not reported | 42.4 (33.4 to 51.8) |
| **Morin-Ben Abdallah (2016)** | Gardlund (1996) | Venous Thromboembolism (prophylaxis) | Heparin vs No treatment | Public | 58.5 (49.0 to 67.3) |
| **Morin-Ben Abdallah (2016)** | Samama (1999) | Venous Thromboembolism (prophylaxis) | Enoxaparin 40mg vs Enoxaparin 20mg vs Placebo | Industry | 51.7 (42.4 to 60.9) |
| **Morin-Ben Abdallah (2016)** | Leizorovicz (2004) | Venous Thromboembolism (prophylaxis) | Dalteparin vs Placebo | Industry | 25.4 (18.1 to 34.4) |
| **Morin-Ben Abdallah (2016)** | Mahe (2005) | Venous Thromboembolism (prophylaxis) | Nadroparin vs Placebo | Industry | 23.7 (16.6 to 32.6) |
| **Morin-Ben Abdallah (2016)** | Cohen (2006) | Venous Thromboembolism (prophylaxis) | Fondaparinux vs Placebo | Industry | 24.6 (17.3 to 33.5) |
| **Morin-Ben Abdallah (2016)** | Lederle (2006) | Venous Thromboembolism (prophylaxis) | Enoxaparin vs Placebo | Not reported | 28.0 (20.3 to 37.1) |
| **Morin-Ben Abdallah (2016)** | Kakkar (2011) | Venous Thromboembolism (prophylaxis) | Enoxaparin vs Placebo | Industry | 41.5 (32.6 to 51.0) |
| **Schneider (1997)**[**^39^**](#_ENREF_39) | Knapp (1994) | Alzheimer’s Disease | Tacrine vs Placebo | Industry | 86.5 (85.0 to 88.0) |

Supplementary Table S17: Risk of bias by each of the three criteria

| **No. (%) of trial-clinical population pairs with each pattern N=305** | **Selection method for trials to examine** | **Choice of trial eligibility criteria to examine** | **Appropriateness of clinical population** | **Overall risk of bias** |
| --- | --- | --- | --- | --- |
| 126 (41.3) | **Low** | **Low** | **Low** | **Low** |
| 42 (13.8)  15 (4.9)  12 (3.9)  4 (1.3)  1 (0.3)  1 (0.3) | **Low**  **Low**  **Unclear**  **Unclear**  **Low**  **Unclear** | **Unclear**  **Unclear**  **Low**  **Unclear**  **Low**  **Low** | **Low**  **Unclear**  **Low**  **Low**  **Unclear**  **Unclear** | **Unclear** |
| 52 (17.0)  40 (13.1)  2 (0.7)  7 (2.3)  1 (0.3)  1 (0.3)  1 (0.3) | **Low**  **Low**  **Unclear**  **High**  **High**  **High**  **High** | **Low**  **Unclear**  **Low**  **Low**  **Low**  **Low**  **Unclear** | **High**  **High**  **High**  **Low**  **Unclear**  **High**  **High** | **High** |
| No (%) of trial-clinical population pairs for each risk of bias criterion N=305  **Low**  **Unclear**  **High** | 276 (90.5)  19 (6.2)  10 (3.3) | 203 (66.6)  102 (33.4)  0 | 191 (62.6)  18 (5.9)  96 (31.5) |  |

Supplementary Table S18: Risk of bias assessment for each trial-clinical population pair

| **Study** | **Name of trial (date of publication)** | **Condition examined** | **Risk of bias – selection of trials to examine** | **Risk of bias – appropriateness of the clinical population*** | **Risk of bias – choice of trial eligibility criteria to apply** |
| --- | --- | --- | --- | --- | --- |
| **Aaltonen (2017)**[**^2^**](#_ENREF_2) | Maini (1999) | Rheumatoid Arthritis | Low | High | Low |
| **Aaltonen (2017)** | Moreland (1999) | Rheumatoid Arthritis | Low | High | Low |
| **Aaltonen (2017)** | Weinblatt (1999) | Rheumatoid Arthritis | Low | High | Low |
| **Aaltonen (2017)** | Lipsky (2000) | Rheumatoid Arthritis | Low | High | Low |
| **Aaltonen (2017)** | ARMADA (2003) | Rheumatoid Arthritis | Low | High | Low |
| **Aaltonen (2017)** | TEMPO (2004) | Rheumatoid Arthritis | Low | High | Low |
| **Aaltonen (2017)** | Keystone (2004) | Rheumatoid Arthritis | Low | High | Low |
| **Aaltonen (2017)** | Van de Putte (2004) | Rheumatoid Arthritis | Low | High | Low |
| **Aaltonen (2017)** | Combe (2006) | Rheumatoid Arthritis | Low | High | Low |
| **Aaltonen (2017)** | TEMPO (2006) | Rheumatoid Arthritis | Low | High | Low |
| **Aaltonen (2017)** | Westhovens (2006) | Rheumatoid Arthritis | Low | High | Low |
| **Aaltonen (2017)** | Kim (2007) | Rheumatoid Arthritis | Low | High | Low |
| **Aaltonen (2017)** | Keystone (2008) | Rheumatoid Arthritis | Low | High | Low |
| **Aaltonen (2017)** | ATTEST (2008) | Rheumatoid Arthritis | Low | High | Low |
| **Aaltonen (2017)** | FAST4WARD (2009) | Rheumatoid Arthritis | Low | High | Low |
| **Aaltonen (2017)** | RAPID 2 (2009) | Rheumatoid Arthritis | Low | High | Low |
| **Aaltonen (2017)** | GO-AFTER (2009) | Rheumatoid Arthritis | Low | High | Low |
| **Aaltonen (2017)** | GO-FORWARD (2010) | Rheumatoid Arthritis | Low | High | Low |
| **Aaltonen (2017)** | Choy (2012) | Rheumatoid Arthritis | Low | High | Low |
| **Aaltonen (2017)** | REALISTIC (2012) | Rheumatoid Arthritis | Low | High | Low |
| **Aaltonen (2017)** | TEAR (2012) | Rheumatoid Arthritis | Low | High | Low |
| **Aaltonen (2017)** | GO-FORTH (2012) | Rheumatoid Arthritis | Low | High | Low |
| **Aaltonen (2017)** | ADACTA (2013) | Rheumatoid Arthritis | Low | High | Low |
| **Aaltonen (2017)** | AMPLE (2013) | Rheumatoid Arthritis | Low | High | Low |
| **Aaltonen (2017)** | O'Dell (2013) | Rheumatoid Arthritis | Low | High | Low |
| **Aaltonen (2017)** | Yoo (2013) | Rheumatoid Arthritis | Low | High | Low |
| **Andersson (2016)**[**^3^**](#_ENREF_3) | COLOFOL (2015) | Colorectal cancer | Low | Unclear | Low |
| **Bijker (2002)**[**^4^**](#_ENREF_4) | EORTC 10853 (2000) | Breast cancer (DCIS) | High | High | Low |
| **Bijkerk (2008)**[**^5^**](#_ENREF_5) | Bijkerk (2008) | Irritable bowel syndrome | High | Low | Low |
| **Bress (2017)**[**^6^**](#_ENREF_6) | SPRINT (2015) | Hypertension | High | Low | Low |
| **Carter (2009)**[**^7^**](#_ENREF_7) | Donahue (1998) | Diabetic foot ulcers | Low | Low | Unclear |
| **Carter (2009)** | Armstrong (2005) | Diabetic foot ulcers | Low | Low | Unclear |
| **Carter (2009)** | Driver (2006) | Diabetic foot ulcers | Low | Low | Unclear |
| **Carter (2009)** | Marston (2003) | Diabetic foot ulcers | Low | Low | Unclear |
| **Carter (2009)** | Kalani (2003) | Diabetic foot ulcers | Low | Low | Unclear |
| **Carter (2009)** | Tsang (2003) | Diabetic foot ulcers | Low | Low | Unclear |
| **Carter (2009)** | Veves (2002) | Diabetic foot ulcers | Low | Low | Unclear |
| **Carter (2009)**[**^7^**](#_ENREF_7) | Graumlich (2003) | Pressure ulcers | Low | Low | Unclear |
| **Carter (2009)**[**^7^**](#_ENREF_7) | Arosio (2001) | Venous ulcers | Low | Low | Unclear |
| **Carter (2009)** | Conccheri (2002) | Venous ulcers | Low | Low | Unclear |
| **Carter (2009)** | da Costa (1999) | Venous ulcers | Low | Low | Unclear |
| **Carter (2009)** | Harding (2005) | Venous ulcers | Low | Low | Unclear |
| **Carter (2009)** | Milio (2005) | Venous ulcers | Low | Low | Unclear |
| **Carter (2009)** | Mostow (2005) | Venous ulcers | Low | Low | Unclear |
| **Carter (2009)** | Robson (2004) | Venous ulcers | Low | Low | Unclear |
| **Chalmers (2016)**[**^8^**](#_ENREF_8) | EMBRACE (2012) | Bronchiectasis | Low | Low | Low |
| **Chalmers (2016)** | BLESS (2013) | Bronchiectasis | Low | Low | Low |
| **Chalmers (2016)** | BAT (2013) | Bronchiectasis | Low | Low | Low |
| **Chalmers (2016)** | AIR-BX (2014) | Bronchiectasis | Low | Low | Low |
| **Chalmers (2016)** | PROMIS (2014) | Bronchiectasis | Low | Low | Low |
| **Chalmers (2016)** | ORBIT-2 (2013) | Bronchiectasis | Low | Low | Low |
| **Chalmers (2016)** | RESPIRE (2013) | Bronchiectasis | Low | Low | Low |
| **Chalmers (2016)** | GENT (2011) | Bronchiectasis | Low | Low | Low |
| **Chalmers (2016)** | DNAse (1998) | Bronchiectasis | Low | Low | Low |
| **Chalmers (2016)** | B301 (2013) | Bronchiectasis | Low | Low | Low |
| **Constantino (2008)**[**^9^**](#_ENREF_9) | CIBIS (1994) | Heart failure | Low | Unclear | Unclear |
| **Constantino (2008)** | GESICA (1994) | Heart failure | Low | Unclear | Unclear |
| **Constantino (2008)** | Packer (1996) | Heart failure | Low | Unclear | Unclear |
| **Constantino (2008)** | DIG (1997) | Heart failure | Low | Unclear | Unclear |
| **Constantino (2008)** | PRIME II (1997) | Heart failure | Low | Unclear | Unclear |
| **Constantino (2008)** | FIRST (1997) | Heart failure | Low | Unclear | Unclear |
| **Constantino (2008)** | Cohn (1998) | Heart failure | Low | Unclear | Unclear |
| **Constantino (2008)** | RALES (1999) | Heart failure | Low | Unclear | Unclear |
| **Constantino (2008)** | DIAMOND (1999) | Heart failure | Low | Unclear | Unclear |
| **Constantino (2008)** | CIBIS II (1999) | Heart failure | Low | Unclear | Unclear |
| **Constantino (2008)** | MERIT-HF (2000) | Heart failure | Low | Unclear | Unclear |
| **Constantino (2008)** | MACH-1 (2000) | Heart failure | Low | Unclear | Unclear |
| **Constantino (2008)** | COPERNICUS (2001) | Heart failure | Low | Unclear | Unclear |
| **Constantino (2008)** | Val-HeFT (2001) | Heart failure | Low | Unclear | Unclear |
| **Constantino (2008)** | BEST (2001) | Heart failure | Low | Unclear | Unclear |
| **Constantino (2008)** | CHARM (2003) | Heart failure | Low | Unclear | Unclear |
| **Dalela (2017)**[**^10^**](#_ENREF_10) | PIVOT (2012) | Prostate cancer | Low | Low | Low |
| **Desmaele (2016)**[**^11^**](#_ENREF_11) | ARISTOTLE (2011) | Atrial fibrillation | High | High | Low |
| **Desmaele (2016)** | RE-LY (2011) | Atrial fibrillation | High | High | Low |
| **Desmaele (2016)** | ROCKET-AF (2011) | Atrial fibrillation | High | High | Low |
| **Fanning (2017)**[**^12^**](#_ENREF_12) | ARISTOTLE (2011) | Atrial fibrillation | High | Low | Low |
| **Fanning (2017)** | RE-LY (2011) | Atrial fibrillation | High | Low | Low |
| **Fanning (2017)** | ROCKET-AF (2011) | Atrial fibrillation | High | Low | Low |
| **Fischer (2012)**[**^13^**](#_ENREF_13) | INSECT (2009) | Incisional hernia | High | Low | Low |
| **Fortin (2006)**[**^14^**](#_ENREF_14) | Appel (2003) | Hypertension | Low | Low | Low |
| **Fortin (2006)** | Hansson (1998) | Hypertension | Low | Low | Low |
| **Fortin (2006)** | Wing (2003) | Hypertension | Low | Low | Low |
| **Fortin (2006)** | ALLHAT (2002) | Hypertension | Low | Low | Low |
| **Fortin (2006)** | Sacks (2001) | Hypertension | Low | Low | Low |
| **Fossa (2002)**[**^15^**](#_ENREF_15) | International Collaboration (1999) | Bladder cancer | Low | Low | Low |
| **Gandhi (2005)**[**^16^**](#_ENREF_16) | ACTG 159 (1997) | HIV | Low | Low | Low |
| **Gandhi (2005)** | ACTG 206 (2002) | HIV | Low | Low | Low |
| **Gandhi (2005)** | ACTG 223 (2003) | HIV | Low | Low | Low |
| **Gandhi (2005)** | ACTG 237 (2002) | HIV | Low | Low | Low |
| **Gandhi (2005)** | ACTG 243 (1998) | HIV | Low | Low | Low |
| **Gandhi (2005)** | ACTG 251 (1994) | HIV | Low | Low | Low |
| **Gandhi (2005)** | ACTG 261 (1999) | HIV | Low | Low | Low |
| **Gandhi (2005)** | ACTG 306 (1999) | HIV | Low | Low | Low |
| **Gandhi (2005)** | ACTG 320 (1997) | HIV | Low | Low | Low |
| **Gandhi (2005)** | ACTG 343 (1998) | HIV | Low | Low | Low |
| **Gandhi (2005)** | ACTG 347 (1999) | HIV | Low | Low | Low |
| **Gandhi (2005)** | ACTG 359 (2000) | HIV | Low | Low | Low |
| **Gandhi (2005)** | ACTG 364 (2001) | HIV | Low | Low | Low |
| **Gandhi (2005)** | ACTG 370 (2000) | HIV | Low | Low | Low |
| **Gandhi (2005)** | ACTG 373 (2001) | HIV | Low | Low | Low |
| **Gandhi (2005)** | ACTG 388 (2003) | HIV | Low | Low | Low |
| **Gandhi (2005)** | ACTG 398 (2002) | HIV | Low | Low | Low |
| **Gandhi (2005)** | ACTG 384 (2003) | HIV | Low | Low | Low |
| **Gandhi (2005)** | ACTG 5095 (2001) | HIV | Low | Low | Low |
| **Gandhi (2005)** | CPCRA 002 (1994) | HIV | Low | Low | Low |
| **Gandhi (2005)** | CPCRA 004 (2000) | HIV | Low | Low | Low |
| **Gandhi (2005)** | CPCRA 005 (1997) | HIV | Low | Low | Low |
| **Gandhi (2005)** | CPCRA 006 (1999) | HIV | Low | Low | Low |
| **Gandhi (2005)** | CPCRA 007 (1996) | HIV | Low | Low | Low |
| **Gandhi (2005)** | CPCRA 009 (2000) | HIV | Low | Low | Low |
| **Gandhi (2005)** | CPCRA 022 (1998) | HIV | Low | Low | Low |
| **Gandhi (2005)** | CPCRA 023 (1998) | HIV | Low | Low | Low |
| **Gandhi (2005)** | CPCRA 048 (2000) | HIV | Low | Low | Low |
| **Gandhi (2005)** | CPCRA 064 (2003) | HIV | Low | Low | Low |
| **Gandhi (2005)** | CPCRA 046 (2000) | HIV | Low | Low | Low |
| **Gandhi (2005)** | CPCRA 039 (2001) | HIV | Low | Low | Low |
| **Hagg (2014)**[**^17^**](#_ENREF_17) | ARISTOTLE (2011) | Atrial fibrillation | Low | Low | Low |
| **Halpin (2016)**[**^18^**](#_ENREF_18) | NCT02172287 (2000) | COPD | Low | Low | Low |
| **Halpin (2016)** | NCT00274014 (2003) | COPD | Low | Low | Low |
| **Halpin (2016)** | NCT00274547 (2003) | COPD | Low | Low | Low |
| **Halpin (2016)** | NCT00277264 (2004) | COPD | Low | Low | Low |
| **Halpin (2016)** | UPLIFT (2008) | COPD | Low | Low | Low |
| **Halpin (2016)** | Bateman (2010) | COPD | Low | Low | Low |
| **Halpin (2016)** | POET-COPD (2011) | COPD | Low | Low | Low |
| **Halpin (2016)** | TIOSPIR (2013) | COPD | Low | Low | Low |
| **Halpin (2016)** | Tie-COPD (2014) | COPD | Low | Low | Low |
| **Halpin (2016)** | NCT00134979 (2008) | COPD | Low | Low | Low |
| **Halpin (2016)** | ACCLAIM/COPD I (2011) | COPD | Low | Low | Low |
| **Halpin (2016)** | ATTAIN (2012) | COPD | Low | Low | Low |
| **Halpin (2016)** | NCT01044459 (2011) | COPD | Low | Low | Low |
| **Halpin (2016)** | Donohue (2010) | COPD | Low | Low | Low |
| **Halpin (2016)** | Jones (2011) | COPD | Low | Low | Low |
| **Halpin (2016)** | Yao (2014) | COPD | Low | Low | Low |
| **Halpin (2016)** | INVIGORATE (2013) | COPD | Low | Low | Low |
| **Halpin (2016)** | Ferguson (2014) | COPD | Low | Low | Low |
| **Halpin (2016)** | Koch (2014) | COPD | Low | Low | Low |
| **Halpin (2016)** | GLOW2 (2014) | COPD | Low | Low | Low |
| **Halpin (2016)** | GLOW1 (2014) | COPD | Low | Low | Low |
| **Halpin (2016)** | GLOW7 (2013) | COPD | Low | Low | Low |
| **Halpin (2016)** | SPARK (2013) | COPD | Low | Low | Low |
| **Halpin (2016)** | SHINE (2015) | COPD | Low | Low | Low |
| **Halpin (2016)** | ILLUMINATE (2013) | COPD | Low | Low | Low |
| **Halpin (2016)** | LANTERN (2015) | COPD | Low | Low | Low |
| **Halpin (2016)** | FLAME (2016) | COPD | Low | Low | Low |
| **Halpin (2016)** | Donohue (2013) | COPD | Low | Low | Low |
| **Halpin (2016)** | Decramer (2014) | COPD | Low | Low | Low |
| **Halpin (2016)** | NCT01777334 (2013) | COPD | Low | Low | Low |
| **Halpin (2016)** | Buhl (2015) | COPD | Low | Low | Low |
| **Hansen (2016)**[**^19^**](#_ENREF_19) | STICH-I (2005) | Stroke | Low | Low | Low |
| **Hansen (2016)** | CHANG (2007) | Stroke | Low | Low | Low |
| **Hansen (2016)** | Fast (2008) | Stroke | Low | Low | Low |
| **Hansen (2016)** | INTERACT (2013) | Stroke | Low | Low | Low |
| **Hansen (2016)** | STICH-II (2013) | Stroke | Low | Low | Low |
| **Hansen (2016)** | CLEAR-III (2014) | Stroke | Low | Low | Low |
| **Hansen (2016)** | ATACH-II (2016) | Stroke | Low | Low | Low |
| **Hansen (2016)** | MISTIE-III (2017) | Stroke | Low | Low | Low |
| **Hansen (2016)** | RESTART (2018) | Stroke | Low | Low | Low |
| **Hansen (2016)** | SWITCH (2018) | Stroke | Low | Low | Low |
| **Hansen (2016)** | TICH-2 (2018) | Stroke | Low | Low | Low |
| **Janson (2009)**[**^20^**](#_ENREF_20) | COLOR (2002) | Colon cancer | High | Low | Low |
| **Jeremias (2008)**[**^21^**](#_ENREF_21) | SIRIUS (2003) | Angina | Low | Low | Low |
| **Jones (2017)**[**^22^**](#_ENREF_22) | MUSICIAN | Chronic pain | High | High | Unclear |
| **Jost (2005)**[**^23^**](#_ENREF_23) | MERIT-HF (1999) | Heart failure | Low | Low | Low |
| **Klein (1995)**[**^24^**](#_ENREF_24) | DCCT (1993) | Type 1 diabetes | Low | Low | Low |
| **Klein (1995)** | DCCT (1993) | Type 1 diabetes | Low | Low | Low |
| **Kruis (2014)**[**^25^**](#_ENREF_25) | ISOLDE (2000) | COPD | Low | High | Low |
| **Kruis (2014)** | TRISTAN (2003) | COPD | Low | High | Low |
| **Kruis (2014)** | TORCH (2007) | COPD | Low | High | Low |
| **Kruis (2014)** | UPLIFT (2009) | COPD | Low | High | Low |
| **Kruis (2014)** | POET-COPD (2011) | COPD | Low | High | Low |
| **Krumhols CCP (2003)**[**^26^**](#_ENREF_26) | GUSTO (1993) | Acute myocardial infarction | Low | High | Low |
| **Krumhols NRRMI (2003)**[**^26^**](#_ENREF_26) | GUSTO (1993) | Acute myocardial infarction | Low | High | Low |
| **Lee (2012)**[**^27^**](#_ENREF_27) | RE-LY (2009) | Atrial fibrillation | Low | Low | Low |
| **Lee (2012)** | ARISTOTLE (2011) | Atrial fibrillation | Low | Low | Low |
| **Lee (2012)** | ROCKET-AF (2011) | Atrial fibrillation | Low | Low | Low |
| **Lloyd-Jones (2001)**[**^28^**](#_ENREF_28) | LPC-CPPT (1984) | Lipid lowering for primary prevention | Low | High | Unclear |
| **Lloyd-Jones (2001)** | HHS (1987) | Lipid lowering for primary prevention | Low | High | Unclear |
| **Lloyd-Jones (2001)** | WOSCOPS (1995) | Lipid lowering for primary prevention | Low | High | Unclear |
| **Lloyd-Jones (2001)** | AFCAPS/ TexCAPS (1998) | Lipid lowering for primary prevention | Low | High | Unclear |
| **Maasland (2009)**[**^29^**](#_ENREF_29) | ESPS-2 (1996) | Acute stroke/TIA | Low | Low | Low |
| **Maasland (2009)** | CARPIE (1996) | Acute stroke/TIA | Low | Low | Low |
| **Maasland (2009)** | TACIP (2003) | Acute stroke/TIA | Low | Low | Low |
| **Maasland (2009)** | MATCH (2004) | Acute stroke/TIA | Low | Low | Low |
| **Maasland (2009)** | ESPRIT (2006) | Acute stroke/TIA | Low | Low | Low |
| **Maasland (2009)** | PRoFESS (2008) | Acute stroke/TIA | Low | Low | Low |
| **Masoudi (2003)**[**^30^**](#_ENREF_30) | SOLVD (1991) | Heart failure | Low | High | Low |
| **Masoudi (2003)** | MERIT-HF (1999) | Heart failure | Low | High | Low |
| **Masoudi (2003)** | RALES (1999) | Heart failure | Low | High | Low |
| **Markovic 4-month (2017)**[**^31^**](#_ENREF_31) | NCT02091453 (2014) | Brain injury | High | Low | Low |
| **Markovic 12-month (2017)**[**^31^**](#_ENREF_31) | NCT02091453 (2014) | Brain injury | High | Low | Low |
| **Minnerup (2015)**[**^32^**](#_ENREF_32) | ALIAS 2 (2013) | Acute stroke | Unclear | Low | Unclear |
| **Minnerup (2015)** | AXIS 2 (2013) | Acute stroke | Unclear | Low | Unclear |
| **Minnerup (2015)** | EuroHYP-1 (2014) | Acute stroke | Unclear | Low | Unclear |
| **Minnerup (2015)** | SWIFT PRIME (2015) | Acute stroke | Unclear | Low | Unclear |
| **Miro (2015)**[**^33^**](#_ENREF_33) | RELAX-AHF (2013) | Acute heart failure | Unclear | Low | Low |
| **Morin-Ben Abdullah (2016)**[**^34^**](#_ENREF_34) | Belch (1981) | Venous thromboembolism prophylaxis | Low | Low | Low |
| **Morin-Ben Abdullah (2016)** | Dahan (1986) | Venous thromboembolism prophylaxis | Low | Low | Low |
| **Morin-Ben Abdullah (2016)** | Gardlund (1996) | Venous thromboembolism prophylaxis | Low | Low | Low |
| **Morin-Ben Abdullah (2016)** | Samama (1999) | Venous thromboembolism prophylaxis | Low | Low | Low |
| **Morin-Ben Abdullah (2016)** | Leizorovicz (2004) | Venous thromboembolism prophylaxis | Low | Low | Low |
| **Morin-Ben Abdullah (2016)** | Mahe (2005) | Venous thromboembolism prophylaxis | Low | Low | Low |
| **Morin-Ben Abdullah (2016)** | Cohen (2006) | Venous thromboembolism prophylaxis | Low | Low | Low |
| **Morin-Ben Abdullah (2016)** | Lederle (2006) | Venous thromboembolism prophylaxis | Low | Low | Low |
| **Morin-Ben Abdullah (2016)** | Kakkar (2011) | Venous thromboembolism prophylaxis | Low | Low | Low |
| **Patel (2017)**[**^35^**](#_ENREF_35) | DIG-Ancillary (1997) | Heart failure | Unclear | Low | Low |
| **Patel (2017)** | CHARM-Preserved (2003) | Heart failure | Unclear | Low | Low |
| **Patel (2017)** | PEP-CHF (2006) | Heart failure | Unclear | Low | Low |
| **Patel (2017)** | I-PRESERVE (2008) | Heart failure | Unclear | Low | Low |
| **Patel (2017)** | J-DHF (2013) | Heart failure | Unclear | Low | Low |
| **Patel (2017)** | TOPCAT (2014) | Heart failure | Unclear | Low | Low |
| **Patel (2017)** | PARAGON-HF (2018) | Heart failure | Unclear | Low | Low |
| **Pedone (2003)**[**^36^**](#_ENREF_36) | VA-NHBLI (1978) | Hypertension | Low | High | Low |
| **Pedone (2003)** | HDPF (1979) | Hypertension | Low | High | Low |
| **Pedone (2003)** | Oslo (1980) | Hypertension | Low | High | Low |
| **Pedone (2003)** | Australia (1980) | Hypertension | Low | High | Low |
| **Pedone (2003)** | MRC (1985) | Hypertension | Low | High | Low |
| **Pedone (2003)** | VA I (1967) | Hypertension | Low | High | Low |
| **Pedone (2003)** | VA II (1970) | Hypertension | Low | High | Low |
| **Pedone (2003)** | PHS (1977) | Hypertension | Low | High | Low |
| **Pedone (2003)** | HSCSG (1977) | Hypertension | Low | High | Low |
| **Pedone (2003)** | Barraclough (1971) | Hypertension | Low | High | Low |
| **Pedone (2003)** | Carter (1970) | Hypertension | Low | High | Low |
| **Pedone (2003)** | EWPHE (1985) | Hypertension | Low | High | Low |
| **Pedone (2003)** | Coope (1986) | Hypertension | Low | High | Low |
| **Pedone (2003)** | MRC-O (1992) | Hypertension | Low | High | Low |
| **Pedone (2003)** | SHEP (1991) | Hypertension | Low | High | Low |
| **Pedone (2003)** | STOP (1991) | Hypertension | Low | High | Low |
| **Neider (2017)**[**^37^**](#_ENREF_37) | Motzer (2013) | Metastatic renal cancer | Unclear | Low | Low |
| **Neider (2017)**[**^37^**](#_ENREF_37) | Choueiri (2015) | Metastatic renal cancer | Unclear | Low | Low |
| **Neider (2017)**[**^37^**](#_ENREF_37) | Motzer (2015) | Metastatic renal cancer | Unclear | Low | Low |
| **Saunders (2013)**[**^38^**](#_ENREF_38) | ACCORD (2008) | Type 2 diabetes | Low | Low | Low |
| **Saunders (2013)** | ADVANCE (2008) | Type 2 diabetes | Low | Low | Low |
| **Saunders (2013)** | PROactive (2005) | Type 2 diabetes | Low | Low | Low |
| **Saunders (2013)** | RECORD (2009) | Type 2 diabetes | Low | Low | Low |
| **Saunders (2013)** | VADT (2009) | Type 2 diabetes | Low | Low | Low |
| **Saunders (2013)** | UKDPS 33 (1998) | Type 2 diabetes | Low | Low | Low |
| **Saunders (2013)** | UKDPS 34 (1998) | Type 2 diabetes | Low | Low | Low |
| **Schneider (1997)**[**^39^**](#_ENREF_39) | Knapp (1994) | Alzheimer’s Disease | High | Unclear | Low |
| **Sokka (2003)**[**^40^**](#_ENREF_40) | ATTRACT (2002) | Rheumatoid arthritis | Unclear | Unclear | Low |
| **Spitzer (2016)**[**^41^**](#_ENREF_41) | COMFORTABLE AMI (2013) | Myocardial infarction | High | Low | Low |
| **Terschuren (2010)**[**^42^**](#_ENREF_42) | NHL-B1/NHL-B2 (2004) | Lymphoma | Unclear | Low | Low |
| **Timmis (2016)**[**^43^**](#_ENREF_43) | PEGASUS-TIMI-54 (2015) | Secondary prevention after myocardial infarction | Unclear | High | Low |
| **Travers (2007)**[**^44^**](#_ENREF_44) | Greening (1994) | Asthma | Low | High | Unclear |
| **Travers (2007)** | Woolcock (1996) | Asthma | Low | High | Unclear |
| **Travers (2007)** | Pauwels (1994) | Asthma | Low | High | Unclear |
| **Travers (2007)** | Busse (1998) | Asthma | Low | High | Unclear |
| **Travers (2007)** | Reed (1998) | Asthma | Low | High | Unclear |
| **Travers (2007)** | Wenzel (1998) | Asthma | Low | High | Unclear |
| **Travers (2007)** | Laviolette (1999) | Asthma | Low | High | Unclear |
| **Travers (2007)** | Bleecker (2000) | Asthma | Low | High | Unclear |
| **Travers (2007)** | Nelson (2000) | Asthma | Low | High | Unclear |
| **Travers (2007)** | Busse (2001) | Asthma | Low | High | Unclear |
| **Travers (2007)** | Fish (2001) | Asthma | Low | High | Unclear |
| **Travers (2007)** | O'Byrne (2001) | Asthma | Low | High | Unclear |
| **Travers (2007)** | Lalloo (2003) | Asthma | Low | High | Unclear |
| **Travers (2007)** | Pauwels (2003) | Asthma | Low | High | Unclear |
| **Travers (2007)** | Price (2003) | Asthma | Low | High | Unclear |
| **Travers (2007)** | Ringdal (2003) | Asthma | Low | High | Unclear |
| **Travers (2007)** | Vaquerizo (2003) | Asthma | Low | High | Unclear |
| **Travers (2007)**[**^45^**](#_ENREF_45) | Anthonisen (1994) | COPD | Low | High | Unclear |
| **Travers (2007)** | COMVIBENT (1997) | COPD | Low | High | Unclear |
| **Travers (2007)** | Boyd (1997) | COPD | Low | High | Unclear |
| **Travers (2007)** | Gross (1998) | COPD | Low | High | Unclear |
| **Travers (2007)** | Mahler (1999) | COPD | Low | High | Unclear |
| **Travers (2007)** | Paulwels (1999) | COPD | Low | High | Unclear |
| **Travers (2007)** | Lung Health Study Research Group (2000) | COPD | Low | High | Unclear |
| **Travers (2007)** | Burge (2000) | COPD | Low | High | Unclear |
| **Travers (2007)** | Dahl (2001) | COPD | Low | High | Unclear |
| **Travers (2007)** | Casaburi (2002) | COPD | Low | High | Unclear |
| **Travers (2007)** | Mahler (2002) | COPD | Low | High | Unclear |
| **Travers (2007)** | Rossi (2002) | COPD | Low | High | Unclear |
| **Travers (2007)** | Vincken (2002) | COPD | Low | High | Unclear |
| **Travers (2007)** | Calverley (2003) | COPD | Low | High | Unclear |
| **Travers (2007)** | Calverley (2003) | COPD | Low | High | Unclear |
| **Travers (2007)** | Hanania (2003) | COPD | Low | High | Unclear |
| **Travers (2007)** | Szafranski (2003) | COPD | Low | High | Unclear |
| **Treweek (2005)**[**^46^**](#_ENREF_46) | ATAC (2002) | Breast cancer | Low | Low | Low |
| **Treweek (2005)** | BIG 1-98 (2005) | Breast cancer | Low | Low | Low |
| **Treweek (2015)** | CANADIAN (2002) | Breast cancer | Low | Low | Low |
| **Treweek (2015)** | EORTC (2001) | Breast cancer | Low | Low | Low |
| **Treweek (2015)** | IES (2004) | Breast cancer | Low | Low | Low |
| **Treweek (2015)** | MA17 (2003) | Breast cancer | Low | Low | Low |
| **Treweek (2015)** | NSABP B-28 (2005) | Breast cancer | Low | Low | Low |
| **Treweek (2015)** | START A/ START B (2008) | Breast cancer | Low | Low | Low |
| **Treweek (2015)** | TACT (2009) | Breast cancer | Low | Low | Low |
| **Treweek (2015)** | TARGIT-A (2010) | Breast cancer | Low | Low | Low |
| **Treweek (2015)** | TEAM (2011) | Breast cancer | Low | Low | Low |
| **Vardy (2009)**[**^47^**](#_ENREF_47) | E1594 (2001) | Non-small cell lung cancer | Low | Low | Unclear |
| **Vardy (2009)** | SWOG 9509 (2001) | Non-small cell lung cancer | Low | Low | Unclear |
| **Vardy (2009)** | TAS 326 (2003) | Non-small cell lung cancer | Low | Low | Unclear |
| **Vashisht VARA (2016)**[**^48^**](#_ENREF_48) | Moreland (1999) | Rheumatoid arthritis | Low | High | Unclear |
| **Vashisht VARA (2016)** | Maini (1999) | Rheumatoid arthritis | Low | High | Unclear |
| **Vashisht VARA (2016)** | St Clair (2004) | Rheumatoid arthritis | Low | High | Unclear |
| **Vashisht VARA (2016)** | Weinblatt (2003) | Rheumatoid arthritis | Low | High | Unclear |
| **Vashisht VARA (2016)** | Furst (2003) | Rheumatoid arthritis | Low | High | Unclear |
| **Vashisht VARA (2016)** | Keystone (2004) | Rheumatoid arthritis | Low | High | Unclear |
| **Vashisht VARA (2016)** | Van de Putte (2004) | Rheumatoid arthritis | Low | High | Unclear |
| **Vashisht VARA (2016)** | Keystone (2008) | Rheumatoid arthritis | Low | High | Unclear |
| **Vashisht VARA (2016)** | Smolen (2009) | Rheumatoid arthritis | Low | High | Unclear |
| **Vashisht VARA (2016)** | Fleischmann (2009) | Rheumatoid arthritis | Low | High | Unclear |
| **Vashisht VARA (2016)** | Keystone (2009) | Rheumatoid arthritis | Low | High | Unclear |
| **Vashisht VARA (2016)** | Emery (2009) | Rheumatoid arthritis | Low | High | Unclear |
| **Vashisht VARA (2016)** | Smolen (2012) | Rheumatoid arthritis | Low | High | Unclear |
| **Vashisht VARA (2016)** | Bresnihan (1998) | Rheumatoid arthritis | Low | High | Unclear |
| **Vashisht VARA (2016)** | Cohen (2002) | Rheumatoid arthritis | Low | High | Unclear |
| **Vashisht VARA (2016)** | Fleischmann (2003) | Rheumatoid arthritis | Low | High | Unclear |
| **Vashisht VARA (2016)** | Cohen (2004) | Rheumatoid arthritis | Low | High | Unclear |
| **Vashisht VARA (2016)** | Edwards (2004) | Rheumatoid arthritis | Low | High | Unclear |
| **Vashisht VARA (2016)** | Cohen (2006) | Rheumatoid arthritis | Low | High | Unclear |
| **Vashisht VARA (2016)** | Genovese (2005) | Rheumatoid arthritis | Low | High | Unclear |
| **Vashisht VARA (2016)** | Kremer (2006) | Rheumatoid arthritis | Low | High | Unclear |
| **Vashisht VARA (2016)** | Westhovens (2009) | Rheumatoid arthritis | Low | High | Unclear |
| **Vashisht VARA (2016)** | Genovese (2008) | Rheumatoid arthritis | Low | High | Unclear |
| **Vashisht VARA (2016)** | Emery (2008) | Rheumatoid arthritis | Low | High | Unclear |
| **Vashisht VARA (2016)** | Smolen (2008) | Rheumatoid arthritis | Low | High | Unclear |
| **Vashisht VARA (2016)** | Jones (2010) | Rheumatoid arthritis | Low | High | Unclear |
| **Vashisht VARA (2016)** | Kremer (2011) | Rheumatoid arthritis | Low | High | Unclear |
| **Vashisht VARA (2016)** | Burmester (2013) | Rheumatoid arthritis | Low | High | Unclear |
| **Vashisht VARA (2016)** | Fleischamann (2012) | Rheumatoid arthritis | Low | High | Unclear |
| **Vashisht VARA (2016)** | van Vollenhoven (2012) | Rheumatoid arthritis | Low | High | Unclear |
| **Vashisht RAIN (2016)**[**^48^**](#_ENREF_48) | Moreland (1999) | Rheumatoid arthritis | Low | Low | Unclear |
| **Vashisht RAIN (2016)** | Maini (1999) | Rheumatoid arthritis | Low | Low | Unclear |
| **Vashisht RAIN (2016)** | St Clair (2004) | Rheumatoid arthritis | Low | Low | Unclear |
| **Vashisht RAIN (2016)** | Weinblatt (2003) | Rheumatoid arthritis | Low | Low | Unclear |
| **Vashisht RAIN (2016)** | Furst (2003) | Rheumatoid arthritis | Low | Low | Unclear |
| **Vashisht RAIN (2016)** | Keystone (2004) | Rheumatoid arthritis | Low | Low | Unclear |
| **Vashisht RAIN (2016)** | Van de Putte (2004) | Rheumatoid arthritis | Low | Low | Unclear |
| **Vashisht RAIN (2016)** | Keystone (2008) | Rheumatoid arthritis | Low | Low | Unclear |
| **Vashisht RAIN (2016)** | Smolen (2009) | Rheumatoid arthritis | Low | Low | Unclear |
| **Vashisht RAIN (2016)** | Fleischmann (2009) | Rheumatoid arthritis | Low | Low | Unclear |
| **Vashisht RAIN (2016)** | Keystone (2008) | Rheumatoid arthritis | Low | Low | Unclear |
| **Vashisht RAIN (2016)** | Emery (2009) | Rheumatoid arthritis | Low | Low | Unclear |
| **Vashisht RAIN (2016)** | Smolen (2012) | Rheumatoid arthritis | Low | Low | Unclear |
| **Vashisht RAIN (2016)** | Bresnihan (1998) | Rheumatoid arthritis | Low | Low | Unclear |
| **Vashisht RAIN (2016)** | Cohen (2002) | Rheumatoid arthritis | Low | Low | Unclear |
| **Vashisht RAIN (2016)** | Fleischmann (2003) | Rheumatoid arthritis | Low | Low | Unclear |
| **Vashisht RAIN (2016)** | Cohen (2004) | Rheumatoid arthritis | Low | Low | Unclear |
| **Vashisht RAIN (2016)** | Edwards (2004) | Rheumatoid arthritis | Low | Low | Unclear |
| **Vashisht RAIN (2016)** | Cohen (2006) | Rheumatoid arthritis | Low | Low | Unclear |
| **Vashisht RAIN (2016)** | Genovese (2005) | Rheumatoid arthritis | Low | Low | Unclear |
| **Vashisht RAIN (2016)** | Kremer (2006) | Rheumatoid arthritis | Low | Low | Unclear |
| **Vashisht RAIN (2016)** | Westhovens (2009) | Rheumatoid arthritis | Low | Low | Unclear |
| **Vashisht RAIN (2016)** | Genovese (2008) | Rheumatoid arthritis | Low | Low | Unclear |
| **Vashisht RAIN (2016)** | Emery (2008) | Rheumatoid arthritis | Low | Low | Unclear |
| **Vashisht RAIN (2016)** | Smolen (2008) | Rheumatoid arthritis | Low | Low | Unclear |
| **Vashisht RAIN (2016)** | Jones (2010) | Rheumatoid arthritis | Low | Low | Unclear |
| **Vashisht RAIN (2016)** | Kremer (2011) | Rheumatoid arthritis | Low | Low | Unclear |
| **Vashisht RAIN (2016)** | Burmester (2013) | Rheumatoid arthritis | Low | Low | Unclear |
| **Vashisht RAIN (2016)** | Fleischamann (2012) | Rheumatoid arthritis | Low | Low | Unclear |
| **Vashisht RAIN (2016)** | van Vollenhoven (2012) | Rheumatoid arthritis | Low | Low | Unclear |
| **Ward (1992)**[**^49^**](#_ENREF_49) | BSCG-1 (1989) | Stomach cancer | Unclear | High | Low |
| **Yeh (2015)**[**^50^**](#_ENREF_50) | DAPT (2010) | Angina | Low | Low | Low |
| **Yoon (2014)**[**^51^**](#_ENREF_51) | RE-LY (2009) | Atrial fibrillation | Low | High | Low |
| **Yoon (2014)** | ROCKET-AF (2011) | Atrial fibrillation | Low | High | Low |
| **Yoon (2014)** | ARISTOTLE (2011) | Atrial fibrillation | Low | High | Low |
| **Yoon (2014)** | ENGAGE (2013) | Atrial fibrillation | Low | High | Low |

### Additional file references

1. A systematic review of the applicability of clinical trial evidence to real-world patients. PROSPERO 2016:CRD42016042282 Available from <http://www.crd.york.ac.uk/PROSPERO/display_record.asp?ID=CRD42016042282>. 2016.

2. Aaltonen KJ, Ylikylä S, Tuulikki Joensuu J, et al. Efficacy and effectiveness of tumour necrosis factor inhibitors in the treatment of rheumatoid arthritis in randomized controlled trials and routine clinical practice. Rheumatology 2017;56:725-35.

3. Andersson PA, Wille-Jorgensen P, Horvath-Puho E, et al. The COLOFOL trial: study design and comparison of the study population with the source cancer population. Clin Epidemiol 2016;8:15-21.

4. Bijker N, Peterse JL, Fentiman IS, et al. Effects of patient selection on the applicability of results from a randomised clinical trial (EORTC 10853) investigating breast-conserving therapy for DCIS. Br J Cancer 2002;87:615-20.

5. Bijkerk CJ, Muris JW, Knottnerus JA, Hoes AW, de Wit NJ. Randomized patients in IBS research had different disease characteristics compared to eligible and recruited patients. J Clin Epidemiol 2008;61:1176-81.

6. Bress AP, Kramer H, Khatib R, et al. Potential Deaths Averted and Serious Adverse Events Incurred From Adoption of the SPRINT (Systolic Blood Pressure Intervention Trial) Intensive Blood Pressure Regimen in the United States: Projections From NHANES (National Health and Nutrition Examination Survey). Circulation 2017;135:1617-28.

7. Carter MJ, Fife CE, Walker D, Thomson B. Estimating the applicability of wound care randomized controlled trials to general wound-care populations by estimating the percentage of individuals excluded from a typical wound-care population in such trials. Advances in skin & wound care 2009;22:316-24.

8. Chalmers JD, McDonnell MJ, Rutherford R, et al. The generalizability of bronchiectasis randomized controlled trials: A multicentre cohort study. Respir Med 2016;112:51-8.

9. Costantino G, Rusconi AM, Duca PG, et al. Eligibility criteria in heart failure randomized controlled trials: a gap between evidence and clinical practice. Intern Emerg Med 2009;4:117-22.

10. Dalela D, Karabon P, Sammon J, et al. Generalizability of the Prostate Cancer Intervention Versus Observation Trial (PIVOT) Results to Contemporary North American Men with Prostate Cancer. European urology 2017;71:511-4.

11. Desmaele S, Steurbaut S, Cornu P, Brouns R, Dupont AG. Clinical trials with direct oral anticoagulants for stroke prevention in atrial fibrillation: how representative are they for real life patients? European journal of clinical pharmacology 2016;72:1125-34.

12. Fanning L, Ilomaki J, Bell JS, Darzins P. The representativeness of direct oral anticoagulant clinical trials to hospitalized patients with atrial fibrillation. European journal of clinical pharmacology 2017;73:1427-36.

13. Fischer L, Knaebel HP, Golcher H, et al. To whom do the results of the multicenter, randomized, controlled INSECT trial (ISRCTN 24023541) apply?-assessment of external validity. BMC surgery 2012;12:2.

14. Fortin M, Dionne J, Pinho G, Gignac J, Almirall J, Lapointe L. Randomized controlled trials: do they have external validity for patients with multiple comorbidities? Ann Fam Med 2006;4:104-8.

15. Fosså SD, Skovlund E. Selection of Patients may Limit the Generalizability of Results from Cancer Trials. Acta Oncologica 2009;41:131-7.

16. Gandhi M, Ameli N, Bacchetti P, et al. Eligibility criteria for HIV clinical trials and generalizability of results: the gap between published reports and study protocols. Aids 2005;19:1885-96.

17. Hagg L, Johansson C, Jansson JH, Johansson L. External validity of the ARISTOTLE trial in real-life atrial fibrillation patients. Cardiovasc Ther 2014;32:214-8.

18. Halpin DM, Kerkhof M, Soriano JB, Mikkelsen H, Price DB. Eligibility of real-life patients with COPD for inclusion in trials of inhaled long-acting bronchodilator therapy. Respir Res 2016;17:120.

19. Hansen BM, Ullman N, Norrving B, Hanley DF, Lindgren A. Applicability of Clinical Trials in an Unselected Cohort of Patients With Intracerebral Hemorrhage. Stroke 2016;47:2634-7.

20. Janson M, Edlund G, Kressner U, et al. Analysis of patient selection and external validity in the Swedish contribution to the COLOR trial. Surg Endosc 2009;23:1764-9.

21. Jeremias A, Ruisi CP, Kirtane AJ, et al. Differential outcomes after sirolimus-eluting stent implantation: comparing on-label versus off-label patients in the ‘real world’. Coronary artery disease 2008;19:111-5.

22. Jones GT, Jones EA, Beasley MJ, Macfarlane GJ. Investigating generalizability of results from a randomized controlled trial of the management of chronic widespread pain: the MUSICIAN study. Pain 2017;158:96-102.

23. Jost A, Rauch B, Hochadel M, et al. Beta-blocker treatment of chronic systolic heart failure improves prognosis even in patients meeting one or more exclusion criteria of the MERIT-HF study. Eur Heart J 2005;26:2689-97.

24. Klein R, Moss S. A comparison of the study populations in the Diabetes Control and Complications Trial and the Wisconsin Epidemiologic Study of Diabetic Retinopathy. Archives of internal medicine 1995;155:745-54.

25. Kruis AL, Stallberg B, Jones RC, et al. Primary care COPD patients compared with large pharmaceutically-sponsored COPD studies: an UNLOCK validation study. PLoS One 2014;9:e90145.

26. Krumholz HM, Gross CP, Peterson ED, et al. Is there evidence of implicit exclusion criteria for elderly subjects in randomized trials? Evidence from the GUSTO-1 study. American Heart Journal 2003;146:839-47.

27. Lee S, Monz BU, Clemens A, Brueckmann M, Lip GY. Representativeness of the dabigatran, apixaban and rivaroxaban clinical trial populations to real-world atrial fibrillation patients in the United Kingdom: a cross-sectional analysis using the General Practice Research Database. BMJ Open 2012;2.

28. Lloyd-Jones DM, O'donnell CJ, D'agostino RB, Massaro J, Silbershatz H, Wilson PW. Applicability of cholesterol-lowering primary prevention trials to a general population: the Framingham Heart Study. Archives of internal medicine 2001;161:949-54.

29. Maasland L, van Oostenbrugge RJ, Franke CF, et al. Patients enrolled in large randomized clinical trials of antiplatelet treatment for prevention after transient ischemic attack or ischemic stroke are not representative of patients in clinical practice: the Netherlands Stroke Survey. Stroke 2009;40:2662-8.

30. Masoudi FA, Havranek EP, Wolfe P, et al. Most hospitalized older persons do not meet the enrollment criteria for clinical trials in heart failure. American Heart Journal 2003;146:250-7.

31. Markovic G, Schult ML, Bartfai A. The effect of sampling bias on generalizability in intervention trials after brain injury. Brain Inj 2017;31:9-15.

32. Minnerup J, Trinczek B, Storck M, et al. Feasibility platform for stroke studies: an online tool to improve eligibility criteria for clinical trials. Stroke 2015;46:137-42.

33. Miro O, Gil V, Muller C, et al. How does a clinical trial fit into the real world? The RELAX-AHF study population into the EAHFE registry. Clin Res Cardiol 2015;104:850-60.

34. Morin-Ben Abdallah S, Dutilleul A, Nadon V, et al. Quantification of the External Validity of Randomized Controlled Trials Supporting Clinical Care Guidelines: The Case of Thromboprophylaxis. Am J Med 2016;129:740-5.

35. Patel HC, Hayward C, Dungu JN, et al. Assessing the Eligibility Criteria in Phase III Randomized Controlled Trials of Drug Therapy in Heart Failure With Preserved Ejection Fraction: The Critical Play-Off Between a “Pure” Patient Phenotype and the Generalizability of Trial Findings. Journal of Cardiac Failure 2017;23:517-24.

36. Pedone C, Lapane KL. Generalizability of guidelines and physicians' adherence. Case study on the Sixth Joint National Commitee's guidelines on hypertension. BMC public health 2003;3:24.

37. Nieder C, Syed MA, Dalhaug A, Pawinski A, Norum J. Eligibility for phase 3 clinical trials of systemic therapy in real-world patients with metastatic renal cell cancer managed in a rural region. Medical Oncology 2017;34:149.

38. Saunders C, Byrne CD, Guthrie B, et al. External validity of randomized controlled trials of glycaemic control and vascular disease: how representative are participants? Diabet Med 2013;30:300-8.

39. Schneider LS, Olin JT, Lyness SA, Chui HC. Eligibility of Alzheimer's disease clinic patients for clinical trials. Journal of the American Geriatrics Society 1997;45:923-8.

40. Sokka T, Pincus T. Eligibility of patients in routine care for major clinical trials of anti-tumor necrosis factor alpha agents in rheumatoid arthritis. Arthritis Rheum 2003;48:313-8.

41. Spitzer E, Hadorn S, Zanchin T, et al. External validity of a contemporaneous primary percutaneous coronary intervention trial in patients with acute ST-elevation myocardial infarction: insights from a single-centre investigation. EuroIntervention 2016;12:1135-43.

42. Terschuren C, Gierer S, Brillant C, Paulus U, Loffler M, Hoffmann W. Are patients with Hodgkin lymphoma and high-grade non-Hodgkin lymphoma in clinical therapy optimization protocols representative of these groups of patients in Germany? Ann Oncol 2010;21:2045-51.

43. Timmis A, Rapsomaniki E, Chung SC, et al. Prolonged dual antiplatelet therapy in stable coronary disease: comparative observational study of benefits and harms in unselected versus trial populations. BMJ 2016;353:i3163.

44. Travers J, Marsh S, Williams M, et al. External validity of randomised controlled trials in asthma: to whom do the results of the trials apply? Thorax 2007;62:219-23.

45. Travers J, Marsh S, Caldwell B, et al. External validity of randomized controlled trials in COPD. Respir Med 2007;101:1313-20.

46. Treweek S, Dryden R, McCowan C, Harrow A, Thompson AM. Do participants in adjuvant breast cancer trials reflect the breast cancer patient population? Eur J Cancer 2015;51:907-14.

47. Vardy J, Dadasovich R, Beale P, Boyer M, Clarke SJ. Eligibility of patients with advanced non-small cell lung cancer for phase III chemotherapy trials. BMC Cancer 2009;9:130.

48. Vashisht P, Sayles H, Cannella AC, Mikuls TR, Michaud K. Generalizability of Patients With Rheumatoid Arthritis in Biologic Agent Clinical Trials. Arthritis Care Res (Hoboken) 2016;68:1478-88.

49. Ward L, Fielding J, Dunn J, Kelly K. The selection of cases for randomised trials: a registry survey of concurrent trial and non-trial patients. The British Stomach Cancer Group. British journal of cancer 1992;66:943.

50. Yeh RW, Czarny MJ, Normand SL, et al. Evaluating the generalizability of a large streamlined cardiovascular trial: comparing hospitals and patients in the dual antiplatelet therapy study versus the National Cardiovascular Data Registry. Circ Cardiovasc Qual Outcomes 2015;8:96-102.

51. Yoon CH, Park YK, Kim SJ, et al. Eligibility and preference of new oral anticoagulants in patients with atrial fibrillation: comparison between patients with versus without stroke. Stroke 2014;45:2983-8.
